# Synthesis and Evaluation of Colchicine C-Cyclic AmineDerivatives as Potent Anti-Biofilms Agents AgainstMethicillin-Resistant *Staphylococcus aureus*

**DOI:** 10.3390/antibiotics14020173

**Published:** 2025-02-10

**Authors:** Yuxin Yang, Xin Liu, Can Sun, Yuan Fang, Danyang Qu, Zhengbin Tang, Zetao Sun, Xiaoping Zhou, Dacheng Wang

**Affiliations:** 1 College of Animal Science, Jilin University, Changchun 130062, China; yangyuxin22@mails.jlu.edu.cn (Y.Y.); l_xin22@mails.jlu.edu.cn (X.L.); suncan24@mails.jlu.edu.cn (C.S.); 13763534150@163.com (D.Q.); sunzt24@mails.jlu.edu.cn (Z.S.); 2 School of Pharmaceutical Science, Jilin University, Changchun 130021, China; fangyuan23@mails.jlu.edu.cn (Y.F.); tangzb24@mails.jlu.edu.cn (Z.T.)

**Keywords:** MRSA, anti-biofilms agents, *icaA*, *agrA*, colchicine derivatives

## Abstract

Background/Objectives: The elimination of bacterial biofilm formation is an effective strategy against bacterial infections. The objective was to design 27 colchicine C-ring modified amine derivatives and evaluate their inhibitory activities against the biofilms of MRSA USA300. Methods: Design 27 colchicine C-ring modified amine derivatives. Evaluate their inhibitory activities against MRSA USA300 biofilms. Conduct antibacterial or synergistic antibacterial experiments. Research the phenotypic mechanisms related to biofilm-related genes *icaA* and *agrA*. Results: The experiments showed that most compounds in this series exhibited varying degrees of biofilm inhibitory activity (with inhibition rates ranging from 7.72% to 40.79%). Further verification through antibacterial or synergistic antibacterial experiments revealed that the compounds with biofilm-inhibiting effects (compounds **7b–11b**) generally had certain antibacterial activities (MICs = 16–32 μg/mL) or synergistic antibacterial effects (FICIs < 0.5). Furthermore, through in-depth research on their phenotypic mechanisms (i.e., research on biofilm-related mechanisms), it was found that the compounds with antibacterial or synergistic antibacterial properties could inhibit the formation of biofilms by affecting the regulation of the biofilm-related genes *icaA* and *agrA*. Conclusions: The designed colchicine C-ring modified amine derivatives showed potential in inhibiting MRSA biofilms, and their antibacterial or synergistic antibacterial properties are related to the regulation of biofilm-related genes *icaA* and *agrA*, demonstrating inhibitory activity against MRSA.

## 1. Introduction

Since the 1960s, methicillin-resistant *Staphylococcus aureus* (MRSA) has emerged and spread globally, becoming one of the leading causes of bacterial infections in healthcare and community settings. Despite the continuous development of novel antibiotics and advancements in infection prevention measures, the mortality rate associated with MRSA remains high due to its broad-spectrum resistance and virulence [1]. Therefore, it is critical to develop a new antibacterial drug and research adjunct therapies to overcome its resistance.

Additionally, another key factor contributing to this problem is the biofilm formation by MRSA [2]. Biofilms can occur on both biological and non-biological surfaces, complicating MRSA infections and making clinical treatment more challenging [3], leading to prolonged hospitalization and increased mortality. Thus, eliminating the formation of bacterial biofilms is an effective strategy against bacterial infections. Targeting the regulatory factors and proteins involved in MRSA biofilm formation is one method to control and prevent biofilm-related infections [4,5]. Based on a complex molecular basis, the biofilm formation of MRSA involves various regulatory factors within multiple regulatory systems, including *icaADBC, agr, sarA, sarZ, sigB, sarX*, and *icaR* [6]. Among the factors mentioned above, the more dominant one is the ica operon, on which the synthesis of polysaccharide intercellular adhesin (PIA) depends [7]. The ica operon is composed of the closely linked genes *icaA*, *D, B,* and *C*, along with the upstream regulatory gene *icaR* [8]. Among these, the *icaA* operon plays the most significant role, and the IcaA protein synthesized by its encoding has *N*-acetylglucosyl transferase activity, which is able to regulate PIA synthesis with the assistance of other ica operons in order to promote the formation of the biofilm [9]. Moreover, the agr-mediated quorum sensing system has a critical impact on biofilm formation in *staphylococci*, including MRSA [10,11,12]. This system comprises four main components encoded by the agrBDCA operon [13]. Therein, AgrA, which is encoded by *agrA*, plays a prominent role in biofilm formation, because it is not only fully expressed within biofilms but also participates in different stages of biofilm formation, such as cell adhesion and detachment [14,15].

So far, chemical modifications of naturally derived bioactive compounds remain one of the most effective sources of antibiotics [16]. Moreover, unlike traditional antibiotics, compounds modified from natural sources may exhibit different antibacterial mechanisms, inhibiting bacterial growth through these mechanisms and providing lead molecules for further research [17]. Among these, colchicine, one of the oldest drugs and a well-known natural molecule, has gained extensive attention in fields such as anticancer, analgesic, and anti-inflammatory therapies. It has been clinically found to treat conditions like acute gout, Behçet’s disease, and familial Mediterranean fever, and it has also shown effective activity in preclinical studies for diseases such as cancer, autoimmune, and neurodegenerative diseases [18,19,20,21]. Despite its widespread application in these areas, the pharmacological potential of colchicine as a lead compound has yet to be fully exploited [22].

Currently, the structural optimization and activity exploration of colchicine primarily focus on its binding with tubulin and research in the anticancer field [23]. Interestingly, recent studies have found that some C-ring amine-modified derivatives of colchicine with increased lipid solubility and extended modified side chains enable them to inhibit the growth and reproduction of MRSA in a lower concentration range, although their specific mechanisms of action remain unknown [24,25]. Meanwhile, many other studies have shown that when the methoxy group at the C-10 position of the C-ring is replaced with an amino or sulfhydryl group, these compounds not only alter biological properties but also enhance molecular stability. Furthermore, compared to the deacetylation modifications of the B-ring side chain, modifying the methoxy group on the C-ring is simpler and does not produce any undesirable changes in the structure of these analogs [26,27,28,29,30,31]. These findings suggest that perhaps colchicine C-ring amine-modified derivatives could be a very promising lead compound in the inhibition of MRSA.

Therefore, the current study primarily focuses on two main objectives. First, it aims to modify the methoxy group on the C-ring of colchicine using amine substitution. Second, it conducts a preliminary exploration of the structure-activity relationship and an in-depth investigation into a specific phenotypic mechanism in the context of antimicrobial research, namely the antibiofilm activity. The ultimate intention is to offer novel insights for the treatment of MRSA infections.

## 2. Results and Discussion

### 2.1. Chemistry

To investigate the properties of the side chain at C-10 of colchicine and the influence of lipophilicity on biological activity, 27 C-ring-modified amine derivatives were synthesized. For a better structure-activity relationship (SAR) analysis, we designed amine side chains with varying structures, including different lengths of saturated alkyl chains (**6b–11b**, **14b**, and **16b**), alkyl chains containing oxygen, nitrogen, or sulfur atoms (**1b–5b**, **12b–13b**, and **15b**), alkyl chains with aromatic substituents (**17b–21b**, **24–26b**), alkyl chains with cyclic structures (**22b–23b**), and side chains containing effective structural fragments of marketed drugs (**27b**).

**Scheme 1:**
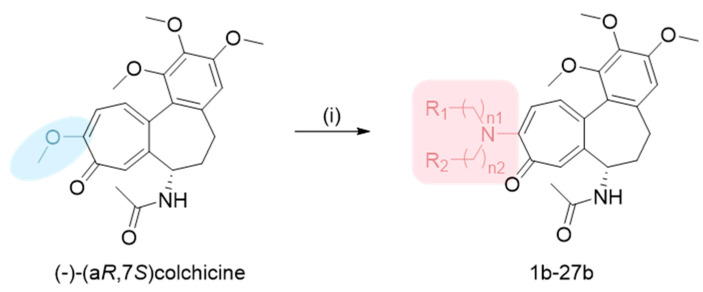
Reagents and conditions: (i) 5–15 equiv amines, ethanol, reflux, 1–36 h. Time for completion of the reaction at reflux as indicated by TLC.

The synthetic route for colchicine derivatives **1b–27b** is shown in Scheme 1. All designed compounds were synthesized starting from colchicine with various amine fragments in ethanol, refluxed for 1–36 h (60–88% yield).

Previous studies have successfully identified that under these conditions, the methoxy group at C (10) of the colchicine’s tropolone ring undergoes regioselective one-pot reactions with the corresponding amines using spectroscopic methods [24,31]. The structures of all target products were confirmed by HRMS, ^1^H NMR, and ^13^C NMR, with details provided in the Supplementary Materials (the structures of the target compounds are shown in Table 1).

### 2.2. Biological Evaluation of **1b–27b**

#### 2.2.1. Biofilm Inhibition ASSAY

**Figure 1:**
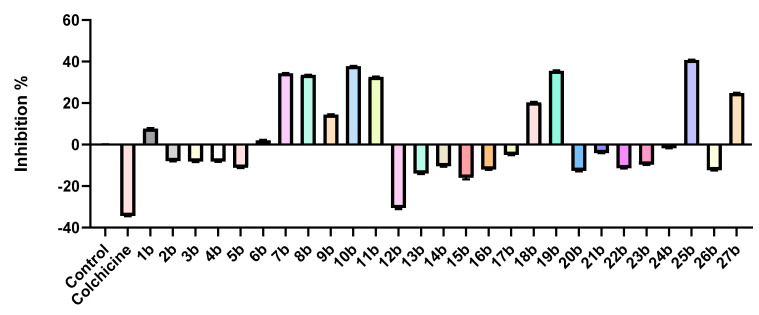
Percent of biofilm inhibition. Determined after colchicine derivatives **1b−27b** and colchicine.

Using colchicine derivatives **1b–27b** at 1/2 MIC concentration to treat MRSA USA300, we investigated their inhibitory effects on biofilm formation to screen for biofilm inhibitors. As shown in Figure 1, compounds **1b, 6b, 7b, 8b, 9b, 10b, 11b, 18b, 19b, 25b**, and **27b** exhibited inhibitory effects on the biofilm formation of MRSA, with inhibition rates of 7.72%, 2.06%, 34.40%, 33.54%, 14.34%, 37.79%, 32.66%, 20.33%, 35.54%, 40.79%, and 24.76%, respectively.

#### 2.2.2. Determination of MICs and FICIs

The MIC and FICI values for this series of derivatives are summarized in Table 2. We unexpectedly found that compounds **14b** (MIC = 32–64 μg/mL) and **15b** (MIC = 64–256 μg/mL), previously reported to have inhibitory activity against MRSA [24], did not exhibit anti-MRSA USA300 activity in this study, which may be due to the difference in the selected strains that led to the different results of activity. Among the previously tested compounds with biofilm inhibition, compound 9b showed the expected inhibition of MRSA USA300 with a MIC of 16 μg/mL. Moreover, compounds **10b** and **11b** could also effectively inhibit the growth of MRSA USA300 at a concentration of 32 μg/mL. Unfortunately, the remaining biofilm-inhibiting compounds did not effectively inhibit MRSA USA300 (particularly compound **25b**, which had the highest biofilm inhibition rate of 40.79% in this series of colchicine derivatives), and their MICs were more than 1024 μg/mL.

Given the increasing resistance of MRSA USA300 to β-lactam antibiotics, we chose penicillin G and cefoxitin, two typical β-lactam antibiotics, for combination testing with compounds **1b–27b** to determine the fractional inhibitory concentration index (FICI). The results revealed that biofilm-inhibiting compounds **7b, 8b, 10b**, and **11b** exhibited a degree of synergistic antibacterial activity when used in combination with penicillin G (FICI < 0.5), as expected. More surprisingly, compounds **14b** and **16b** were able to produce synergistic antimicrobial effects in the presence of penicillin G, wherein compound **16b** was also able to produce synergistic antimicrobial effects in combination with cefoxitin. Based on the above experimental results, it can be inferred that compounds **7b–11b** can have a certain degree of antibacterial effect or synergistic activity, possibly due to their biofilm-inhibiting properties. This brings some opportunities for the subsequent in-depth study of the mechanism.

### 2.3. Activity of **7b−11b** on Biofilm Formation

#### 2.3.1. Effects of **7b−11b** on the Development of MRSA USA300 Biofilm

**Figure 2:**
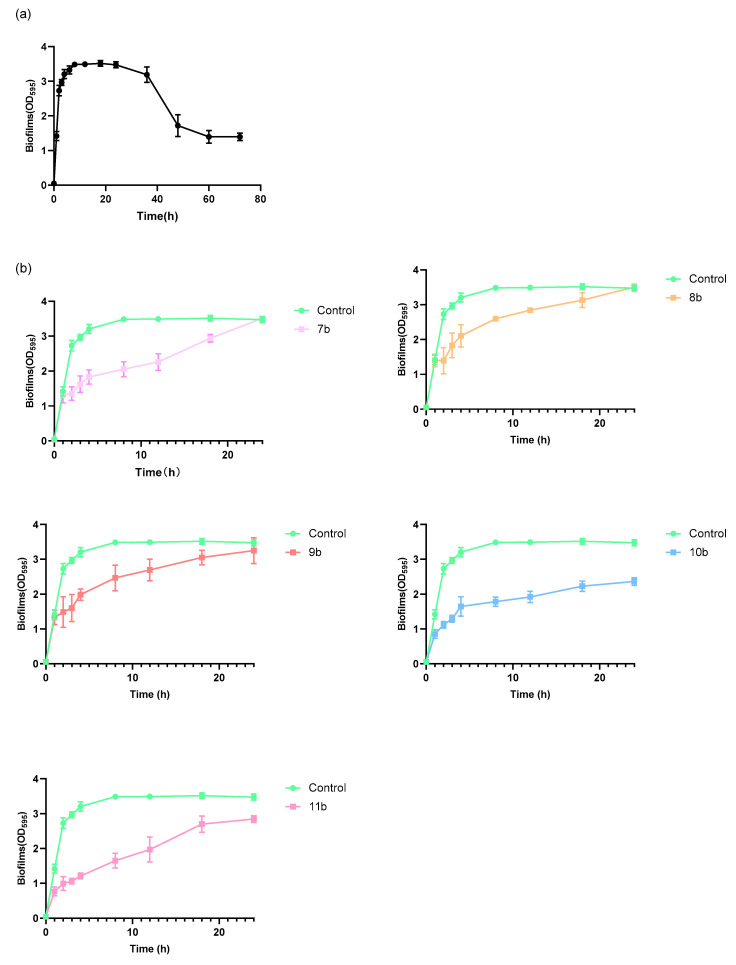
(**a**) The formation process of MRSA biofilm. (**b**) The formation process of MRSA biofilm under treatment with compounds **7b−11b**.

This experiment assessed the biofilm development of MRSA USA300 in 3% sucrose BHI broth using the crystal violet staining method. As shown in Figure 2a, during the 0–1 h period, free bacteria attached to the 96-well plates, but biofilm formation had not yet occurred. From 1–12 h, the adherent bacteria continued to grow, gradually forming small colonies while continuously secreting self-synthesized extracellular polymers, which facilitated biofilm formation. The OD_595_ values stabilized around 3.5 from 12 h to 36 h, indicating that the biofilm had matured and was no longer growing. After 36 h, the amount of biofilm began to decrease, with the OD_595_ values continuously declining, signaling the onset of biofilm diffusion. From this, it can be concluded that the process of biofilm formation of MRSA USA300 under this culture condition is as follows: 0–1 h is the stage of bacterial adhesion, 1–12 h is the stage of bacterial proliferation, 12–36 h is the stage of biofilm maturation, and 36 h is the stage of biofilm diffusion.

To investigate the mechanism of action of compounds **7b–11b** in inhibiting the formation of a MRSA USA300 biofilm, we added each compound at 1/2 MIC concentration during different stages of biofilm development and halted the culture after 24 h. Then we measured the amount of crystal violet attached to the biofilm using a microplate reader to observe the effects of compounds **7b–11b** on biofilm development. As shown in Figure 2b, the results indicate that adding compound **7b** at 2 h inhibited 50.38% of MRSA biofilm formation, but this inhibition decreased to approximately 47.46% when added at 12 h, and nearly 0% at 24 h. Similarly, adding compound 8b at 2 h resulted in a 48.96% inhibition rate, which dropped to about 18.51% at 12 h and nearly 0% at 24 h. For compound **9b**, a 45.58% inhibition was observed at 2 h, decreasing to approximately 22.95% at 12 h, and nearly 0% at 24 h. Adding compound **10b** at 2 h led to a 59.06% inhibition rate, which decreased to about 45.04% at 12 h and decreased to 32.09% at 24 h. Compound **11b** exhibited a 63.74% inhibition at 2 h, declining to approximately 43.66% at 12 h, and to 18.20% at 24 h. These findings indicate that compounds **7b–11b** significantly inhibit MRSA biofilm formation during the initial 0–2 h period. Adding compounds **7b–9b** after 24 h had almost no effect on biofilm development compared to the control group, while adding compounds **10–11b** after 24 h reduced their inhibitory effects on biofilm formation. This suggests that compounds **7b–11b** primarily inhibit the bacterial proliferation and maturation phases of MRSA USA300 biofilm development.

#### 2.3.2. Compounds **7b−11b** Alter the Expression of *S. aureus* Biofilm Genes

**Figure 3:**
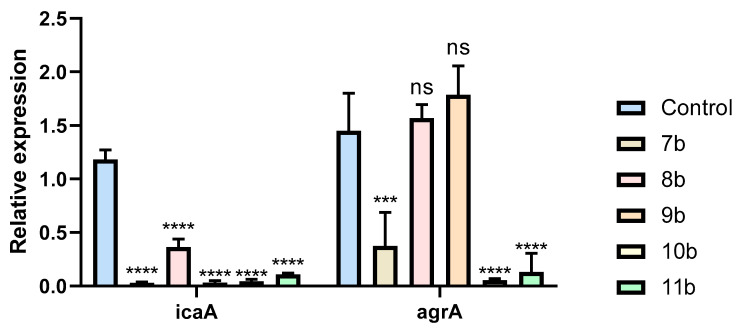
Transcriptional profile of MRSA USA300 biofilm genes upon **7b−11b** treatment. **** Significantly different from control group (**** *p* < 0.0001). *** Significantly different from control group (*** *p* = 0.005). Not significantly different from control group (ns).

To investigate the effects of compounds **7b–11b** on biofilm-related genes, qPCR was used to measure changes in the expression of the MRSA biofilm-related genes *icaA* and *agrA* after treatment with these compounds. As shown in Figure 3, all five compounds significantly reduced the transcription levels of the biofilm-related gene *icaA* in MRSA. However, for the *agrA* gene, only compounds **7b, 10b**, and **11b** exhibited a significant reduction in transcription, while compounds **8b** and **9b** had no effect. These results suggest that colchicine derivatives **7b−11b** can interfere with MRSA biofilm formation by regulating the expression of the biofilm-related genes *icaA* and *agrA*, thereby contributing to their antibacterial effects or potential synergistic antibacterial activity.

### 2.4. Effect of **7b−11b** on Biofilm Structure

**Figure 4:**
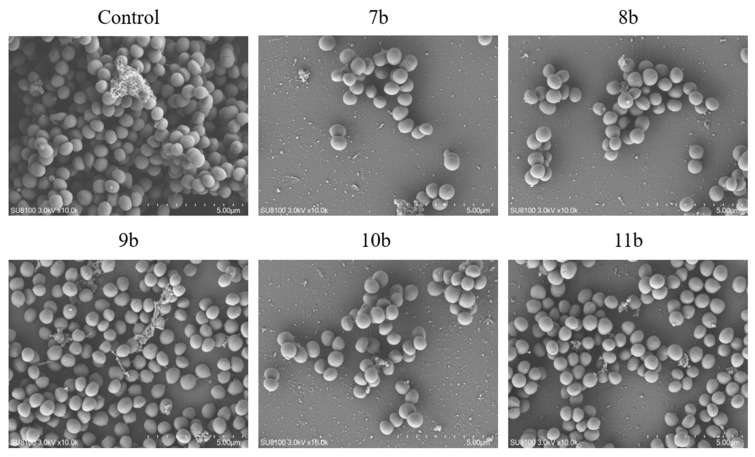
SEM photos of MRSA biofilm treated with different concentrations of **7b−11b**.

To visually demonstrate the overall structural changes in the biofilm, SEM imaging was used to observe and document images of the biofilm. Figure 4 shows notable differences in the MRSA biofilm between the **7b–11b**-treated group and the control group. The control group displayed large clusters and colonies of bacteria. By contrast, the biofilm treated with **7b–11b** at or above the 1/2 MIC showed a more relaxed overall structure with a significant reduction in bacterial adhesion and aggregation. These results likely reflect suppression of the production of extracellular polysaccharides, such as PIA, leading to reduced bacterial adhesion and aggregation capabilities and hence reduced resistance to antibacterial agents.

### 2.5. ADME Prediction

**Figure 5:**
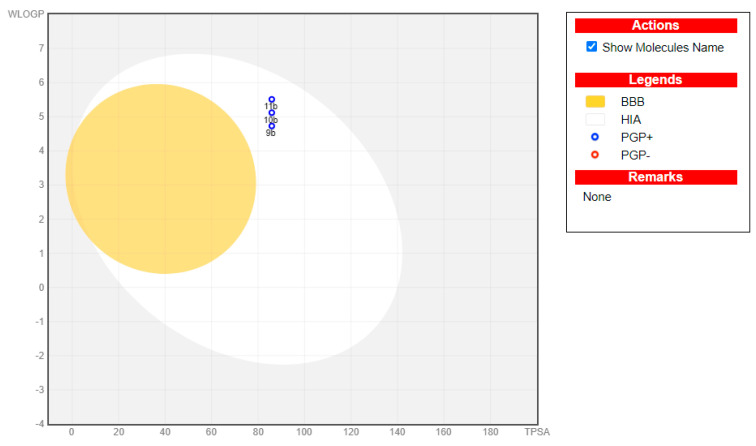
BOILED-Egg plot of colchicine derivatives (**9b−11b**).

Undesirable pharmacokinetic properties are a major reason for drug development failures, so it is essential to study the pharmacokinetics of compounds during drug development. In this study, the SwissADME online tool (http://www.swissadme.ch, accessed on 8 October 2024) was used to predict the ADME properties of the most active compounds in the series (**9b−11b**). As shown in Figure 5, the BOILED-Egg plot illustrates the WLOGP versus TPSA of the tested compounds. Table 3 provides additional predictive data. Notably, none of the compounds exhibited blood-brain barrier (BBB) permeability, indicating central nervous system (CNS) safety. All compounds were identified as substrates of P-glycoprotein (PGP+), although this aspect has a minimal impact on their activity against MRSA, which does not directly express P-glycoprotein. Additionally, the compounds demonstrated high gastrointestinal absorption (oral bioavailability). However, as indicated in Table 3, the compounds showed poor solubility (which may account for some inactive compounds) and low skin permeability (the more negative the log Kp, the less skin permeant is the molecule). In general, all tested compounds complied with Lipinski’s rule. However, the lipophilicity of compounds **9–11b** increased with the number of carbon atoms, potentially enhancing their ability to penetrate cell membranes and thus increasing toxicity.

## 3. Materials and Methods

### 3.1. Chemistry

#### 3.1.1. General

All of the solvents and chemical materials utilized herein were obtained from Energy Chemical (Anhui, China) or Bidepharm (Shanghai, China) and were used without further purification. All of the reagents were treated with activated 4Å molecular sieves. TLC (Thin Layer Chromatography) analysis was performed using precoated plates (0.2–0.25 mm thickness, TLC silica gel 60 F_254_) from Qingdao Hai Yang Chemical (Qingdao, China), and spots were detected by UV-light. The ^1^H-NMR and ^13^C-NMR spectra were recorded on a Varian spectrometer (Thermo Scientific, Bremen, Germany) (^1^H-NMR at 300 MHz and ^13^C-NMR at 75 MHz), using CDCl_3_-*d*, DMSO-*d*_6_, CD_3_OD-*d*_4_ as solvents. ^1^H-NMR spectra are reported as follows: chemical shift (δ, ppm), multiplicity (s = singlet, d = doublet, t = triplet, m = multiplet), coupling constant (*J*) in Hz, and integration. ^13^C-NMR spectra are reported in chemical shifts downward from TMS using the residual solvent peak as internal standard. HRMS (Shimadzu, Japan) were obtained on Orbitrap Exploris 480 mass Spectrometry (Thermo Scientific, Bremen, Germany).

#### 3.1.2. General Procedure for the Synthesis of Colchicine Derivatives (**1b-27b**)

To a solution of colchicine (a) (1.0 equiv.) in EtOH, amine (10.0 equiv.) in the designed program was added. The mixture was stirred at reflux for 1–36 h. At the end of the reaction, the mixture was distilled under reduced pressure. Then the residue was dissolved in CH_2_Cl_2_ and purified by column chromatography (silica gel: DCM/MeOH) and next recrystallized (PE/DCM) to give the product as a yellow solid with a yield of 60–88%.

(*S*)-*N*-(10-((3-hydroxypropyl)amino)-1,2,3-trimethoxy-9-oxo-5,6,7,9-tetrahydrobenzo[*a*]heptalen-7-yl)acetamide (**1b**)

Yellow solid (yield 73%); ^1^H NMR (300 MHz, Chloroform-*d*) δ 7.68 (t, *J* = 5.0 Hz, 1H), 7.59 (d, *J* = 7.2 Hz, 1H), 7.50–7.40 (m, 2H), 6.67 (d, *J* = 11.4 Hz, 1H), 6.52 (s, 1H), 4.73–4.62 (m, 1H), 3.93 (s, 3H), 3.88 (s, 3H), 3.81 (t, *J* = 5.7 Hz, 2H), 3.61 (s, 3H), 3.59–3.48 (m, 2H), 2.60 (s, 2H), 2.50–2.30 (m, 2H), 2.29–2.19 (m, 1H), 2.00 (s, 3H), 1.97–1.91 (m, 1H); ^13^C NMR (75 MHz, cdcl_3_) δ 174.47, 169.84, 154.30, 152.58, 150.88, 150.66, 141.15, 139.60, 134.25, 130.14, 126.45, 122.73, 109.04, 106.94, 60.99, 59.87, 56.68, 52.85, 39.47, 37.34, 30.88, 29.74, 22.48. C_24_H_30_N_2_O_6_ (442.2104), HRMS: m/z 443.2182 [M + H]^+^.

(*S*)-*N*-(1,2,3-trimethoxy-10-((2-methoxyethyl)amino)-9-oxo-5,6,7,9-tetrahydrobenzo[*a*]heptalen-7-yl)acetamide (**2b**)

Yellow solid (yield 77%); ^1^H NMR (300 MHz, Chloroform-*d*) δ 7.73 (d, *J* = 7.2 Hz, 1H), 7.55 (s, 1H), 7.45 (d, *J* = 11.2 Hz, 2H), 6.66 (d, *J* = 11.4 Hz, 1H), 6.53 (s, 1H), 4.76–4.62 (m, 1H), 3.94 (s, 3H), 3.89 (s, 3H), 3.72–3.68 (m, 2H), 3.62 (s, 3H), 3.60–3.50 (m, 2H), 3.42 (s, 3H), 2.50–2.41 (m, 1H), 2.40–2.33 (m, 1H), 2.31 (s, 2H), 1.99 (s, 3H). ^13^C NMR (75 MHz, cdcl_3_) δ 174.63, 169.74, 154.01, 152.60, 151.08, 150.76, 141.22, 138.87, 134.28, 130.45, 126.54, 122.85, 108.30, 106.96, 69.76, 61.06, 58.68, 55.84, 52.30, 42.18, 36.81, 29.78, 22.49. C_24_H_30_N_2_O_6_ (442.2104), HRMS: m/z 443.2178 [M + H]^+^.

(*S*)-*N*-(1,2,3-trimethoxy-10-((3-methoxypropyl)amino)-9-oxo-5,6,7,9-tetrahydrobenzo[*a*]heptalen-7-yl)acetamide (**3b**)

Yellow solid (yield 75%); ^1^H NMR (300 MHz, Chloroform-*d*) δ 7.87 (d, *J* = 7.2 Hz, 1H), 7.53–7.38 (m, 3H), 6.63 (d, *J* = 11.3 Hz, 1H), 6.52 (s, 1H), 4.77–4.62 (m, 1H), 3.94 (s, 3H), 3.89 (s, 3H), 3.62 (s, 3H), 3.57–3.43 (m, 4H), 3.37 (s, 3H), 2.51–2.42 (m, 1H), 2.41–2.20 (m, 2H), 2.06–1.99 (m, 2H), 1.98 (s, 3H), 1.95–1.91 (m, 1H). ^13^C NMR (75 MHz, cdcl_3_) δ 174.68, 169.71, 154.17, 152.51, 151.01, 150.78, 141.19, 138.91, 133.54, 129.95, 126.64, 122.47, 108.02, 106.84, 69.93, 62.16, 61.48, 59.00, 56.31, 52.29, 40.05, 36.74, 29.80, 28.31, 21.94. C_25_H_32_N_2_O_6_ (456.2260), HRMS: m/z 457.2339 [M + H]^+^.

(*S*)-*N*-(10-((2-(dimethylamino)ethyl)amino)-1,2,3-trimethoxy-9-oxo-5,6,7,9-tetrahydrobenzo[*a*]heptalen-7-yl)acetamide (**4b**)

Yellow solid (yield 65%); ^1^H NMR (300 MHz, Chloroform-*d*) δ 7.58 (d, *J* = 7.2 Hz, 1H), 7.50 (t, 1H), 7.46 (s, 1H), 7.41 (d, *J* = 11.1 Hz, 1H), 6.57 (d, *J* = 11.3 Hz, 1H), 6.52 (s, 1H), 4.74–4.64 (m, 1H), 3.94 (s, 3H), 3.89 (s, 3H), 3.62 (s, 3H), 3.46–3.37 (m, 2H), 2.68 (t, *J* = 6.4 Hz, 2H), 2.49–2.35 (m, 2H), 2.31 (s, 6H), 2.29–2.23 (m, 1H), 1.98 (s, 3H), 1.95–1.86 (m, 1H). ^13^C NMR (75 MHz, cdcl_3_) δ 174.97, 169.64, 156.85, 153.96, 152.50, 150.79, 141.22, 138.69, 133.79, 129.84, 126.67, 122.45, 108.03, 106.91, 61.05, 56.77, 55.80, 51.89, 44.99, 40.09, 38.87, 36.83, 29.82, 22.53. C_25_H_33_N_3_O_5_ (455.2420), HRMS: m/z 456.2498 [M + H]^+^.

(*S*)-*N*-(10-((2,2-dimethoxyethyl)amino)-1,2,3-trimethoxy-9-oxo-5,6,7,9-tetrahydrobenzo[*a*]heptalen-7-yl)acetamide (**5b**)

Yellow solid (yield 60%); ^1^H NMR (300 MHz, Chloroform-*d*) δ 7.77 (d, 1H), 7.53 (s, 1H), 7.43 (d, *J* = 11.0 Hz, 1H), 7.33 (s, 1H), 6.65 (d, *J* = 11.2 Hz, 1H), 6.53 (s, 1H), 4.76–4.61 (m, 2H), 3.91 (d, *J* = 13.8 Hz, 6H), 3.62 (s, 3H), 3.53–3.48 (m, 2H), 3.44 (s, 6H), 2.54–2.33 (m, 2H), 2.33–2.17 (m, 2H), 1.98 (s, 3H). ^13^C NMR (75 MHz, cdcl_3_) δ 174.81, 169.67, 153.79, 152.61, 151.16, 150.78, 141.23, 138.73, 134.24, 130.22, 126.51, 123.13, 108.30, 107.38, 101.54, 61.58, 60.63, 55.81, 53.46, 52.29, 44.12, 36.75, 29.79, 22.50. C_25_H_32_N_2_O_7_ (472.2210), HRMS: m/z 473.2289 [M + H]^+^.

(*S*)-*N*-(10-(butylamino)-1,2,3-trimethoxy-9-oxo-5,6,7,9-tetrahydrobenzo[*a*]heptalen-7-yl)acetamide (**6b**)

Yellow crystal (yield 88%); ^1^H NMR (300 MHz, Chloroform-*d*) δ 7.74 (d, *J* = 7.1 Hz, 1H), 7.49 (s, 1H), 7.43 (d, *J* = 11.2 Hz, 1H), 7.23 (d, *J* = 6.0 Hz, 1H), 6.60 (d, *J* = 11.3 Hz, 1H), 6.53 (s, 1H), 4.76–4.62 (m, 1H), 3.94 (s, 3H), 3.89 (s, 3H), 3.62 (s, 3H), 3.42–3.30 (m, 2H), 2.51–2.35 (m, 2H), 2.33–2.17 (m, 1H), 1.99 (s, 3H), 1.97–1.87 (m, 1H), 1.82–1.70 (m, 2H), 1.54–1.44 (m, 2H), 0.99 (t, *J* = 7.3 Hz, 3H). ^13^C NMR (75 MHz, cdcl_3_) δ 174.52, 169.72, 154.03, 152.53, 151.00, 150.77, 141.20, 138.98, 134.29, 129.93, 126.65, 122.32, 108.15, 106.85, 62.87, 61.05, 55.80, 52.34, 42.21, 36.77, 30.25, 29.40, 22.45, 19.99, 13.44. C_25_H_32_N_2_O_5_ (440.2311), HRMS: m/z 441.2387 [M + H]^+^.

(*S*)-*N*-(1,2,3-trimethoxy-9-oxo-10-(pentylamino)-5,6,7,9-tetrahydrobenzo[*a*]heptalen-7-yl)acetamide (**7b**)

Yellow crystal (yield 85%); ^1^H NMR (300 MHz, Chloroform-*d*) δ 7.96 (d, *J* = 7.0 Hz, 1H), 7.51 (s, 1H), 7.43 (d, *J* = 11.2 Hz, 1H), 7.24 (s, 1H), 6.60 (d, *J* = 11.3 Hz, 1H), 6.52 (s, 1H), 4.74–4.64 (m, 1H), 3.91 (d, *J* = 14.8 Hz, 6H), 3.62 (s, 3H), 3.41–3.29 (m, 2H), 2.51–2.41 (m, 1H), 2.39–2.18 (m, 2H), 2.17–2.02 (m, 1H), 1.98 (s, 3H), 1.80–1.72 (m, 2H), 1.45–1.39 (m, 4H), 0.99–0.89 (m, 3H). ^13^C NMR (75 MHz, cdcl_3_) δ 174.55, 169.72, 154.02, 152.51, 151.02, 150.77, 141.20, 138.96, 134.28, 129.91, 126.65, 122.32, 108.12, 106.86, 61.69, 61.03, 55.78, 52.33, 42.47, 36.74, 29.79, 28.94, 27.90, 22.44, 22.02, 13.63. C_26_H_34_N_2_O_5_ (454.2468), HRMS: m/z 455.2541 [M + H]^+^.

(*S*)-*N*-(10-(hexylamino)-1,2,3-trimethoxy-9-oxo-5,6,7,9-tetrahydrobenzo[*a*]heptalen-7-yl)acetamide (**8b**)

Yellow crystal (yield 80%); ^1^H NMR (300 MHz, Chloroform-*d*) δ 7.73 (s, 1H), 7.50–7.38 (m, 2H), 7.24 (d, 1H), 6.59 (d, *J* = 11.3 Hz, 1H), 6.52 (s, 1H), 4.76–4.62 (m, 1H), 3.94 (s, 3H), 3.89 (s, 3H), 3.62 (s, 3H), 3.41–3.29 (m, 2H), 2.51–2.34 (m, 2H), 2.30–2.19 (m, 1H), 1.98 (s, 3H), 1.96–1.86 (m, 1H), 1.82–1.72 (m, 2H), 1.49–1.41 (m, 2H), 1.38–1.30 (m, 4H), 0.94–0.89 (m, 3H). ^13^C NMR (75 MHz, cdcl_3_) δ 175.67, 170.68, 155.06, 153.53, 151.84, 151.79, 142.23, 139.95, 135.27, 130.82, 127.67, 123.35, 109.10, 107.85, 62.08, 61.07, 56.83, 53.31, 43.52, 37.87, 32.13, 30.81, 29.19, 27.50, 23.52, 23.23, 15.28. C_27_H_36_N_2_O_5_ (468.2624), HRMS: m/z 469.2698 [M + H]^+^.

(*S*)-*N*-(10-(heptylamino)-1,2,3-trimethoxy-9-oxo-5,6,7,9-tetrahydrobenzo[*a*]heptalen-7-yl)acetamide (**9b**)

Yellow solid (yield 79%); ^1^H NMR (300 MHz, Chloroform-*d*) δ 7.68 (d, *J* = 7.1 Hz, 1H), 7.52–7.40 (m, 2H), 7.24 (d, *J* = 5.8 Hz, 1H), 6.60 (d, *J* = 11.3 Hz, 1H), 6.52 (s, 1H), 4.77–4.62 (m, 1H), 3.94 (s, 3H), 3.89 (s, 3H), 3.62 (s, 3H), 3.42–3.29 (m, 2H), 2.51–2.36 (m, 2H), 2.32–2.19 (m, 1H), 1.99 (s, 3H), 1.97–1.87 (m, 1H), 1.85–1.69 (m, 2H), 1.48–1.28 (m, 8H), 0.95–0.84 (m, 3H). ^13^C NMR (75 MHz, cdcl_3_) δ 174.90, 170.02, 154.34, 152.83, 151.25, 151.08, 141.51, 139.26, 134.59, 130.17, 126.96, 122.63, 108.41, 107.16, 61.36, 60.12, 56.09, 52.64, 42.81, 37.09, 31.68, 30.11, 28.92, 28.52, 27.48, 22.77, 22.55, 14.05. C_28_H_38_N_2_O_5_ (482.2781), HRMS: m/z 483.2855 [M + H]^+^.

(*S*)-*N*-(1,2,3-trimethoxy-10-(octylamino)-9-oxo-5,6,7,9-tetrahydrobenzo[*a*]heptalen-7-yl)acetamide (**10b**)

Yellow solid (yield 75%); ^1^H NMR (300 MHz, Chloroform-*d*) δ 7.71 (d, *J* = 7.2 Hz, 1H), 7.54 (s, 1H), 7.46 (d, *J* = 11.2 Hz, 1H), 6.63 (d, *J* = 11.4 Hz, 1H), 6.53 (s, 1H), 4.77–4.64 (m, 1H), 3.94 (s, 3H), 3.89 (s, 3H), 3.62 (s, 3H), 3.42–3.29 (m, 2H), 2.50–2.43 (m, 1H), 2.39–2.19 (m, 2H), 2.00 (s, 3H), 2.00–1.89 (m, 1H), 1.82–1.71 (m, 2H), 1.39–1.23 (m, 10H), 0.93–0.83 (m, 3H). ^13^C NMR (75 MHz, cdcl_3_) δ 173.85, 169.80, 154.02, 152.58, 151.27, 150.72, 141.17, 139.27, 134.32, 130.36, 126.52, 122.31, 108.61, 106.89, 61.04, 55.79, 55.75, 52.33, 42.53, 36.78, 31.40, 29.79, 28.88, 28.82, 28.18, 26.79, 22.44, 22.27, 13.73. C_29_H_40_N_2_O_5_ (496.2937), HRMS: m/z 497.3007 [M + H]^+^.

(*S*)-*N*-(1,2,3-trimethoxy-10-(nonylamino)-9-oxo-5,6,7,9-tetrahydrobenzo[*a*]heptalen-7-yl)acetamide (**11b**)

Yellow solid (yield 77%); ^1^H NMR (300 MHz, Chloroform-*d*) δ 7.91 (d, *J* = 7.2 Hz, 1H), 7.49 (s, 1H), 7.43 (d, *J* = 11.2 Hz, 1H), 7.22 (d, *J* = 6.9 Hz, 1H), 6.59 (d, *J* = 11.3 Hz, 1H), 6.52 (s, 1H), 4.75–4.63 (m, 1H), 3.94 (s, 3H), 3.89 (s, 3H), 3.62 (s, 3H), 3.39–3.28 (m, 2H), 2.52–2.32 (m, 2H), 2.31–2.19 (m, 1H), 1.98 (s, 3H), 1.94 (s, 1H), 1.80–1.71 (m, 2H), 1.39–1.24 (m, 12H), 0.91–0.82 (m, 3H). ^13^C NMR (75 MHz, cdcl_3_) δ 174.51, 169.71, 154.06, 152.56, 150.95, 150.78, 141.24, 139.01, 134.28, 129.95, 126.65, 122.35, 108.21, 106.90, 61.05, 55.81, 52.31, 42.53, 36.86, 31.52, 29.82, 29.15, 28.97, 28.90, 28.24, 26.83, 22.50, 22.32, 13.77. C_30_H_42_N_2_O_5_ (510.3094), HRMS: m/z 511.3164 [M + H]^+^.

(*S*)-*N*-(1,2,3-trimethoxy-10-((2-(methylthio)ethyl)amino)-9-oxo-5,6,7,9-tetrahydrobenzo[*a*]heptalen-7-yl)acetamide (**12b**)

Yellow crystal (yield 80%); ^1^H NMR (300 MHz, Chloroform-*d*) δ 7.77 (d, *J* = 7.2 Hz, 1H), 7.50 (s, 1H), 7.48–7.38 (m, 2H), 6.64–6.52 (m, 2H), 4.77–4.62 (m, 1H), 3.94 (s, 3H), 3.89 (s, 3H), 3.65–3.54 (m, 5H), 2.86 (t, *J* = 6.9 Hz, 2H), 2.52–2.33 (m, 2H), 2.29–2.22 (m, 1H), 2.18 (s, 3H), 1.98 (s, 3H), 1.96–1.88 (m, 1H). ^13^C NMR (75 MHz, cdcl_3_) δ 175.45, 170.21, 154.07, 153.16, 151.79, 151.29, 141.78, 139.29, 134.77, 131.13, 127.03, 123.60, 108.66, 107.47, 61.59, 56.37, 52.83, 41.85, 37.31, 32.82, 30.32, 23.03, 15.73, 1.21. C_24_H_30_N_2_O_5_S (458.1875), HRMS: m/z 459.1953 [M + H]^+^.

(*S*)-*N*-(1,2,3-trimethoxy-10-((3-(methylthio)propyl)amino)-9-oxo-5,6,7,9-tetrahydrobenzo[*a*]heptalen-7-yl)acetamide (**13b**)

Yellow crystal (yield 75%); ^1^H NMR (300 MHz, Chloroform-*d*) δ 7.71 (d, *J* = 7.2 Hz, 1H), 7.50–7.39 (m, 2H), 6.65 (d, *J* = 11.3 Hz, 1H), 6.52 (s, 1H), 4.76–4.63 (m, 1H), 3.93 (s, 3H), 3.89 (s, 3H), 3.62 (s, 3H), 3.56–3.47 (m, 2H), 2.64 (t, *J* = 6.9 Hz, 2H), 2.52–2.32 (m, 2H), 2.31–2.18 (m, 1H), 2.13 (s, 3H), 2.05 (t, 2H), 1.98 (s, 3H), 1.95–1.87 (m, 1H). ^13^C NMR (75 MHz, cdcl_3_) δ 175.06, 169.94, 154.21, 152.90, 151.35, 151.07, 141.55, 139.20, 134.52, 130.58, 126.85, 122.94, 108.46, 107.21, 61.34, 61.29, 56.11, 52.60, 41.39, 37.16, 31.62, 30.08, 27.64, 22.80, 15.57. C_25_H_32_N_2_O_5_S (472.2032), HRMS: m/z 473.2102 [M + H]^+^.

(*S*)-*N*-(10-(dibutylamino)-1,2,3-trimethoxy-9-oxo-5,6,7,9-tetrahydrobenzo[*a*]heptalen-7-yl)acetamide (**14b**)

Yellow solid (yield 67%); ^1^H NMR (300 MHz, Methanol-*d*_4_) δ 7.24 (d, *J* = 11.5 Hz, 1H), 6.96 (s, 1H), 6.78–6.69 (m, 2H), 4.52–4.40 (m, 1H), 3.87 (d, *J* = 3.7 Hz, 6H), 3.72–3.57 (m, 4H), 3.56 (s, 3H), 2.64–2.51 (m, 1H), 2.46–2.29 (m, 1H), 2.27–2.08 (m, 1H), 1.98 (s, 3H), 1.97–1.86 (m, 1H), 1.76–1.54 (m, 4H), 1.46–1.26 (m, 4H), 1.05–0.87 (m, 6H). ^13^C NMR (75 MHz, cd_3_od) δ 179.48, 172.16, 157.01, 154.07, 151.95, 150.39, 142.23, 137.73, 136.00, 130.66, 127.33, 124.74, 113.71, 108.40, 61.35, 60.70, 56.31, 53.12, 52.70, 37.78, 30.61, 30.28, 22.22, 20.97, 13.99. C_29_H_40_N_2_O_5_ (496.2937), HRMS: m/z 497.3018 [M + H]^+^.

(*S*)-*N*-(10-(bis(2-methoxyethyl)amino)-1,2,3-trimethoxy-9-oxo-5,6,7,9-tetrahydrobenzo[*a*]heptalen-7-yl)acetamide (**15b**)

Yellow solid (yield 69%); ^1^H NMR (300 MHz, Chloroform-*d*) δ 7.25–7.15 (m, 2H), 6.74 (d, *J* = 11.5 Hz, 1H), 6.49 (s, 1H), 4.66–4.54 (m, 1H), 3.92 (s, 3H), 3.87 (s, 3H), 3.86–3.64 (m, 8H), 3.63 (s, 3H), 3.35 (s, 6H), 2.46–2.36 (m, 2H), 2.32–2.10 (m, 1H), 2.01 (s, 3H), 1.91–1.71 (m, 1H). ^13^C NMR (75 MHz, cdcl_3_) δ 179.27, 169.61, 155.84, 152.54, 151.03, 149.37, 141.24, 136.04, 134.17, 130.60, 125.97, 125.75, 112.94, 106.99, 70.63, 61.02, 60.97, 58.65, 55.79, 52.36, 51.40, 36.74, 29.83, 22.52. C_27_H_36_N_2_O_7_ (500.2523), HRMS: m/z 501.2602 [M + H]^+^.

(*S*)-*N*-(10-(dipentylamino)-1,2,3-trimethoxy-9-oxo-5,6,7,9-tetrahydrobenzo[*a*]heptalen-7-yl)acetamide (**16b**)

Yellow solid (yield 61%); ^1^H NMR (300 MHz, Chloroform-*d*) δ 7.20 (d, *J* = 11.4 Hz, 1H), 7.07 (s, 1H), 6.57–6.47 (m, 2H), 4.69–4.53 (m, 1H), 3.92 (s, 3H), 3.87 (s, 3H), 3.62 (s, 3H), 2.48–2.35 (m, 2H), 2.27–2.08 (m, 1H), 2.01 (s, 3H), 1.88–1.80 (m, 1H), 1.75–1.51 (m, 4H), 1.41–1.25 (m, 8H), 0.99–0.86 (m, 10H). ^13^C NMR (75 MHz, cdcl_3_) δ 178.79, 169.64, 155.62, 152.40, 151.02, 149.04, 141.20, 136.10, 134.31, 129.12, 126.19, 124.56, 111.88, 106.92, 62.23, 60.31, 56.27, 52.81, 51.36, 37.39, 30.33, 28.99, 27.24, 22.61, 22.20, 13.75. C_31_H_44_N_2_O_5_ (524.3250), HRMS: m/z 525.3328 [M + H]^+^.

(*S*)-*N*-(1,2,3-trimethoxy-9-oxo-10-((4-sulfamoylphenethyl)amino)-5,6,7,9-tetrahydrobenzo[*a*]heptalen-7-yl)acetamide (**17b**)

Yellow solid (yield 75%); ^1^H NMR (300 MHz, DMSO-*d*_6_) δ 8.54 (d, *J* = 7.6 Hz, 1H), 7.80–7.68 (m, 3H), 7.61 (t, *J* = 5.9 Hz, 1H), 7.51 (d, *J* = 8.3 Hz, 2H), 7.38 (d, *J* = 8.1 Hz, 1H), 7.21 (d, *J* = 11.1 Hz, 1H), 7.07 (s, 1H), 6.82–6.71 (m, 2H), 4.41–4.30 (m, 1H), 3.82 (s, 3H), 3.77 (s, 3H), 3.67–3.59 (m, 2H), 3.47 (s, 3H), 3.04 (t, *J* = 7.2 Hz, 2H), 2.84–2.68 (m, 2H), 2.22–2.00 (m, 2H), 1.84 (s, 3H). ^13^C NMR (75 MHz, dmso) δ 174.84, 168.72, 154.23, 152.60, 151.00, 144.92, 143.81, 142.91, 141.99, 140.89, 138.49, 134.65, 129.49, 129.32, 126.01, 125.85, 123.76, 108.06, 63.68, 60.79, 56.85, 52.22, 44.06, 37.27, 33.07, 30.56, 24.20. C_29_H_33_N_3_O_7_S (567.2039), HRMS: m/z 568.2102 [M + H]^+^.

(*S*)-*N*-(10-((4-fluorobenzyl)amino)-1,2,3-trimethoxy-9-oxo-5,6,7,9-tetrahydrobenzo[*a*]heptalen-7-yl)acetamide (**18b**)

Yellow solid (yield 80%); ^1^H NMR (300 MHz, Chloroform-*d*) δ 7.54–7.46 (m, 2H), 7.40–7.27 (m, 4H), 7.12–7.02 (m, 2H), 6.57 (d, *J* = 11.2 Hz, 1H), 6.51 (s, 1H), 4.75–4.61 (m, 1H), 4.56 (d, *J* = 5.9 Hz, 2H), 3.92 (s, 3H), 3.88 (s, 3H), 3.60 (s, 3H), 2.51–2.33 (m, 2H), 2.31–2.20 (m, 1H), 1.99 (s, 3H), 1.93–1.84 (m, 1H). ^13^C NMR (75 MHz, cdcl_3_) δ 175.09, 169.65, 163.68, 163.35, 160.40, 153.53, 152.65, 151.25, 150.74, 141.25, 138.79, 134.14, 131.89, 130.88, 128.75, 128.64, 126.40, 123.33, 115.70, 115.41, 108.65, 106.91, 63.35, 61.04, 55.80, 52.34, 45.94, 36.81, 29.75, 22.52. C_28_H_29_FN_2_O_5_ (492.2061), HRMS: m/z 493.2134 [M + H]^+^.

(*S*)-*N*-(1,2,3-trimethoxy-10-((4-methoxybenzyl)amino)-9-oxo-5,6,7,9-tetrahydrobenzo[*a*]heptalen-7-yl)acetamide (**19b**)

Yellow solid (yield 83%); ^1^H NMR (300 MHz, Chloroform-*d*) δ 7.63 (d, *J* = 7.3 Hz, 1H), 7.57–7.47 (m, 2H), 7.41 (d, *J* = 11.2 Hz, 1H), 6.95–6.86 (m, 2H), 6.65 (d, *J* = 11.3 Hz, 1H), 6.52 (s, 1H), 4.74–4.64 (m, 1H), 4.51 (d, *J* = 5.5 Hz, 2H), 3.92 (s, 3H), 3.89 (s, 3H), 3.81 (s, 3H), 3.61 (s, 3H), 2.51–2.42 (m, 1H), 2.41–2.24 (m, 2H), 2.00 (s, 3H), 1.99–1.90 (m, 1H). ^13^C NMR (75 MHz, cdcl_3_) δ 174.94, 169.70, 158.97, 153.67, 152.59, 151.20, 150.73, 141.21, 138.88, 134.22, 130.61, 128.44, 128.05, 126.48, 123.08, 114.02, 108.70, 106.87, 62.40, 61.05, 56.19, 54.97, 52.71, 46.17, 36.80, 29.77, 22.51. C_29_H_32_N_2_O_6_ (504.2260), HRMS: m/z 505.2246 [M + H]^+^.

(*S*)-*N*-(10-((3,4-dimethoxyphenethyl)amino)-1,2,3-trimethoxy-9-oxo-5,6,7,9-tetrahydrobenzo[*a*]heptalen-7-yl)acetamide (**20b**)

Yellow solid (yield 88%); ^1^H NMR (300 MHz, Chloroform-*d*) δ 7.61 (d, *J* = 7.1 Hz, 1H), 7.53 (s, 1H), 7.45 (d, *J* = 11.2 Hz, 1H), 7.37–7.31 (m, 1H), 6.88–6.78 (m, 2H), 6.78–6.74 (m, 1H), 6.64 (d, *J* = 11.3 Hz, 1H), 6.53 (s, 1H), 4.76–4.64 (m, 1H), 3.94 (s, 3H), 3.89 (d, *J* = 4.5 Hz, 6H), 3.86 (s, 3H), 3.61 (s, 3H), 3.00 (t, *J* = 7.1 Hz, 2H), 2.53–2.43 (m, 2H), 2.40–2.26 (m, 2H), 2.25–2.03 (m, 1H), 1.98 (s, 3H), 1.97–1.86 (m, 1H). ^13^C NMR (75 MHz, cdcl_3_) δ 173.96, 169.70, 153.73, 152.66, 151.30, 150.71, 148.85, 147.65, 141.21, 139.18, 134.26, 130.68, 130.32, 126.42, 122.64, 120.35, 111.59, 111.23, 108.66, 106.94, 61.10, 61.05, 55.82, 55.59, 52.27, 43.99, 36.82, 34.11, 29.76, 29.34, 22.47. C_31_H_36_N_2_O_7_ (548.2523), HRMS: m/z 549.2596 [M + H]^+^.

(*S*)-*N*-(10-((4-fluorophenethyl)amino)-1,2,3-trimethoxy-9-oxo-5,6,7,9-tetrahydrobenzo[*a*]heptalen-7-yl)acetamide (**21b**)

Yellow solid (yield 88%); ^1^H NMR (300 MHz, Chloroform-*d*) δ 7.64 (d, *J* = 7.4 Hz, 1H), 7.43 (d, *J* = 10.6 Hz, 2H), 7.25–7.18 (m, 3H), 7.07–6.96 (m, 2H), 6.61 (d, *J* = 11.3 Hz, 1H), 6.54 (s, 1H), 4.75–4.65 (m, 1H), 3.94 (s, 3H), 3.90 (s, 3H), 3.64–3.54 (m, 5H), 3.02 (t, *J* = 7.2 Hz, 2H), 2.51–2.42 (m, 1H), 2.40–2.18 (m, 2H), 1.99 (s, 3H), 1.97–1.90 (m, 1H). ^13^C NMR (75 MHz, cdcl_3_) δ 175.16, 170.03, 163.58, 160.33, 154.09, 153.11, 151.54, 151.21, 141.72, 139.30, 134.65, 134.00, 133.96, 130.91, 130.34, 130.24, 126.93, 123.26, 115.89, 115.61, 108.63, 107.39, 61.49, 61.45, 56.28, 52.65, 44.18, 37.33, 34.15, 30.23, 22.98. C_29_H_31_FN_2_O_5_ (506.2217), HRMS: m/z 507.2291 [M + H]^+^.

(*S*)-*N*-(10-(cyclopentylamino)-1,2,3-trimethoxy-9-oxo-5,6,7,9-tetrahydrobenzo[*a*]heptalen-7-yl)acetamide (**22b**)

Yellow solid (yield 83%); ^1^H NMR (300 MHz, Chloroform-*d*) δ 7.54 (d, *J* = 7.0 Hz, 1H), 7.46–7.37 (m, 2H), 7.22 (d, *J* = 6.9 Hz, 1H), 6.64 (d, *J* = 11.6 Hz, 1H), 6.52 (s, 1H), 4.75–4.61 (m, 1H), 3.94 (s, 3H), 3.89 (s, 3H), 3.62 (s, 3H), 2.50–2.32 (m, 2H), 2.31–2.20 (m, 1H), 2.15–2.11 (m, 1H), 1.98 (s, 3H), 1.96–1.86 (m, 1H), 1.84–1.73 (m, 4H), 1.72–1.60 (m, 4H). ^13^C NMR (75 MHz, cdcl_3_) δ 174.61, 169.69, 153.55, 152.49, 150.82, 150.77, 141.21, 139.39, 134.29, 129.70, 126.68, 122.67, 108.74, 106.85, 61.04, 55.78, 53.95, 52.35, 36.79, 32.71, 29.79, 23.83, 22.93. C_26_H_32_N_2_O_5_ (452.2311), HRMS: m/z 453.2387 [M + H]^+^.

(*S*)-*N*-(1,2,3-trimethoxy-9-oxo-10-((2-(pyrrolidin-1-yl)ethyl)amino)-5,6,7,9-tetrahydrobenzo[*a*]heptalen-7-yl)acetamide (**23b**)

Yellow solid (yield 79%); ^1^H NMR (300 MHz, Chloroform-*d*) δ 7.66 (d, *J* = 7.2 Hz, 1H), 7.51 (t, 1H), 7.47 (s, 1H), 7.41 (d, *J* = 11.1 Hz, 1H), 6.59 (d, *J* = 11.3 Hz, 1H), 6.52 (s, 1H), 4.75–4.64 (m, 1H), 3.94 (s, 3H), 3.89 (s, 3H), 3.62 (s, 3H), 3.53–3.41 (m, 2H), 2.86 (t, *J* = 6.6 Hz, 2H), 2.62–2.56 (m, 4H), 2.49–2.33 (m, 2H), 2.30–2.18 (m, 1H), 1.98 (s, 3H), 1.94–1.87 (m, 1H), 1.84–1.78 (m, 4H). ^13^C NMR (75 MHz, cdcl_3_) δ 174.91, 169.64, 154.00, 152.50, 150.86, 150.78, 141.22, 138.74, 134.25, 129.87, 126.68, 122.77, 108.08, 106.87, 61.05, 55.83, 55.78, 53.71, 53.43, 52.20, 41.34, 36.79, 29.83, 23.26, 22.50. C_27_H_35_N_3_O_5_ (481.2577), HRMS: m/z 482.2652 [M + H]^+^.

(*S*)-*N*-(10-((2-(benzo[d][1,3]dioxol-5-yl)ethyl)amino)-1,2,3-trimethoxy-9-oxo-5,6,7,9-tetrahydrobenzo[*a*]heptalen-7-yl)acetamide (**24b**)

Yellow crystal (yield 78%); ^1^H NMR (300 MHz, Chloroform-*d*) δ 7.51 (s, 1H), 7.42 (d, 1H), 7.37–7.28 (m, 2H), 6.76 (d, 1H), 6.74–6.69 (m, 2H), 6.61 (d, *J* = 11.3 Hz, 1H), 6.53 (s, 1H), 5.94 (s, 2H), 4.75–4.64 (m, 1H), 3.94 (s, 3H), 3.90 (s, 3H), 3.61 (s, 3H), 3.60–3.53 (m, 2H), 2.97 (t, *J* = 7.2 Hz, 2H), 2.52–2.43 (m, 1H), 2.37–2.21 (m, 2H), 1.99 (s, 3H), 1.95–1.85 (m, 1H). ^13^C NMR (75 MHz, cdcl_3_) δ 174.60, 169.97, 154.01, 152.94, 151.49, 151.07, 147.97, 146.46, 141.56, 139.31, 134.57, 131.81, 130.83, 126.82, 123.04, 121.68, 108.97, 108.72, 108.49, 107.25, 100.98, 61.37, 60.34, 56.14, 52.54, 44.25, 37.14, 34.57, 30.09, 22.81. C_30_H_32_N_2_O_7_ (532.2210), HRMS: m/z 533.2286 [M + H]^+^.

(*S*)-*N*-(10-((2-(1H-indol-3-yl)ethyl)amino)-1,2,3-trimethoxy-9-oxo-5,6,7,9-tetrahydrobenzo[*a*]heptalen-7-yl)acetamide (**25b**)

Yellow solid (yield 75%); ^1^H NMR (300 MHz, Chloroform-*d*) δ 8.45 (s, 1H), 7.67–7.56 (m, 2H), 7.43–7.40 (m, 2H), 7.23–7.07 (m, 4H), 7.05 (d, *J* = 2.0 Hz, 1H), 6.62 (d, *J* = 11.3 Hz, 1H), 6.52 (s, 1H), 4.75–4.60 (m, 1H), 3.94 (s, 3H), 3.89 (s, 3H), 3.61 (s, 3H), 3.21 (t, *J* = 6.9 Hz, 2H), 3.09–2.99 (m, 1H), 2.97–2.87 (m, 1H), 2.46–2.29 (m, 2H), 2.23–2.15 (m, 1H), 1.92 (s, 3H), 1.88–1.81 (m, 1H). ^13^C NMR (75 MHz, cdcl_3_) δ 174.57, 169.86, 153.95, 152.53, 150.79, 150.67, 141.11, 139.06, 136.17, 134.28, 129.99, 126.69, 126.50, 122.39, 122.22, 121.73, 119.02, 118.02, 111.49, 111.20, 108.44, 106.88, 61.00, 55.76, 52.24, 42.60, 36.81, 29.71, 28.41, 24.17, 22.36. C_31_H_33_N_3_O_5_ (527.2420), HRMS: m/z 528.2497 [M + H]^+^.

(*S*)-*N*-(1,2,3-trimethoxy-10-((2-(5-methoxy-1H-indol-3-yl)ethyl)amino)-9-oxo-5,6,7,9-tetrahydrobenzo[*a*]heptalen-7-yl)acetamide (**26b**)

Yellow solid (yield 73%); ^1^H NMR (300 MHz, Chloroform-*d*) δ 8.35 (s, 1H), 7.58 (d, *J* = 7.2 Hz, 1H), 7.44 (d, *J* = 8.0 Hz, 2H), 7.39 (s, 1H), 7.04 (d, *J* = 3.0 Hz, 2H), 6.86 (d, *J* = 10.9 Hz, 1H), 6.64 (d, *J* = 11.3 Hz, 1H), 6.52 (s, 1H), 4.73–4.61 (m, 1H), 3.93 (s, 3H), 3.89 (s, 3H), 3.86 (s, 3H), 3.68 (d, *J* = 6.2 Hz, 2H), 3.61 (s, 3H), 3.20 (t, *J* = 7.1 Hz, 2H), 2.47–2.27 (m, 2H), 2.24–2.13 (m, 1H), 1.94 (s, 3H), 1.90–1.82 (m, 1H). ^13^C NMR (75 MHz, cdcl_3_) δ 174.56, 169.79, 153.93, 153.62, 152.53, 150.88, 150.68, 141.13, 139.00, 134.27, 131.32, 130.03, 127.09, 126.52, 122.92, 122.43, 113.49, 111.89, 111.27, 108.38, 106.86, 99.89, 61.68, 61.00, 55.76, 55.57, 52.22, 42.57, 36.76, 29.73, 24.21, 22.38. C_32_H_35_N_3_O_6_ (557.2526), HRMS: m/z 558.2603 [M + H]^+^.

*N*-((*S*)-10-((((*S*)-3-(3-fluoro-4-morpholinophenyl)-2-oxooxazolidin-5-yl)methyl)amino)-1,2,3-trimethoxy-9-oxo-5,6,7,9-tetrahydrobenzo[*a*]hept*a*len-7-yl)acetamide (**27b**)

Yellow solid (yield 80%); ^1^H NMR (300 MHz, Chloroform-*d*) δ 7.56–7.38 (m, 5H), 7.15–7.05 (m, 1H), 6.92 (t, *J* = 9.1 Hz, 1H), 6.69 (d, *J* = 11.2 Hz, 1H), 6.54 (s, 1H), 5.03–4.93 (m, 1H), 4.72–4.61 (m, 1H), 4.16 (t, *J* = 8.8 Hz, 1H), 3.93 (s, 3H), 3.89 (s, 3H), 3.87–3.83 (m, 4H), 3.83–3.75 (m, 3H), 3.61 (s, 3H), 3.08–2.99 (m, 4H), 2.40–2.20 (m, 2H), 1.99 (s, 3H), 1.93–1.87 (m, 2H). ^13^C NMR (75 MHz, cdcl_3_) δ 175.06, 169.70, 156.81, 154.33, 153.66, 153.41, 152.87, 151.70, 150.75, 141.26, 140.35, 138.60, 136.47, 136.35, 134.27, 132.64, 132.50, 131.84, 126.20, 123.93, 118.64, 113.22, 108.44, 107.52, 107.07, 70.52, 67.04, 61.15, 60.33, 55.87, 52.44, 50.70, 49.90, 48.07, 45.66, 35.89, 29.73, 23.16. C_35_H_39_FN_4_O_8_ (662.2752), HRMS: m/z 663.2830 [M + H]^+^.

### 3.2. Biological Studies

#### 3.2.1. Bacterial Strains and Chemical Reagents

The standard strain USA300 used in this experiment was purchased from the China General Microbiological Culture Collection Center (Beijing, China) and is stored in a −80 °C freezer at Jilin University Laboratory.

#### 3.2.2. Biofilm Inhibition Assay

A 96-well plate was coated with lyophilized rabbit plasma at 4 °C for 12 h. Then the liquid was discarded, and the plates were dried at room temperature for later use. MRSA USA300 stored at −80 °C was activated using solid BHI (Shanghai yuanye Bio-Technology Co, Ltd, Shanghai, China) medium. Then, single colonies were picked and inoculated into BHI medium supplemented with 3% sucrose and incubated at 200 rpm/min for 12 h at 37 °C on a shaker for secondary activation. The cultured broth was diluted 1:100 in BHI (containing 3% sucrose) and set aside. Then, diluted bacterial solution, compounds 1–27b, and colchicine were added to the treated 96-well plate, row C, well 1, and mixed well, and 100 μL of diluted bacterial solution was added to wells 2 to 11 for multiplicative dilution. Row B was incubated with only 100 μL of diluted bacterial solution as a negative control in a biochemical incubator at 37 °C for 12 h. After incubation, the 96-well plates were removed from the incubator, the culture medium was discarded, and the plates were rinsed slowly with sterile PBS twice to remove the unadhered bacteria. The plates were dried at room temperature. Each well was then stained with 200 µL of 1% crystal violet (Sigma-Aldrich, Shanghai, China) for 15 min at room temperature. After washing three times with PBS (Solarbio, Beijing, China) and drying at room temperature, the crystal violet was dissolved using a 33% glacial acetic acid solution (Solarbio). The absorbance value at 595 nm was measured using a microplate reader (TECAN, Beijing, China). The inhibition rate was calculated as follows: Inhibition percentage = [(Control OD_595_ - Treatment OD_595_)/Control OD_595_] × 100% [32].

#### 3.2.3. Determination of MIC and FICI

Minimum inhibitory concentrations (MICs) were determined using the broth microdilution method in accordance with the Clinical and Laboratory Standards Institute Guidelines [33]. MRSA USA300 was cultured in LB broth overnight. The cultured bacterial solution was inoculated into test tubes containing CAMHB (Shanghai yuanye Bio-Technology Co, Ltd) and incubated at 37 °C with shaking at 180 rpm. When the OD_600_ reached 1.0, the culture was taken out for further use. A sterile 96-well plate was prepared, with 100 µL of sterile CAMHB added to each well in columns 1 to 11. In column 12, 200 µL of sterile CAMHB was added to each well, followed by the addition of a certain amount of drug stock solution to achieve a final drug concentration of 1024 µg/mL in that column. Subsequent multiplicative dilutions of the drug were performed from right to left. Next, the bacterial solution was diluted at a ratio of 1:100 and added to all of the wells of the 96-well plate. Finally, the sealed 96-well plate was placed in a constant temperature incubator and incubated at 37 °C for 16 h. The OD value was measured at 600 nm using a multifunctional enzyme marker (TECAN, Beijing, China).

The checkerboard method was used to determine the fractional inhibitory concentration index (FICI) of different compounds in combination with penicillin G and cefoxitin (Shanghai yuanye Bio-Technology Co, Ltd). MRSA USA300 that had been cultured overnight was inoculated into test tubes containing CAMHB and incubated at 37 °C with shaking at 180 rpm. When the OD_600_ reached 1.0, the culture was taken out for further use. In a 96-well plate, penicillin G sodium solution was multiplicative diluted starting from the final concentration of MIC along the vertical axis to obtain various concentrations of penicillin G. The compounds were multiplicative diluted from the highest final concentration (MIC) along the horizontal axis, from right to left. Next, the bacterial solution was diluted at a ratio of 1:100 and added to all of the wells of the 96-well plate. Finally, the sealed 96-well plate was placed in a constant temperature incubator and incubated at 37 °C for 16 h. The OD value was measured at 600 nm using a multifunctional enzyme marker to determine the newly measured MIC values of the two drugs.

Formula for calculating the FICI:

FICI = FIC A + FIC B (1)

FIC A is the newly measured MIC of drug A divided by the MIC of drug A when used alone, and FIC B is the newly measured MIC of drug B divided by the MIC of drug B when used alone. When FICI ≤ 0.5, the two drugs are considered to have a synergistic effect. When 0.5 < FICI ≤ 1, they are considered to have an additive effect. When 1 < FICI ≤ 2, they are considered to have an indifferent effect; and when FICI > 2, they are considered to have an antagonistic effect.

#### 3.2.4. Biofilm Formation Experiment

Biofilm formation process of MRSA USA300: The MRSA USA300 bacterial solution incubated for 12 h was diluted with BHI (containing 3% sucrose) medium at a ratio of 1: 100, and then 200 μL per well was added to 96-well plates treated as described previously. The samples were taken out at different time points and stained with crystal violet as described above. The OD value at 595 nm was measured using a multifunctional enzyme marker.

Effects of **7b–11b** on the development of MRSA USA300 biofilm: A biofilm was cultivated according to the method described above, with the compound added at different time points. The culture was stopped after 24 h. Crystal violet staining was performed as described above, and the OD value at 595 nm was measured using a multifunctional enzyme marker.

#### 3.2.5. qPCR Analysis

MRSA USA300 stored at −80 °C was activated using BHI solid medium, and then single colonies were picked and inoculated in BHI medium supplemented with 3% sucrose, then incubated at 200 rpm/min for 12 h at 37 °C on a shaker for secondary activation. The resulting 12 h culture was then diluted 1:100 in BHI medium (containing 3% sucrose) and treated with different 1/2 MIC concentrations of **7b–11b**, followed by incubation at 200 rpm and 37 °C for another 12 h. RNA extraction was performed using TRIzol reagent (TIANGEN, Beijing, China). Single-stranded complementary DNA (cDNA) was reverse transcribed using a Seven Biotech All-in-one First Strand cDNA Synthesis Kit II Transcription Kit (Beijing, China) and amplified using Servicebio 2×Universal Blue SYBR Green qPCR Master Mix cDNA (Wuhan, China). The expression levels of the tested genes were normalized to 16S rRNA, and the relative expression levels of the genes were calculated using the 2−∆∆CT method. The primers used in this study are listed in the Supplementary Materials.

#### 3.2.6. Scanning Electron Microscopy Observation of Biofilm

Scanning electron microscopy (SEM) was used to investigate the effect of the preferred compounds on the structure of bacterial biofilms. The cell culture slides were placed in lyophilized rabbit plasma and immersed overnight at 4 °C. The overnight culture was diluted 1:100 in BHI medium (containing 3% sucrose). The soaked cell culture slides were dried in a clean bench for 1 h, after which the diluted culture was added to a 6-well plate. Both a compound treatment group (1/2 MIC) and a control group without compounds were set up and incubated at 37 °C for 12 h. After incubation, the 6-well plate was removed, and the culture medium was gently aspirated using a micropipette. The wells were then slowly rinsed with sterile PBS to remove non-adhered bacteria, followed by overnight fixation with glutaraldehyde at 4 °C. The results were observed using a scanning electron microscope [32].

### 3.3. ADME Studies

Absorption, distribution, metabolism, and excretion (ADME) properties were evaluated using the SwissADME [34] online software (http://www.swissadme.ch, accessed on 8 October 2024).

### 3.4. Statistical Analyses

All experiments were carried in triplicate and the findings were expressed as the mean ± standard error. Unless otherwise stated, statistical analyses were conducted with GraphPad Prism 5 software using Student’s *t*-tests or one-way ANOVA.

## 4. Conclusions

This study designed and synthesized 27 colchicine-derived amine derivatives with modifications on the C-ring (compounds **1b–27b**) and screened their inhibitory activity against MRSA USA300 biofilm formation. Most of the newly reported compounds exhibited varying degrees of biofilm inhibition, with inhibition rates ranging from 2.06% to 40.79%. The SAR analysis regarding biofilm inhibition revealed that compounds with aliphatic side chains (*n* = 4–9, including compound **1b** with *n* = 3 and a hydroxyl group at the end) had a higher likelihood of exhibiting biofilm inhibition. Compounds containing aromatic side chains also showed biofilm inhibition potential, particularly those with para-F and para-OCH3 modifications on a phenyl ring (*n* = 1), as well as indole ring modifications (*n* = 2) and hybrid molecules combining effective fragments of linezolid. Among these biofilm-inhibiting compounds, those with aliphatic single-chain side groups are more likely to demonstrate both antibacterial activity and synergistic antibacterial effects (*n* = 5–9). Specifically, when the aliphatic single-chain consists of 7–9 carbon atoms, antibacterial activity is more likely to be observed (MICs = 16–32 μg/mL). Notably, compound 9b (*n* = 7) exhibits the most potent antibacterial activity (MIC = 16 μg/mL). In contrast, compounds 10b and 11b (*n* = 8 and *n* = 9, respectively) have lower antibacterial activity compared to 9b but show synergistic antibacterial effects. This finding indicates that antibacterial efficacy does not necessarily increase with the increasing number of carbon atoms, which might suggest the tolerance level of this modification site. When the aliphatic chain is a single-chain with 5–6 carbon atoms or a double-chain with 8–10 carbon atoms, synergistic antibacterial effects are more likely to occur (FICI < 0.5). Based on the aforementioned observations, lipophilicity appears to contribute more significantly to synergistic antibacterial activity. However, a more systematic SAR study is still lacking, and this warrants further investigation in future research. Additionally, experiments on biofilm formation indicate that compounds **7b–11b** could inhibit the bacterial proliferation and maturation phases of MRSA USA300 biofilm development. qPCR experiments further demonstrated that compounds **7b–11b** can interfere with MRSA biofilm formation by regulating the expression of biofilm-related genes icaA and agrA.

In summary, given the promising results achieved thus far, compounds **9b–11b** have exhibited remarkable potential as lead candidates for MRSA biofilm inhibitors. For future research, we will concentrate on two key aspects. First, we will explore further structural modifications to optimize the properties of these compounds, aiming to develop more potent and less toxic inhibitors. This will involve a systematic study of substitution patterns and functional group modifications to enhance inhibitory activity and selectivity. Second, we plan to conduct in vitro and in vivo preclinical studies to evaluate the safety and efficacy of these potential inhibitors. These studies will provide crucial insights into the pharmacokinetics and pharmacodynamics of the compounds, laying a solid foundation for their translation into clinical applications.

## Figures and Tables

**Table 1:** Structures of new colchicine derivatives **1b–27b**.

Compound	n_1_	R_1_	n_2_	R_2_	%Yield
**1b**	3	-OH	0	-H	73
**2b**	2	-OCH_3_	0	-H	77
**3b**	3	-OCH_3_	0	-H	75
**4b**	2	-N(CH_3_)_2_	0	-H	65
**5b**	1	-CH(OCH_3_)_2_	0	-H	60
**6b**	4	-H	0	-H	88
**7b**	5	-H	0	-H	85
**8b**	6	-H	0	-H	80
**9b**	7	-H	0	-H	79
**10b**	8	-H	0	-H	75
**11b**	9	-H	0	-H	77
**12b**	2	-SCH_3_	0	-H	80
**13b**	3	-SCH_3_	0	-H	75
**14b**	4	-H	4	-H	67
**15b**	2	-OCH_3_	2	-OCH_3_	69
**16b**	5	-H	5	-H	61
**17b**	2	** 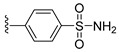 **	0	-H	**75**
**18b**	1	** 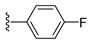 **	0	-H	80
**19b**	1	** 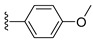 **	0	-H	83
**20b**	2	** 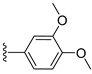 **	0	-H	88
**21b**	2	** 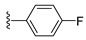 **	0	-H	88
**22b**	0	** 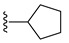 **	0	-H	83
**23b**	2	** 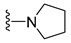 **	0	-H	79
**24b**	2	** 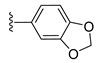 **	0	-H	78
**25b**	2	** 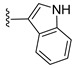 **	0	-H	75
**26b**	2	** 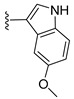 **	0	-H	73
**27b**	1	** 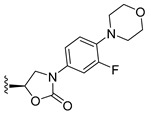 **	0	-H	80

**Table 2:** MICs and FICIs of 1b−27b.

Compound	MIC(μg/mL)	FICI	
		Penicillin G	Cefoxitin
**Colchicine**	>1024	>0.5	>0.5
**1b**	>1024	>0.5	>0.5
**2b**	>1024	>0.5	>0.5
**3b**	>1024	>0.5	>0.5
**4b**	>1024	>0.5	>0.5
**5b**	>1024	>0.5	>0.5
**6b**	>1024	>0.5	>0.5
**7b**	>1024	0.156	>0.5
**8b**	>1024	0.156	>0.5
**9b**	16	>0.5	>0.5
**10b**	32	0.375	>0.5
**11b**	32	0.3125	>0.5
**12b**	>1024	>0.5	>0.5
**13b**	>1024	>0.5	>0.5
**14b**	>1024	0.188	>0.5
**15b**	>1024	>0.5	>0.5
**16b**	>1024	0.156	0.266
**17b**	>1024	>0.5	>0.5
**18b**	>1024	>0.5	>0.5
**19b**	>1024	>0.5	>0.5
**20b**	>1024	>0.5	>0.5
**21b**	>1024	>0.5	>0.5
**22b**	>1024	>0.5	>0.5
**23**	>1024	>0.5	>0.5
**24b**	>1024	>0.5	>0.5
**25b**	>1024	>0.5	>0.5
**26b**	>1024	>0.5	>0.5
**27b**	>1024	>0.5	>0.5

**Table 3:** Other predicted properties of colchicine derivatives (**9b−11b**).

Compound	TPSA	Log S (ESOL)	Water Solubility Class (ESOL)^a^	Consensus Log Po/w	Log Kp (Skin Permeation)
**9b**	85.89	−5.44	2	4.48	−5.72
**10b**	85.89	−5.79	2	4.84	−5.42
**11b**	85.89	−6.15	3	5.20	−5.13

^a^ Water Solubility Class (ESOL): 1 = Soluble, 2 = Moderately soluble, 3 = Poorly soluble.

## Data Availability

The original contributions presented in this study are included in the article and Supplementary Material. Further inquiries can be directed to the corresponding authors.

## Supplementary Materials

The following supporting information can be downloaded at: https://www.mdpi.com/article/10.3390/antibiotics14020173/s1, Figure S1–S27: ^1^H-NMR, ^13^C-NMR, and HRMS data of compound 1b–27b; Table S1: Primer information for qPCR analysis of the selected genes.
