# Supplementary material for: Synthesis and Evaluation of Colchicine C-Cyclic AmineDerivatives as Potent Anti-Biofilms Agents AgainstMethicillin-Resistant Staphylococcus aureus
Source: Antibiotics (Basel). 2025 Feb 10;14(2):173. doi: 10.3390/antibiotics14020173 (PMC11851440; doi:10.3390/antibiotics14020173)
Supplement: Supplementary file 1 [file antibiotics-14-00173-s001.zip › antibiotics-3429828-supplementary.pdf]

Synthesis and evaluation of Colchicine C-cyclic amine derivatives as potent anti-biofilms agents against Methicillin-resistant *Staphylococcus aureus*

Yuxin Yang[1,2,#], Xin Liu[1,#], Can Sun[1], Yuan Fang[2], Danyang Qu[1], Zhengbin Tang[2],Zetao Sun[1] Xiaoping Zhou[2,*], Dacheng Wang[1,*]

[1]College of Animal Science, Jilin University, Changchun, China.
[2]School of Pharmaceutical Science, Jilin University, Changchun, China.

[#] These authors contributed equally to this work.

*Corresponding author: Dacheng Wang, Email: wangdc@jlu.edu.cn; Xiaoping Zhou, Email: zhouxp@jlu.edu.cn

Contents

1. ^{1}H-NMR, ^{13}C-NMR and HRMS Spectra data (Figure S1-S27)
2. Primer information for qPCR analysis of the selected genes (Table S1)

Figure S1. ^{1}H-NMR, ^{13}C-NMR and HRMS data of compound 1b

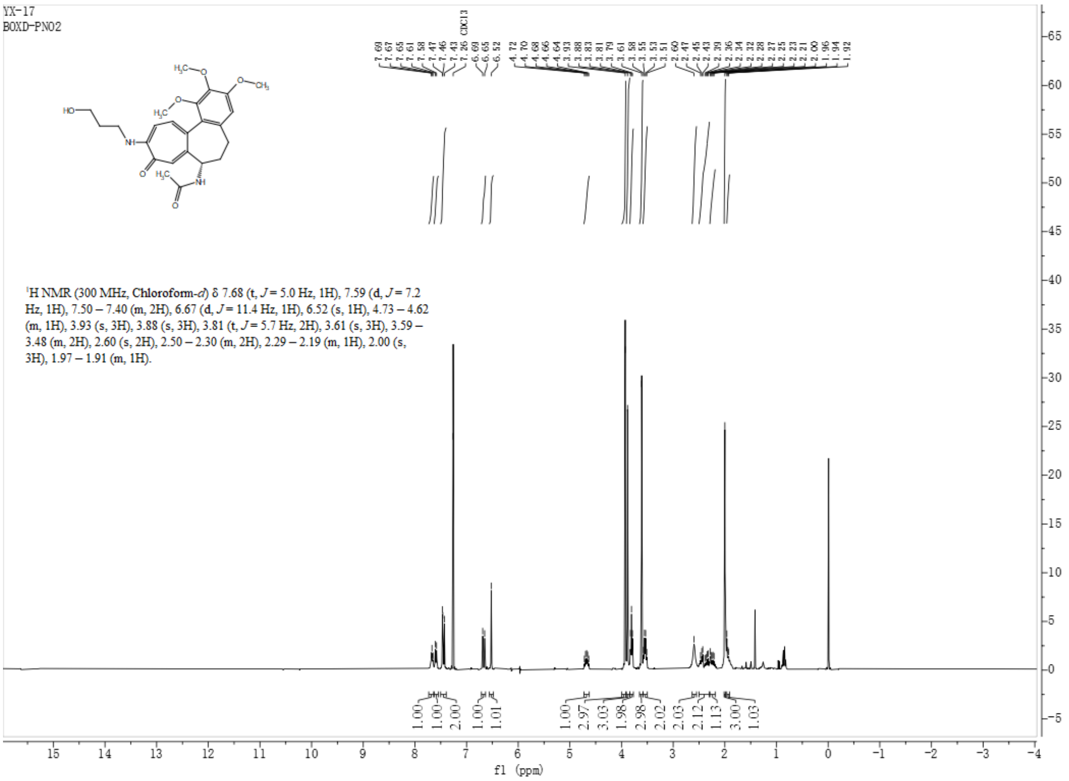

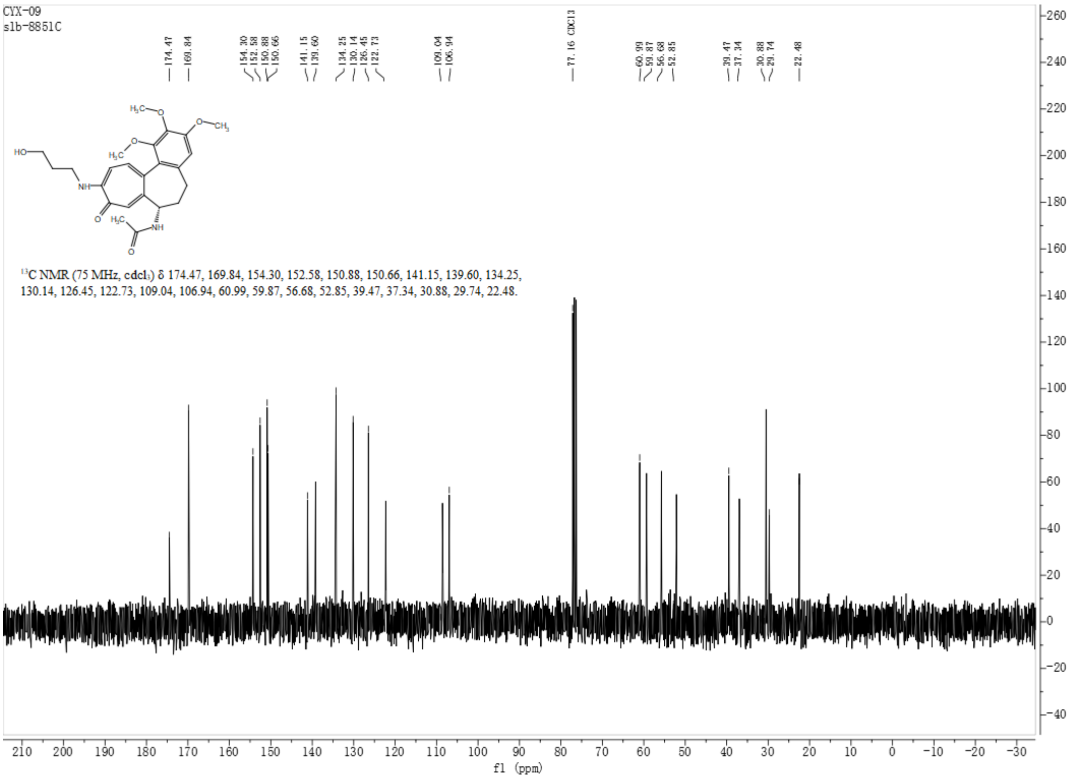

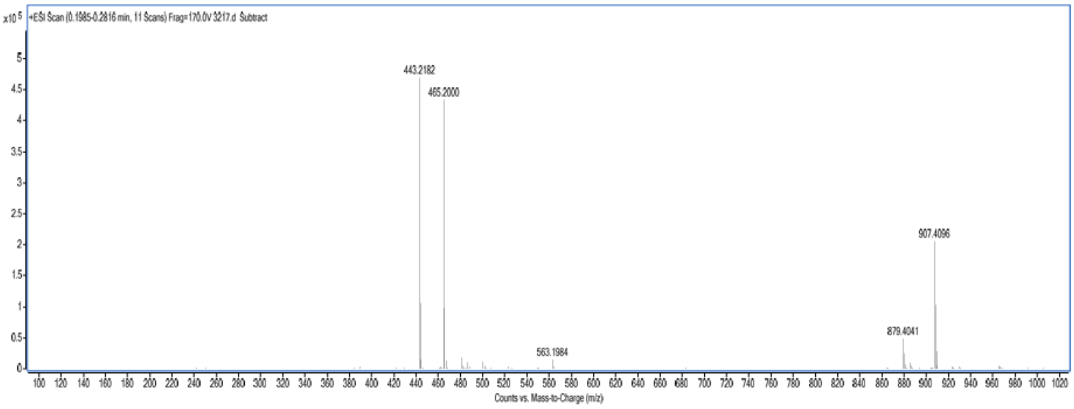

Figure S2. ^{1}H-NMR, ^{13}C-NMR and HRMS data of compound 2b

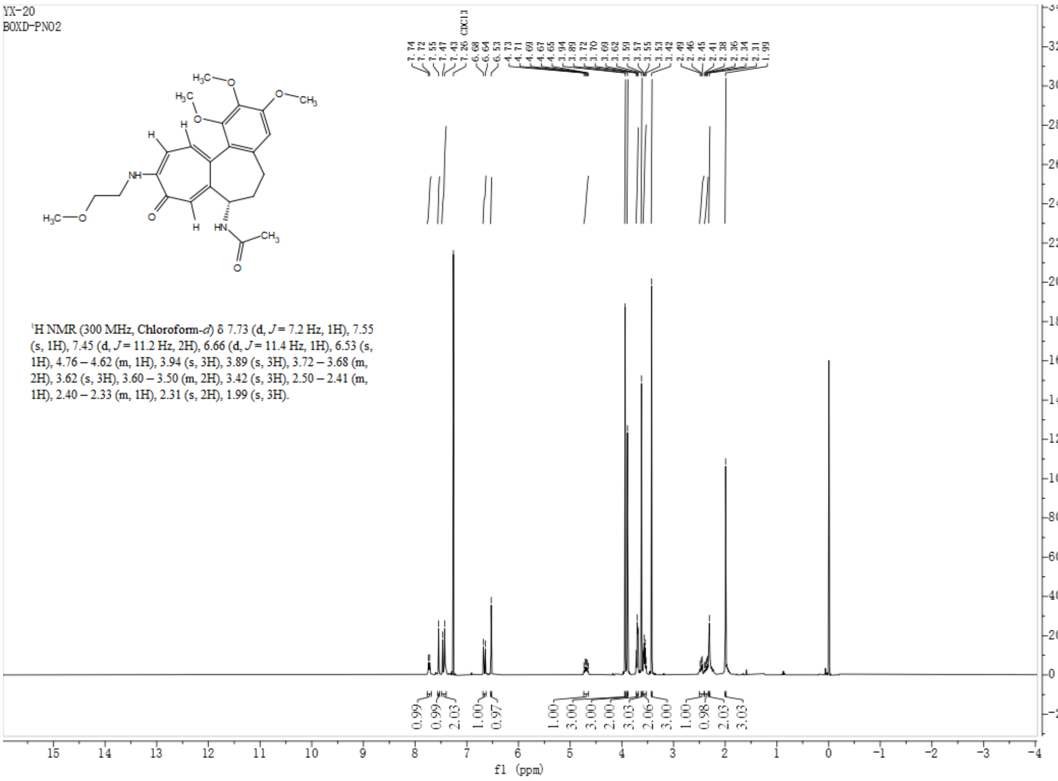

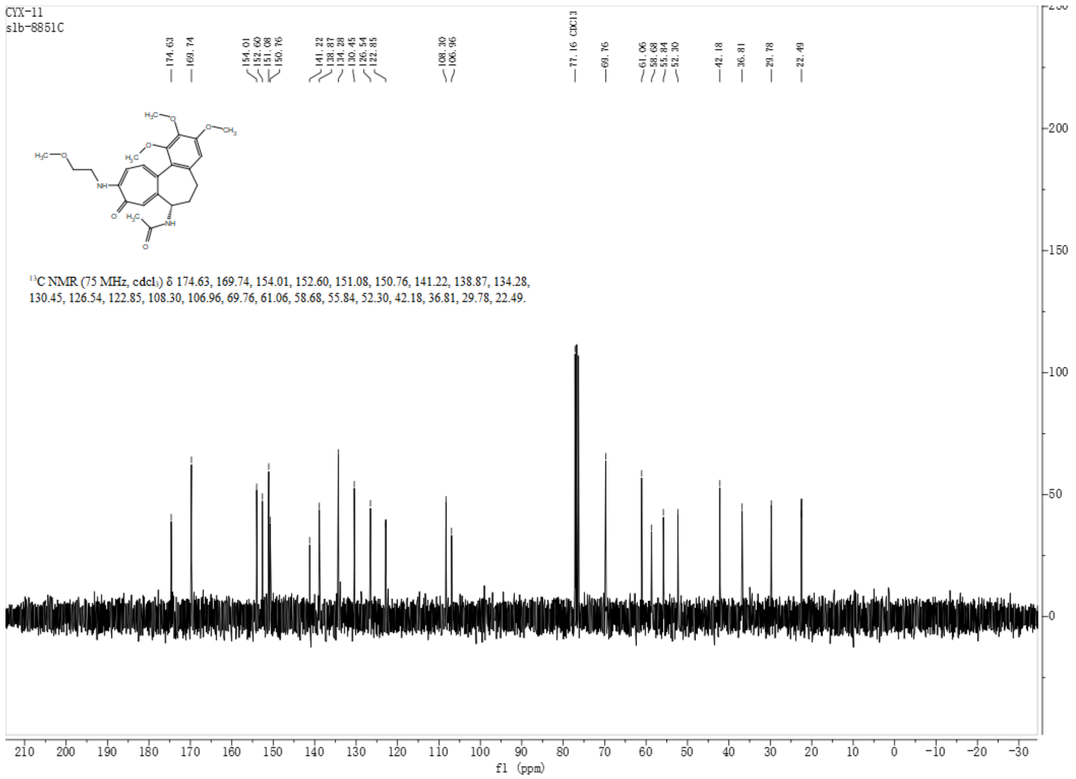
CYX-11
slb-8851C
174.63
169.74
154.01
152.60
151.08
150.76
141.22
138.87
134.28
130.45
126.54
122.85
108.30
106.96
77.16 CDCl3
69.76
61.06
58.68
55.84
52.30
42.18
36.81
29.78
22.49
^{13}C NMR (75 MHz, cdcl$_3$) δ 174.63, 169.74, 154.01, 152.60, 151.08, 150.76, 141.22, 138.87, 134.28, 130.45, 126.54, 122.85, 108.30, 106.96, 69.76, 61.06, 58.68, 55.84, 52.30, 42.18, 36.81, 29.78, 22.49.
f1 (ppm)

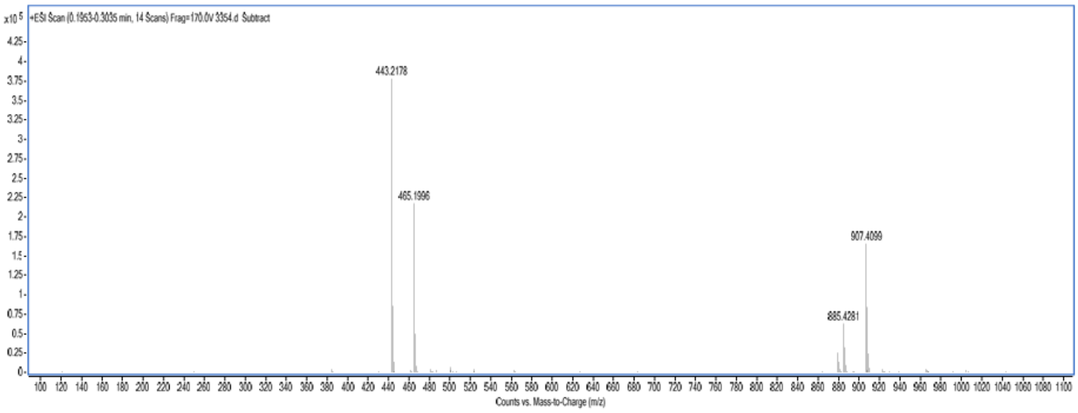
+ESI Scan (0.1953-0.3035 min, 14 Scans) Frag=170.0V 3354.d Subtract
443.2178
465.1996
907.4099
885.4281
Counts vs. Mass-to-Charge (m/z)

Figure S3. ^{1}H-NMR, ^{13}C-NMR and HRMS data of compound 3b

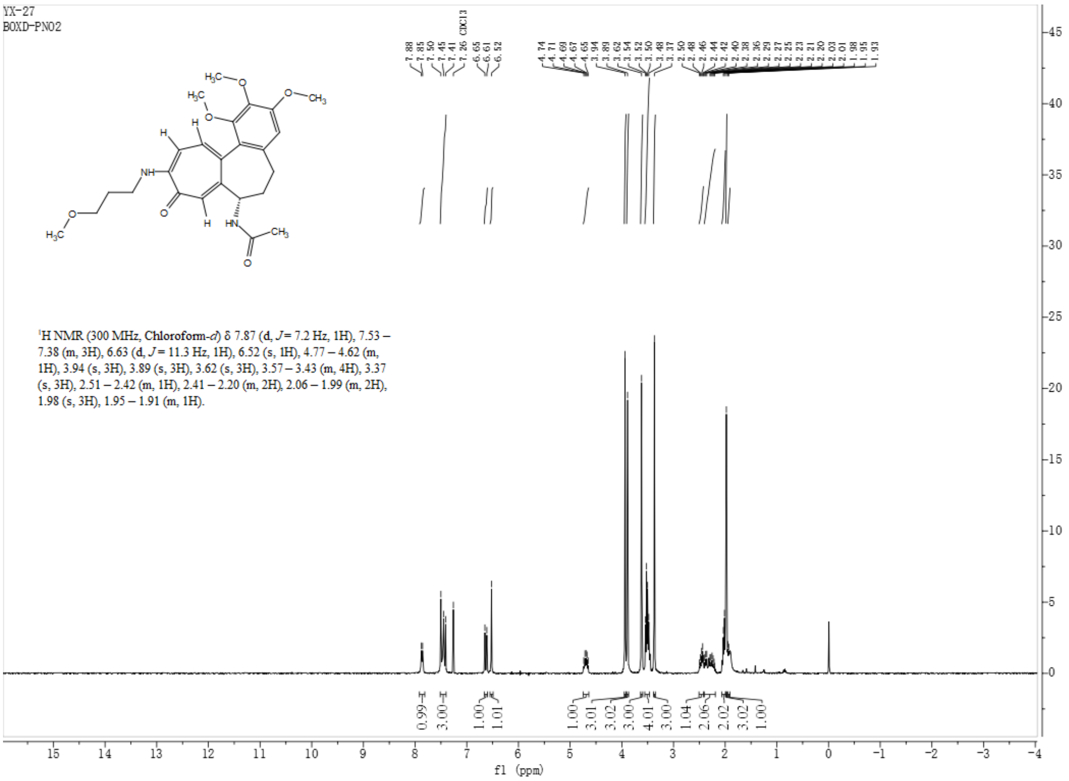

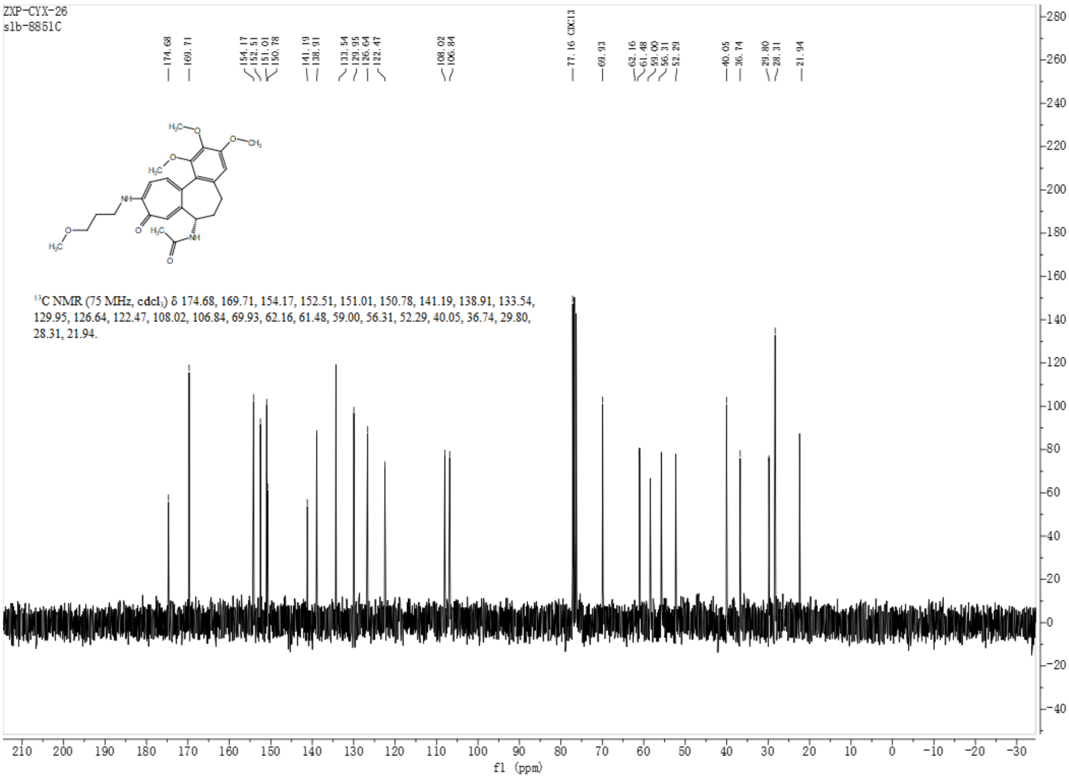

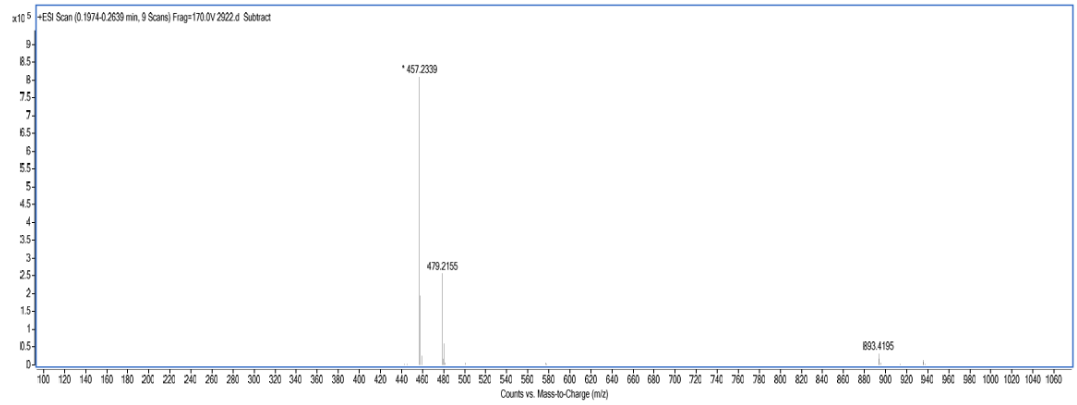

Figure S4. ^{1}H-NMR, ^{13}C-NMR and HRMS data of compound 4b

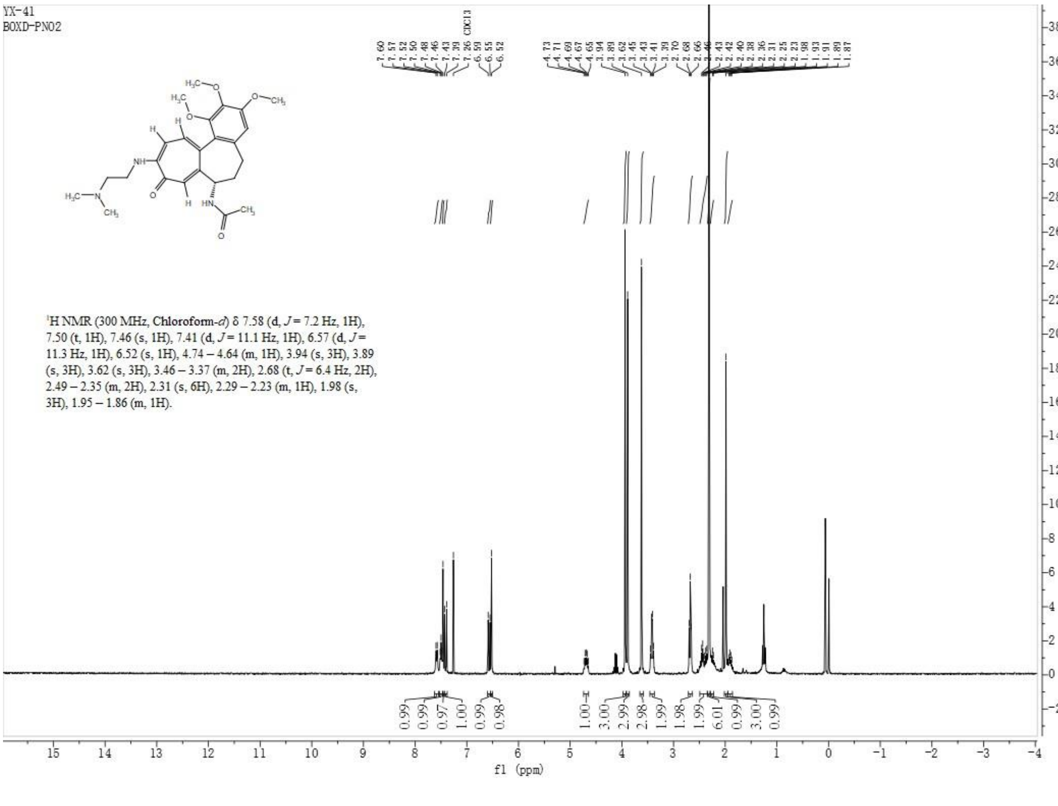

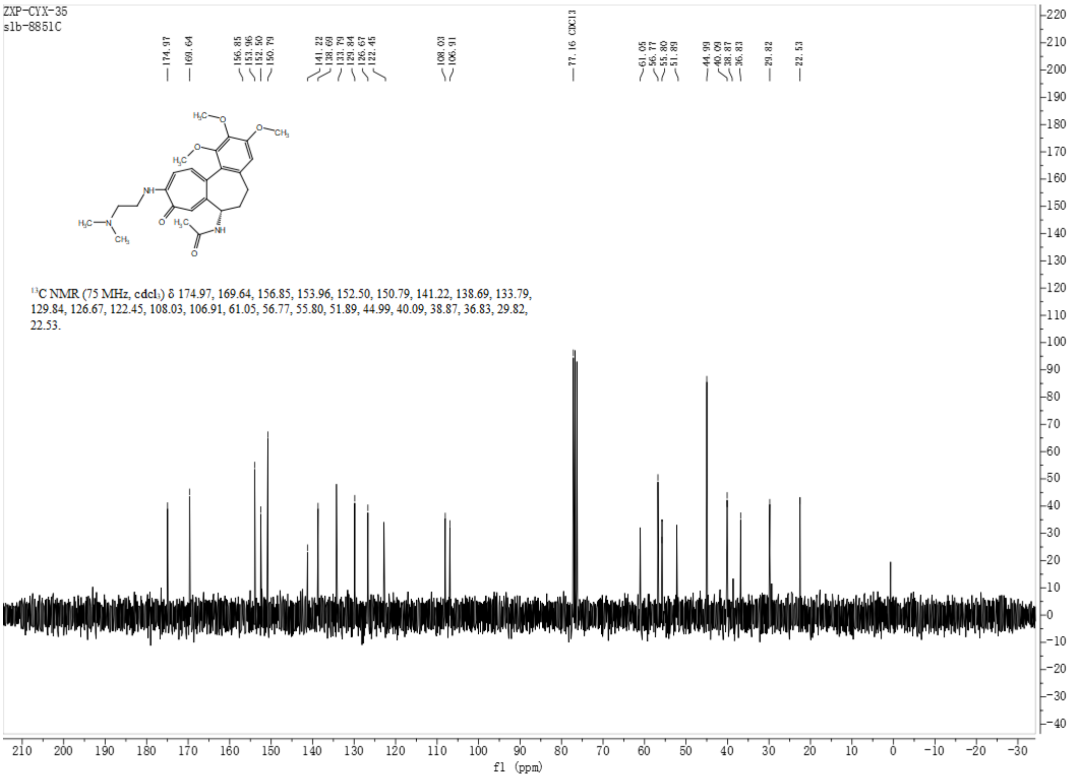
ZXP-CYX-35
slb-8851C
77.16 CDCl3
13C NMR (75 MHz, cdcl3) δ 174.97, 169.64, 156.85, 153.96, 152.50, 150.79, 141.22, 138.69, 133.79, 129.84, 126.67, 122.45, 108.03, 106.91, 61.05, 56.77, 55.80, 51.89, 44.99, 40.09, 38.87, 36.83, 29.82, 22.53.
f1 (ppm)

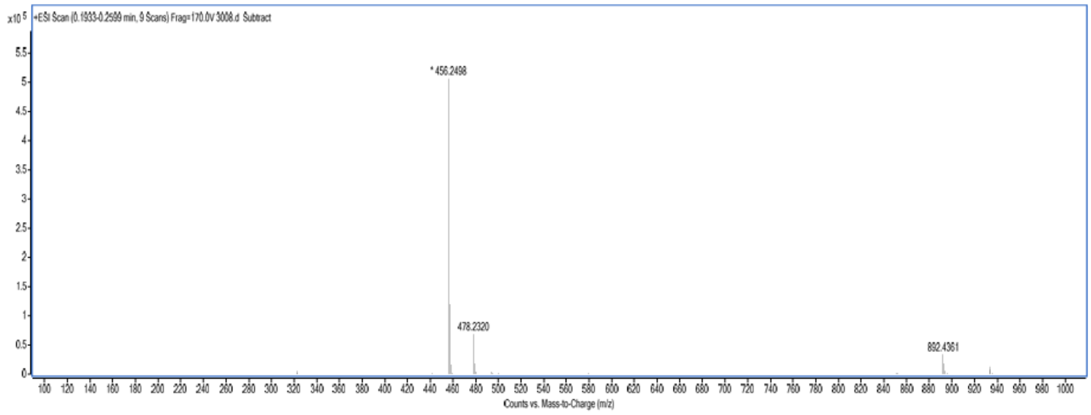
+ESI Scan (0.1933-0.2599 min, 9 Scans) Frag=170.0V 3008.d Subtract
x10 5
* 456.2498
478.2320
892.4361
Counts vs. Mass-to-Charge (m/z)

Figure S5. ^{1}H-NMR, ^{13}C-NMR and HRMS data of compound 5b

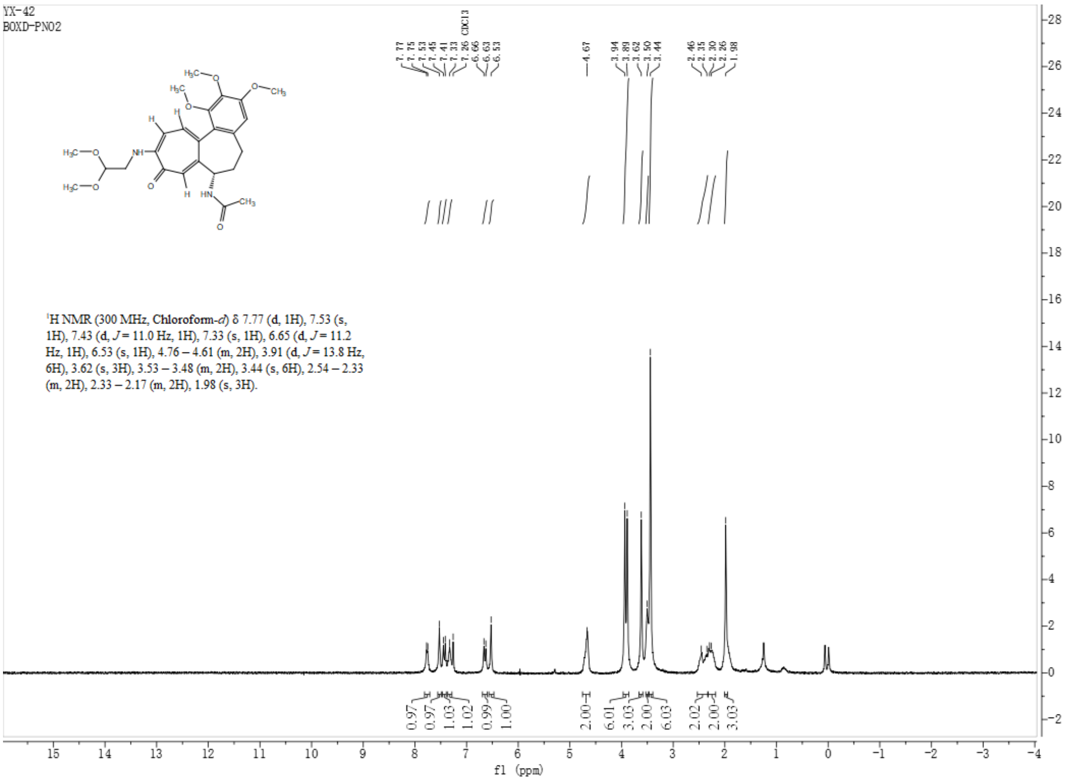

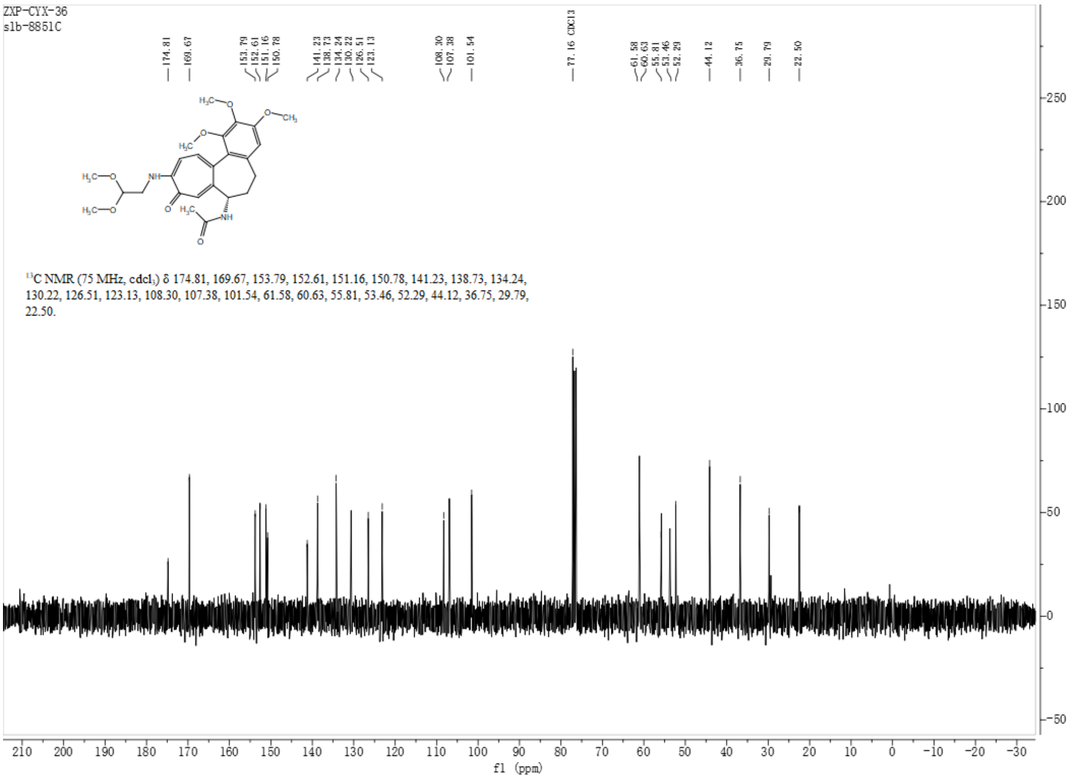

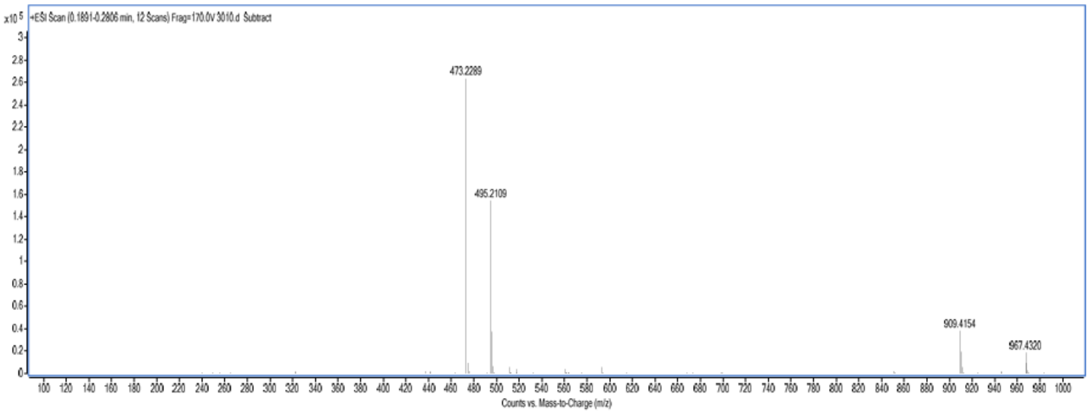

Figure S6. ^{1}H-NMR, ^{13}C-NMR and HRMS data of compound 6b

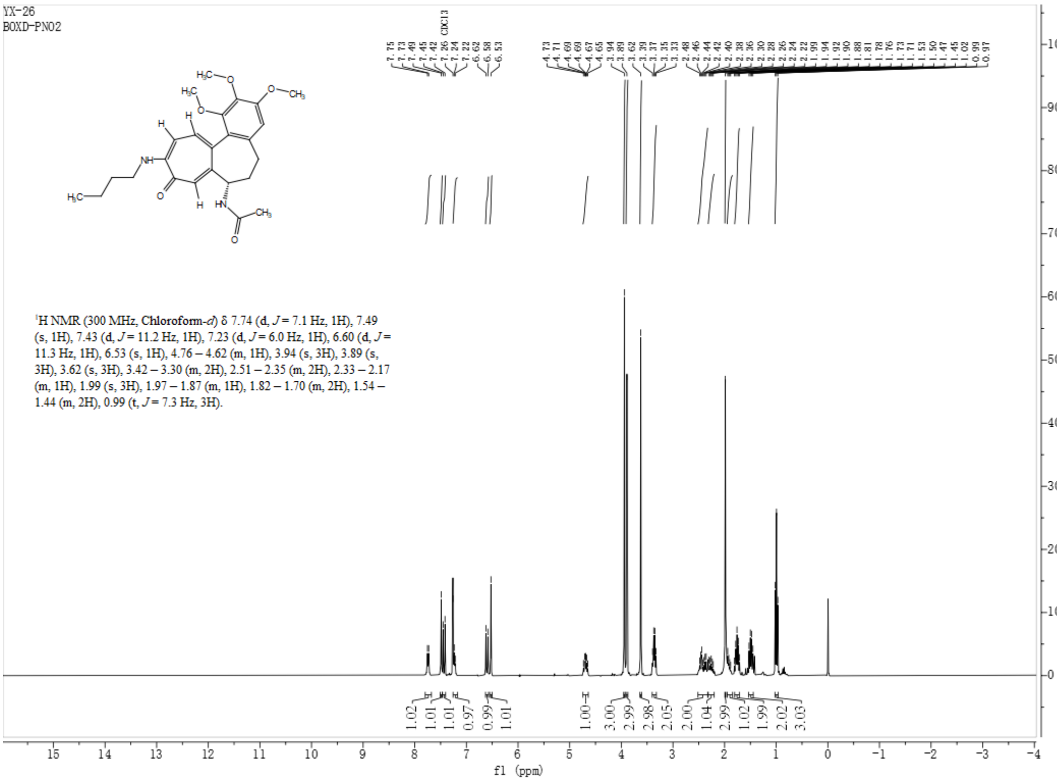

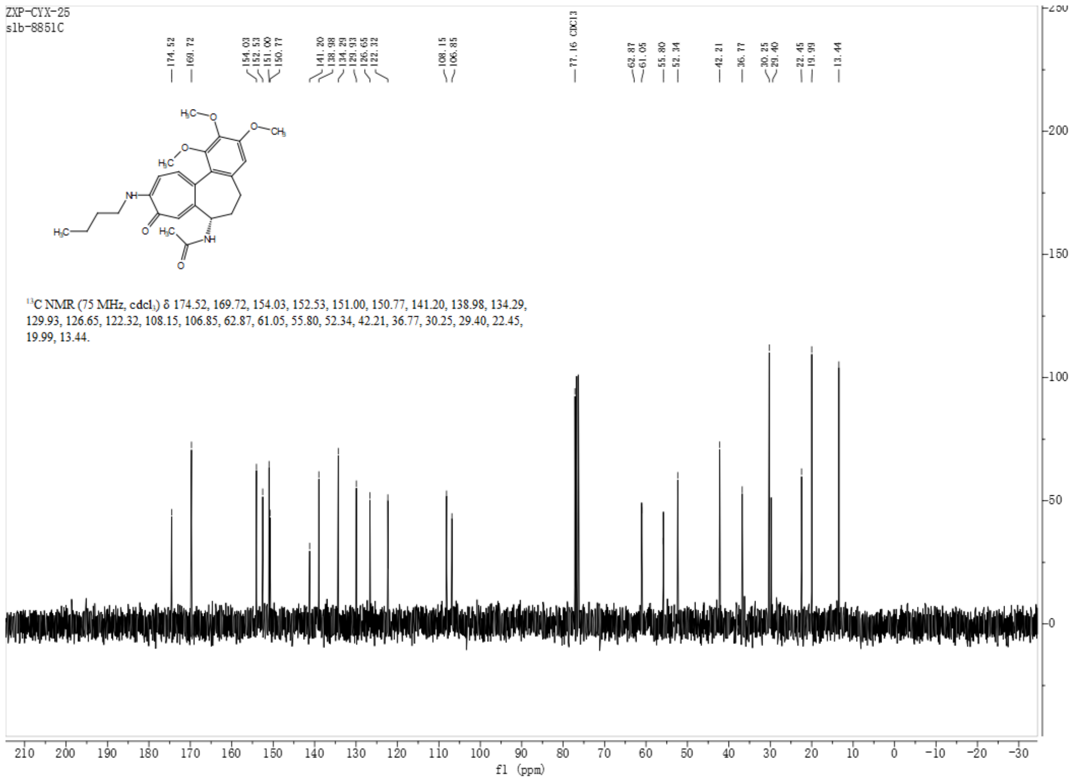
ZXP-CYX-25
slb-8851C
77.16 CDCl3
13C NMR (75 MHz, cdcl3) δ 174.52, 169.72, 154.03, 152.53, 151.00, 150.77, 141.20, 138.98, 134.29, 129.93, 126.65, 122.32, 108.15, 106.85, 62.87, 61.05, 55.80, 52.34, 42.21, 36.77, 30.25, 29.40, 22.45, 19.99, 13.44.
f1 (ppm)

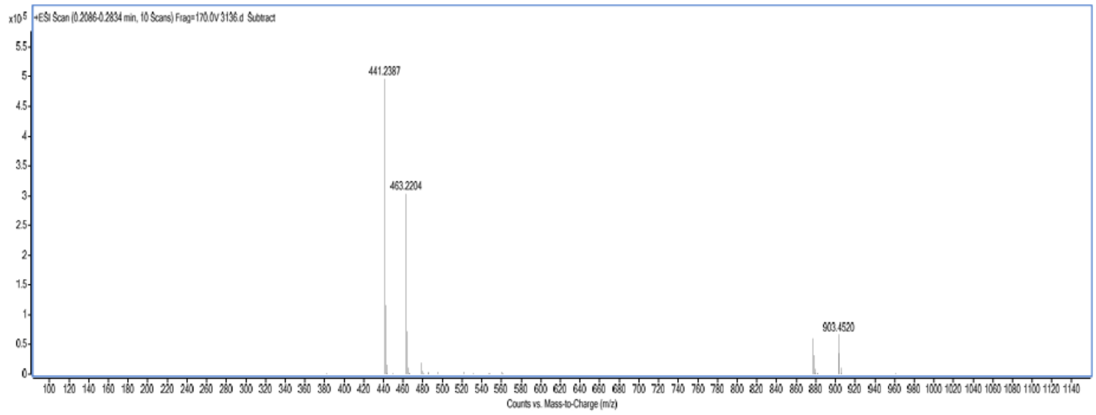
x10^5
+ESI Scan (0.2086-0.2834 min, 10 Scans) Frag=170.0V 3136.d Subtract
441.2387
463.2204
903.4520
Counts vs. Mass-to-Charge (m/z)

Figure S7. ^{1}H-NMR, ^{13}C-NMR and HRMS data of compound 7b

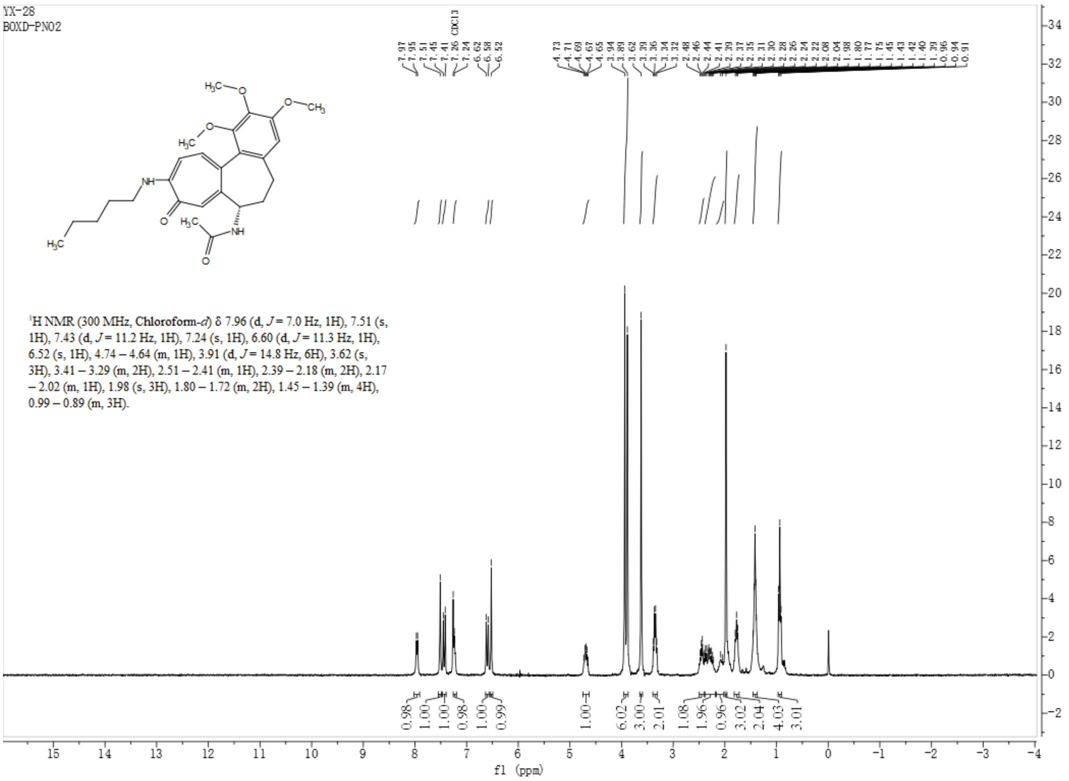

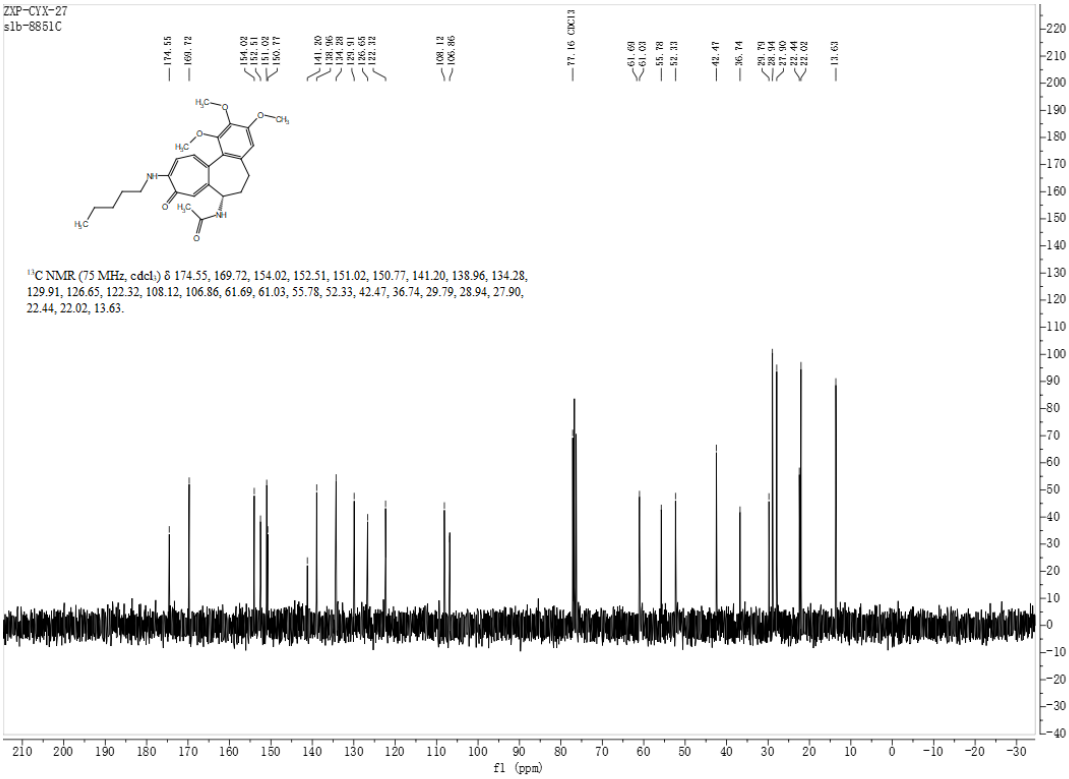

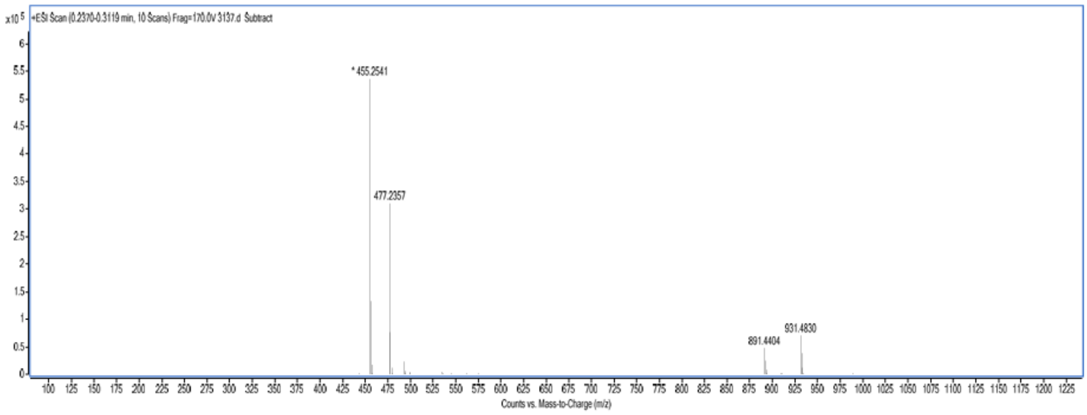

Figure S8. ^{1}H-NMR, ^{13}C-NMR and HRMS data of compound 8b

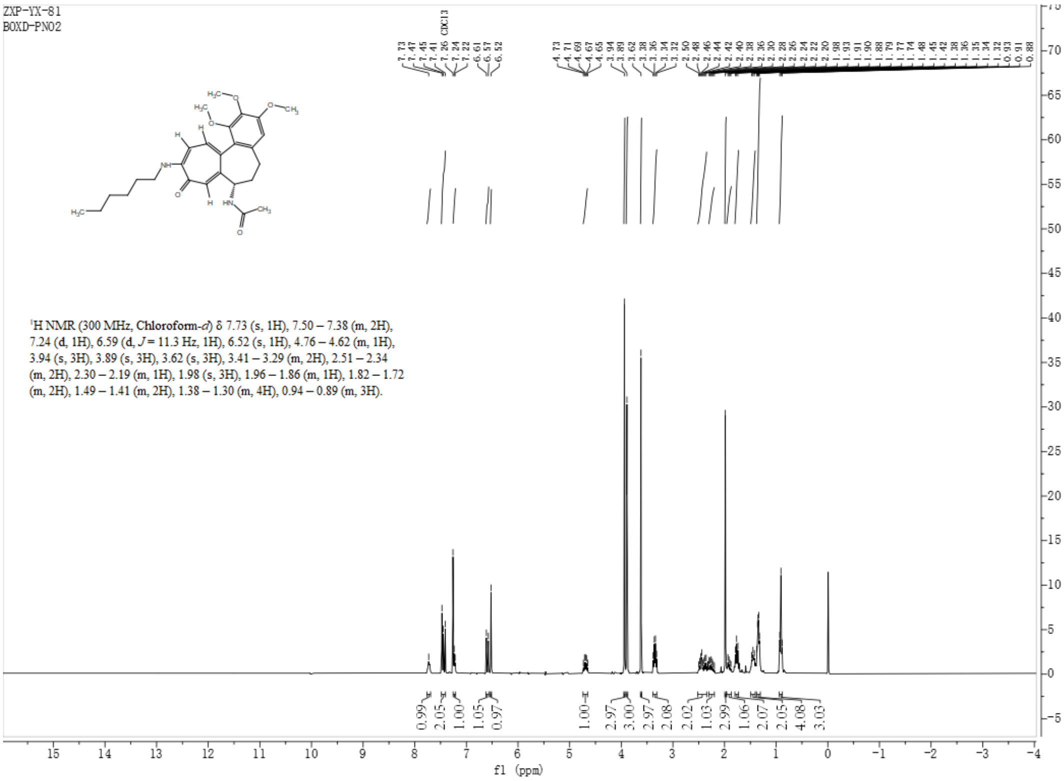

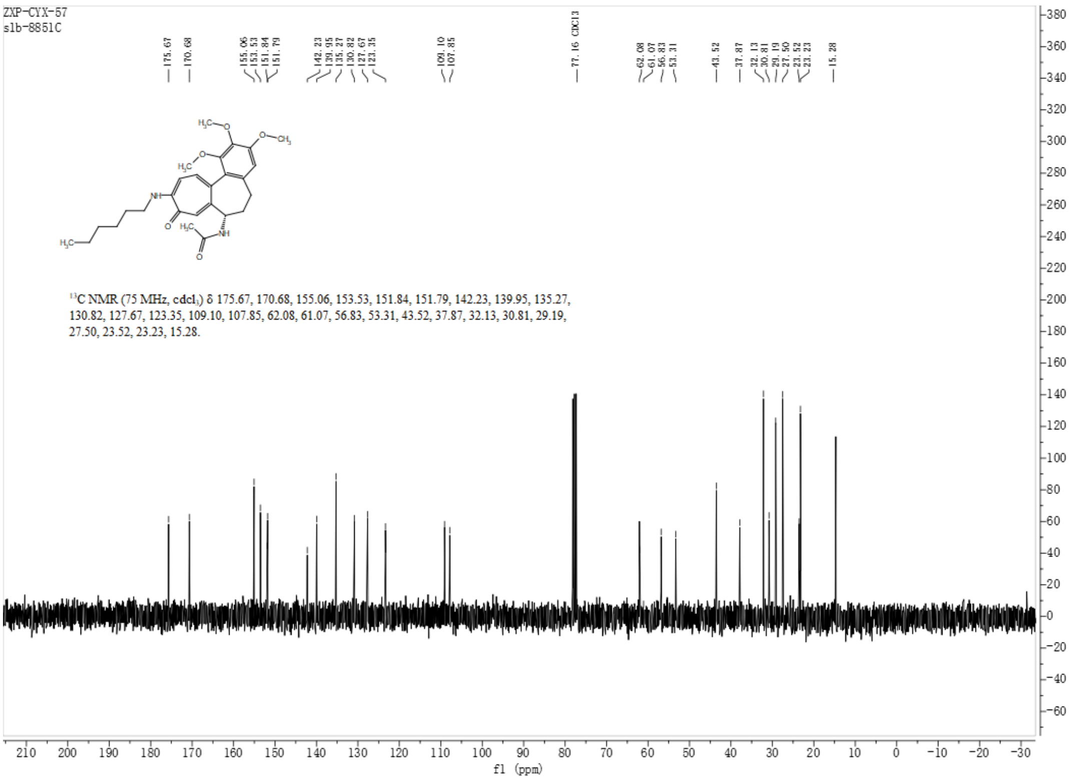
ZXP-CYX-57
slb-8851C
175.67
170.68
155.06
153.53
151.84
151.79
142.23
139.95
135.27
130.82
127.67
123.35
109.10
107.85
77.16 CDCl3
62.08
61.07
56.83
53.31
43.52
37.87
32.13
30.81
29.19
27.50
23.52
23.23
15.28
^{13}C NMR (75 MHz, cdcl$_3$) δ 175.67, 170.68, 155.06, 153.53, 151.84, 151.79, 142.23, 139.95, 135.27, 130.82, 127.67, 123.35, 109.10, 107.85, 62.08, 61.07, 56.83, 53.31, 43.52, 37.87, 32.13, 30.81, 29.19, 27.50, 23.52, 23.23, 15.28.
f1 (ppm)

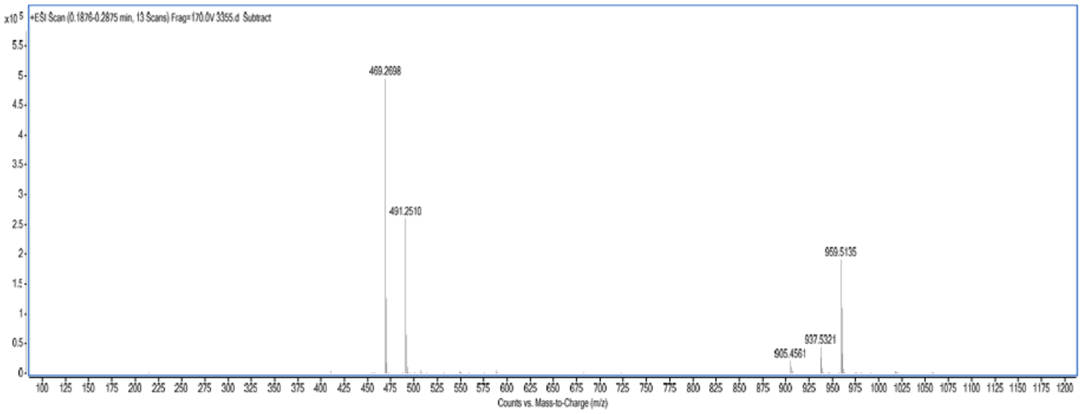
x10 5
+ESI Scan (0.1876-0.2875 min, 13 Scans) Frag=170.0V 3355.d Subtract
469.2698
491.2510
959.5135
937.5321
905.4561
Counts vs. Mass-to-Charge (m/z)

Figure S9. ^{1}H-NMR, ^{13}C-NMR and HRMS data of compound 9b

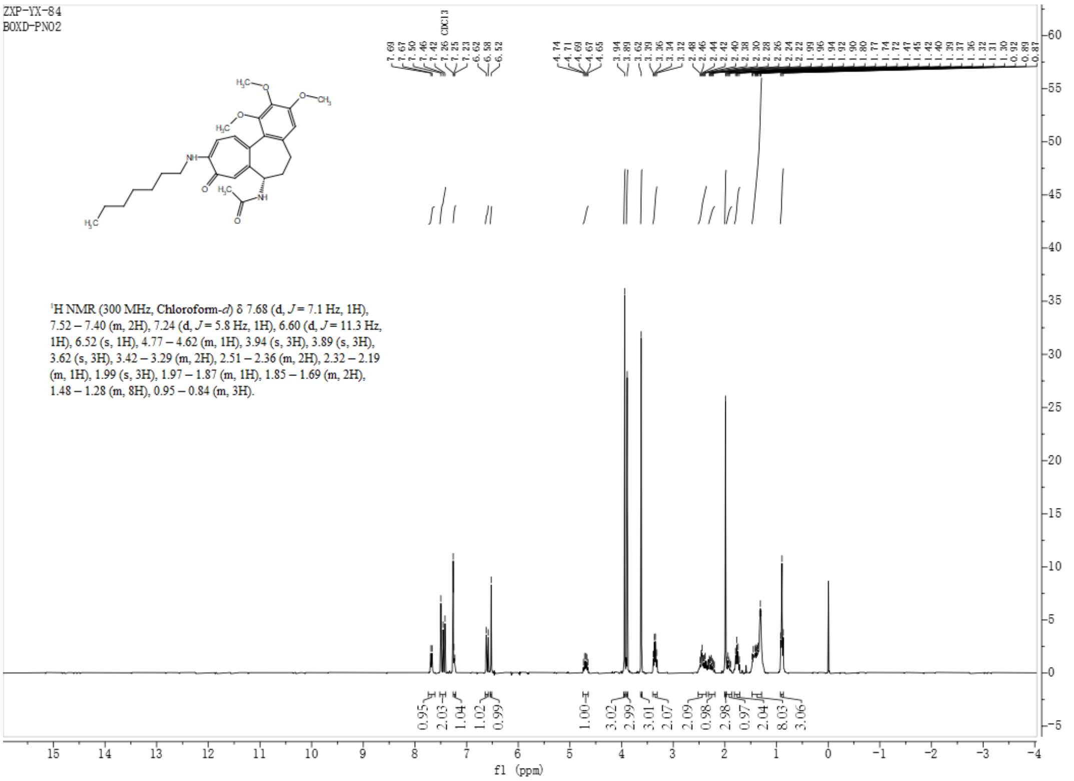

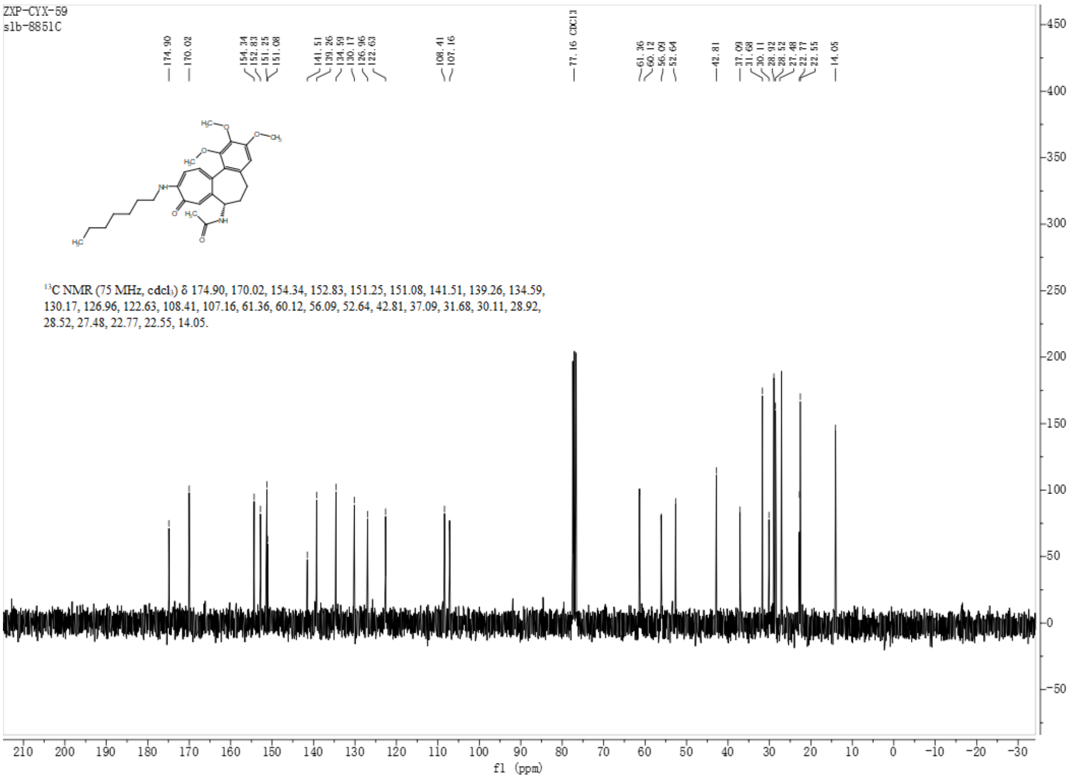

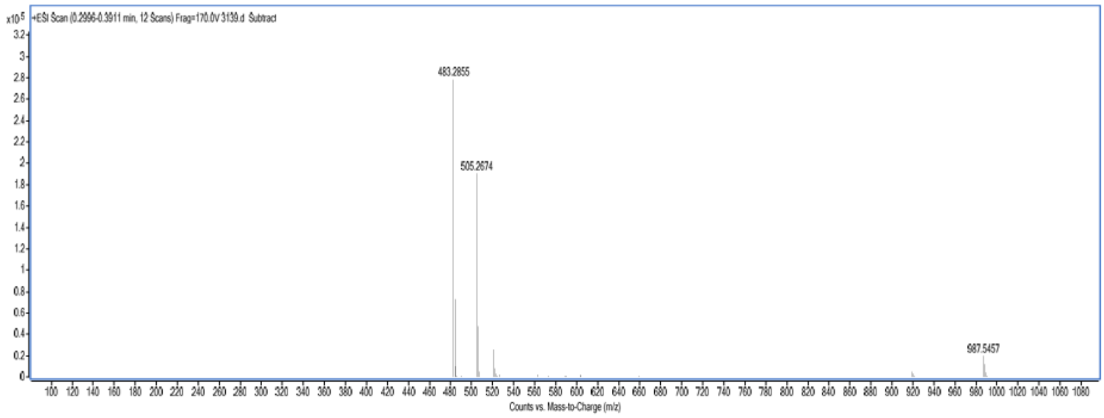

Figure S10. ^{1}H-NMR, ^{13}C-NMR and HRMS data of compound 10b

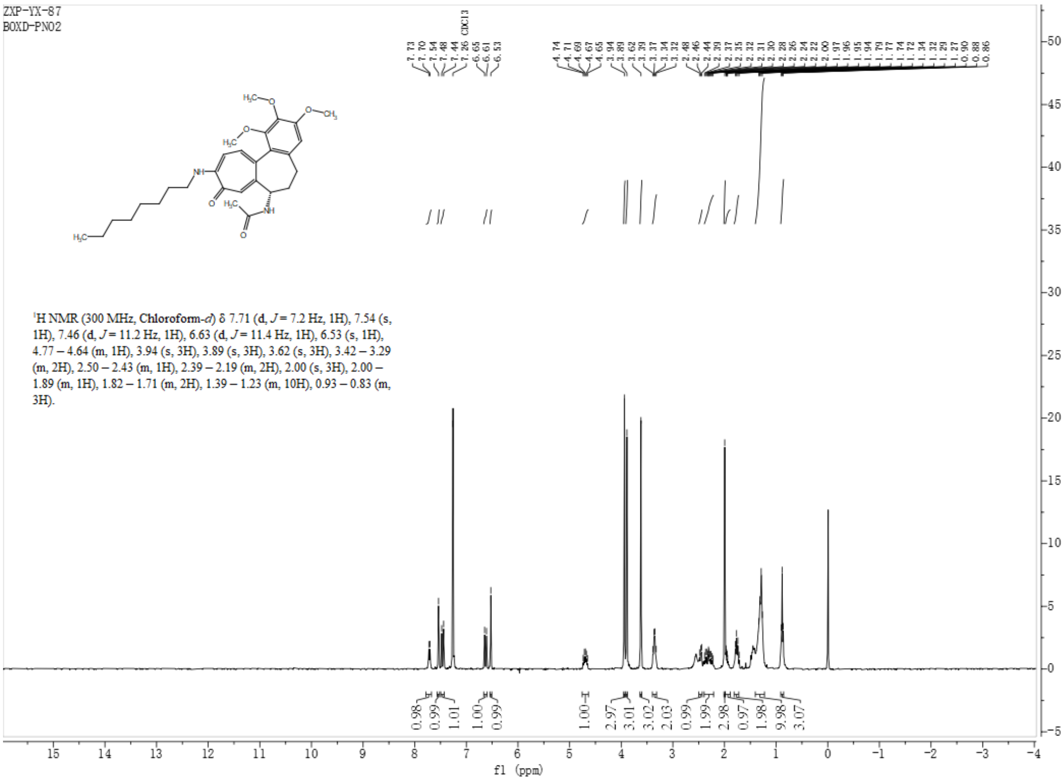

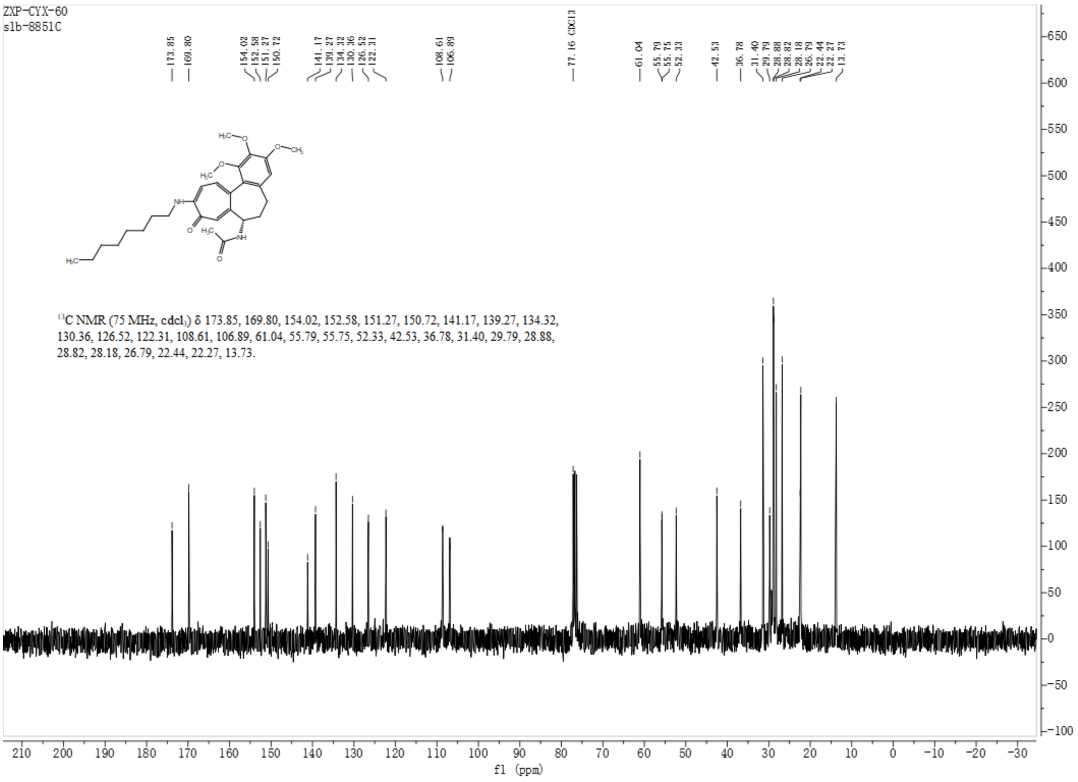
ZXP-CYX-60
slb-8851C
173.85
169.80
154.02
152.58
151.27
150.72
141.17
139.27
134.32
130.36
126.52
122.31
108.61
106.89
77.16 CDCl3
61.04
55.79
55.75
52.33
42.53
36.78
31.40
29.79
28.88
28.82
28.18
26.79
22.44
22.27
13.73
13C NMR (75 MHz, cdcl3) δ 173.85, 169.80, 154.02, 152.58, 151.27, 150.72, 141.17, 139.27, 134.32, 130.36, 126.52, 122.31, 108.61, 106.89, 61.04, 55.79, 55.75, 52.33, 42.53, 36.78, 31.40, 29.79, 28.88, 28.82, 28.18, 26.79, 22.44, 22.27, 13.73.
f1 (ppm)

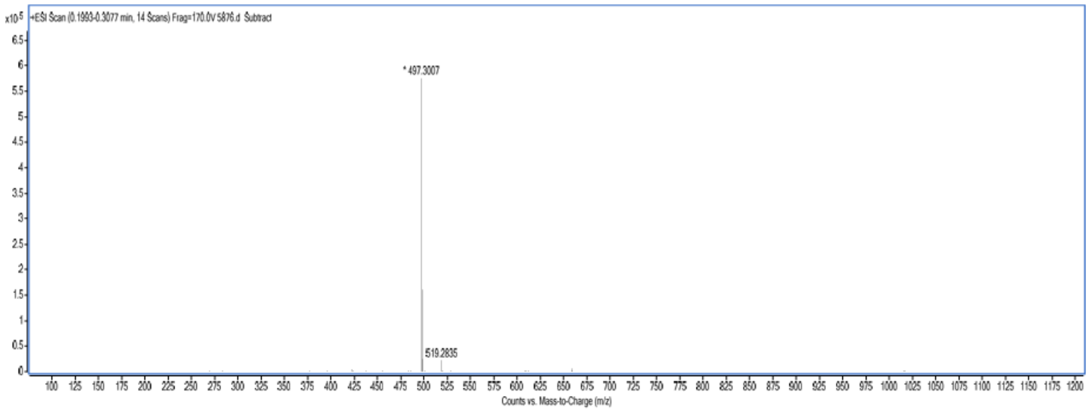
+ESI Scan (0.1993-0.3077 min, 14 Scans) Frag=170.0V 5876.d Subtract
x10^5
* 497.3007
519.2835
Counts vs. Mass-to-Charge (m/z)

Figure S11. ^{1}H-NMR, ^{13}C-NMR and HRMS data of compound 11b

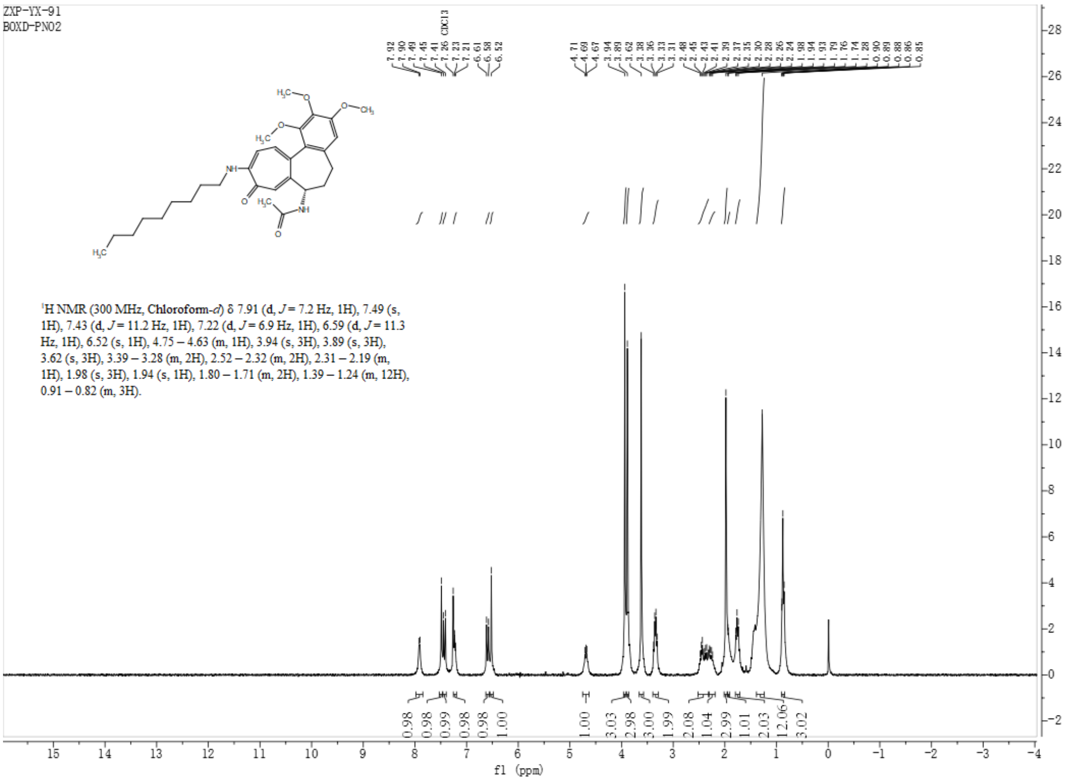

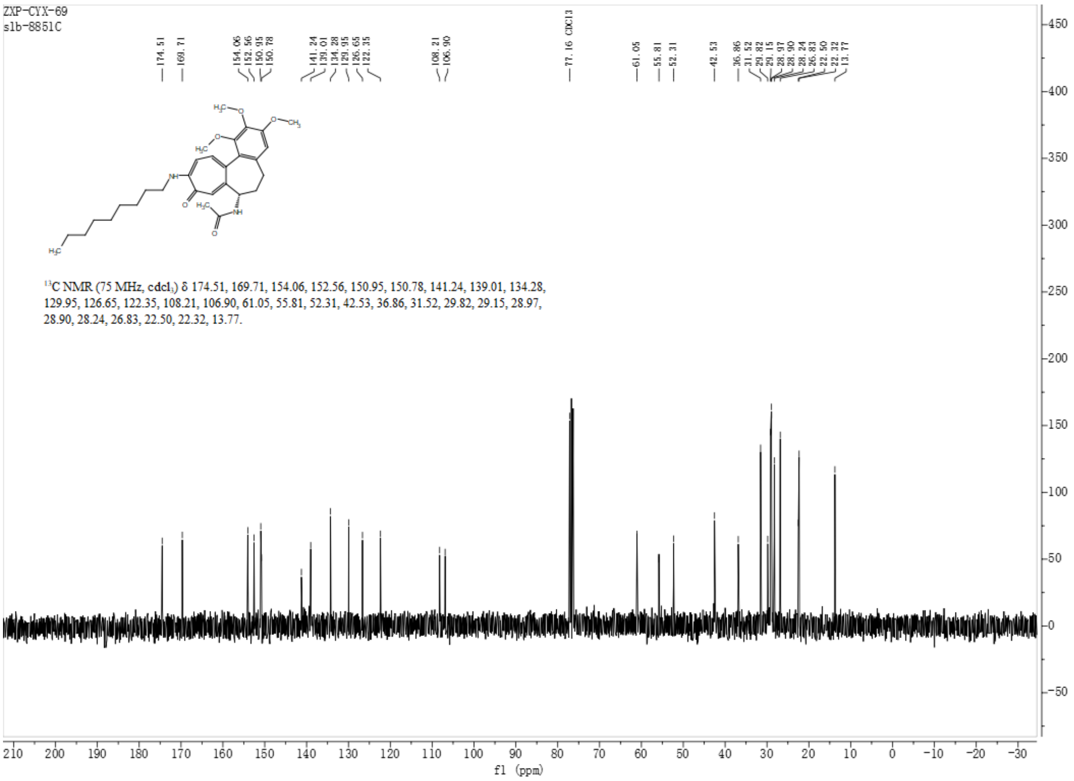

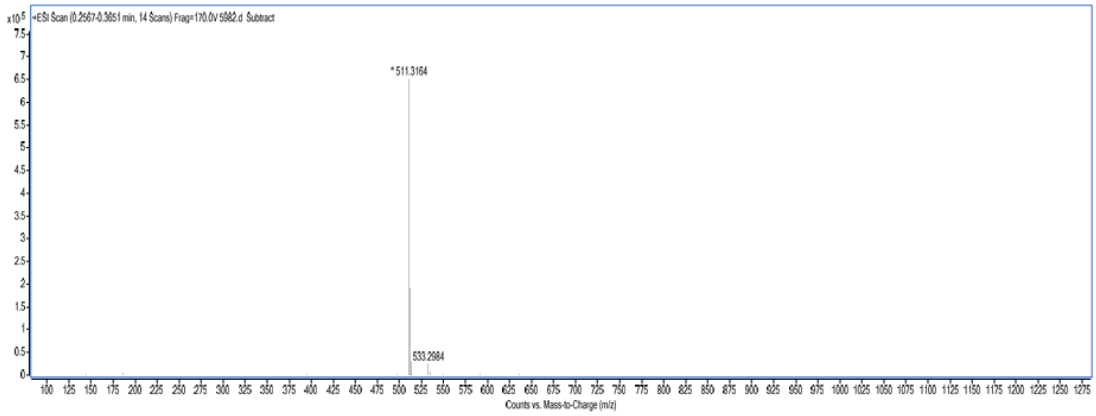

Figure S12. ^{1}H-NMR, ^{13}C-NMR and HRMS data of compound 12b

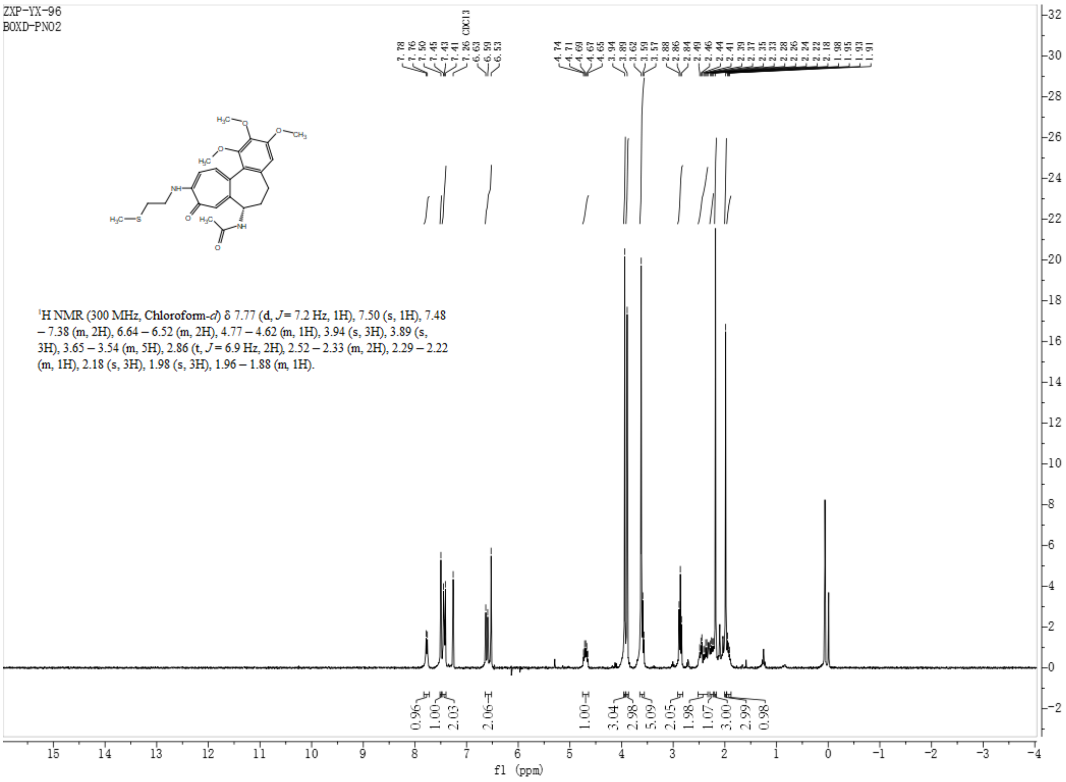

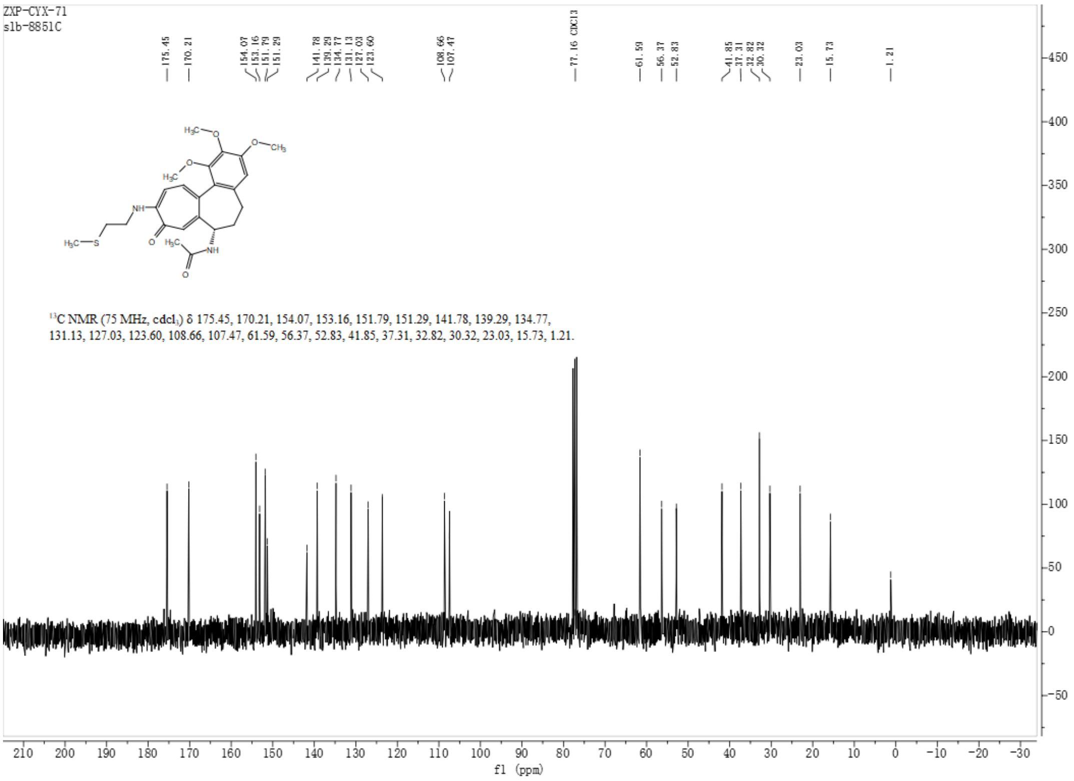
ZXP-CYX-71
slb-8851C
13C NMR (75 MHz, cdcl3) δ 175.45, 170.21, 154.07, 153.16, 151.79, 151.29, 141.78, 139.29, 134.77, 131.13, 127.03, 123.60, 108.66, 107.47, 61.59, 56.37, 52.83, 41.85, 37.31, 32.82, 30.32, 23.03, 15.73, 1.21.
77.16 CDCl3
f1 (ppm)

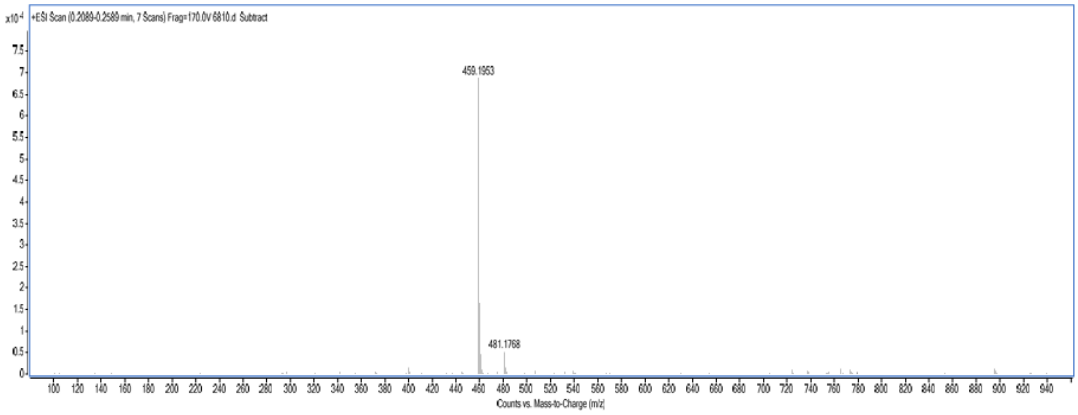
+ESI Scan (0.2089-0.2589 min, 7 Scans) Frag=170.0V 6810.d Subtract
459.1953
481.1768
Counts vs. Mass-to-Charge (m/z)

Figure S13. ^{1}H-NMR, ^{13}C-NMR and HRMS data of compound 13b

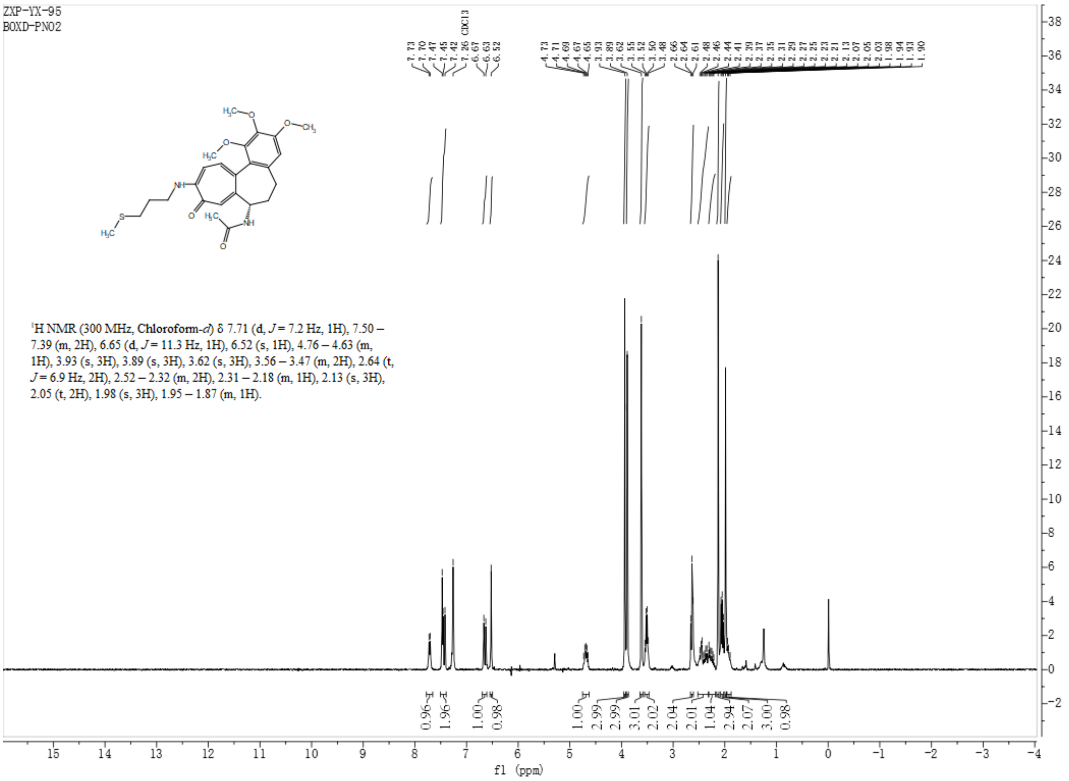

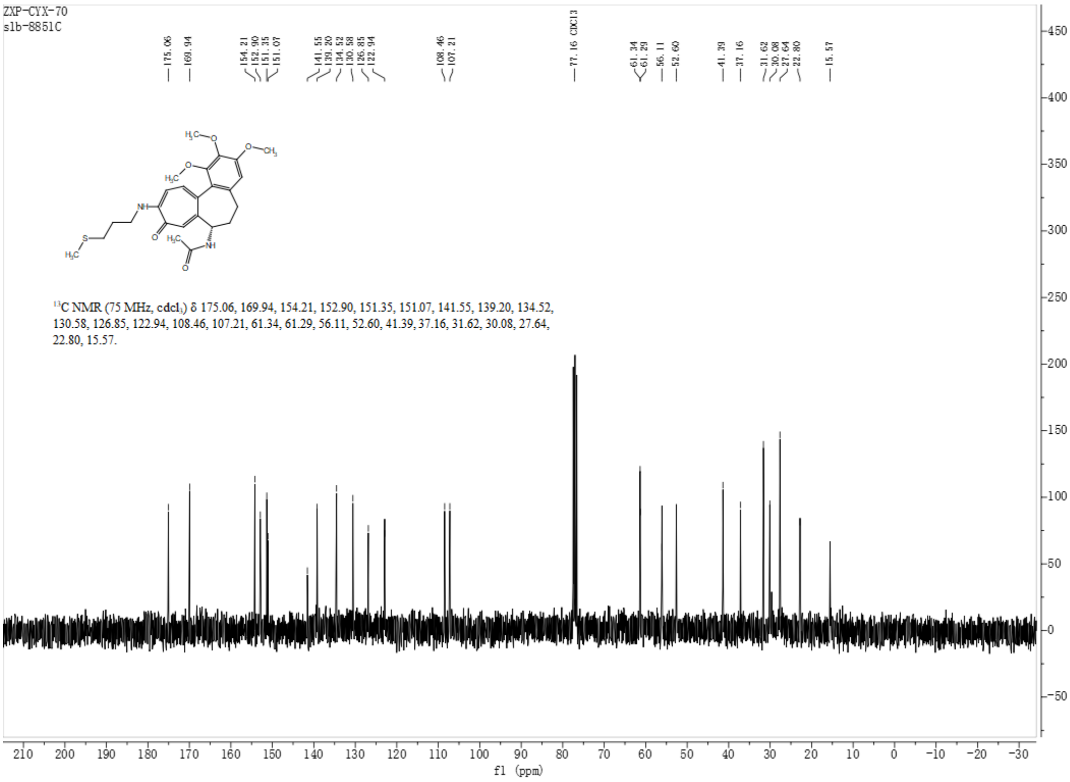

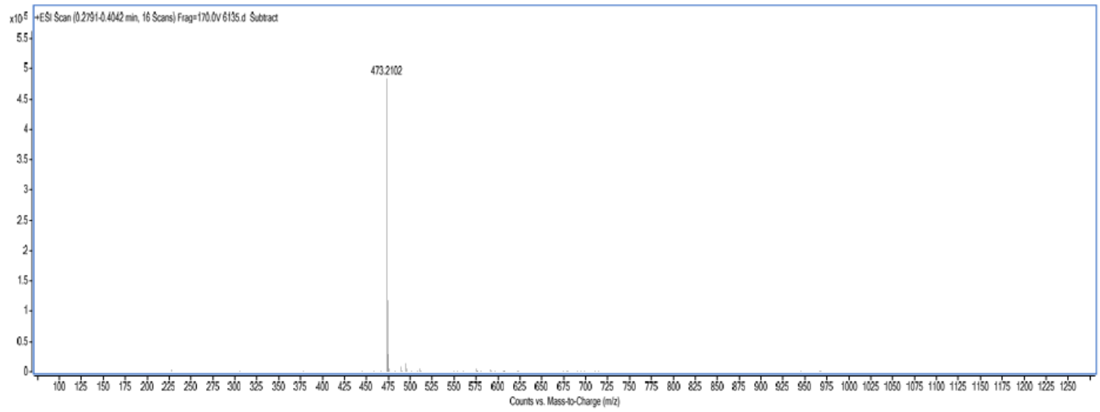

Figure S14. ^{1}H-NMR, ^{13}C-NMR and HRMS data of compound 14b

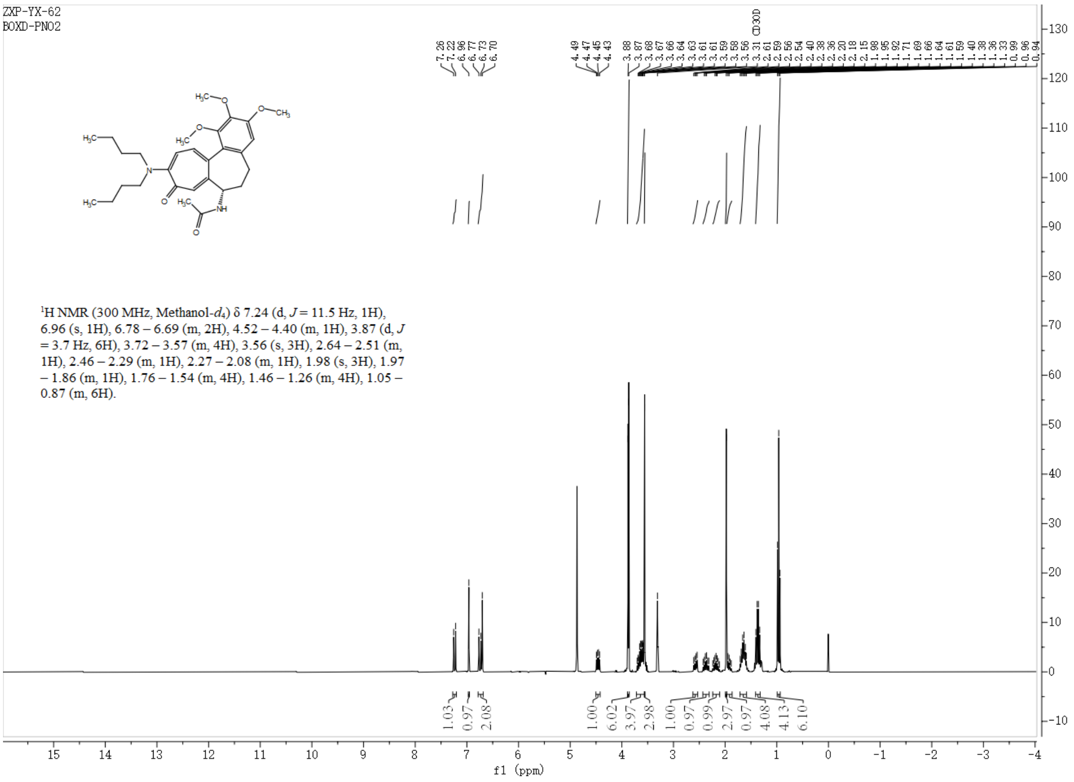

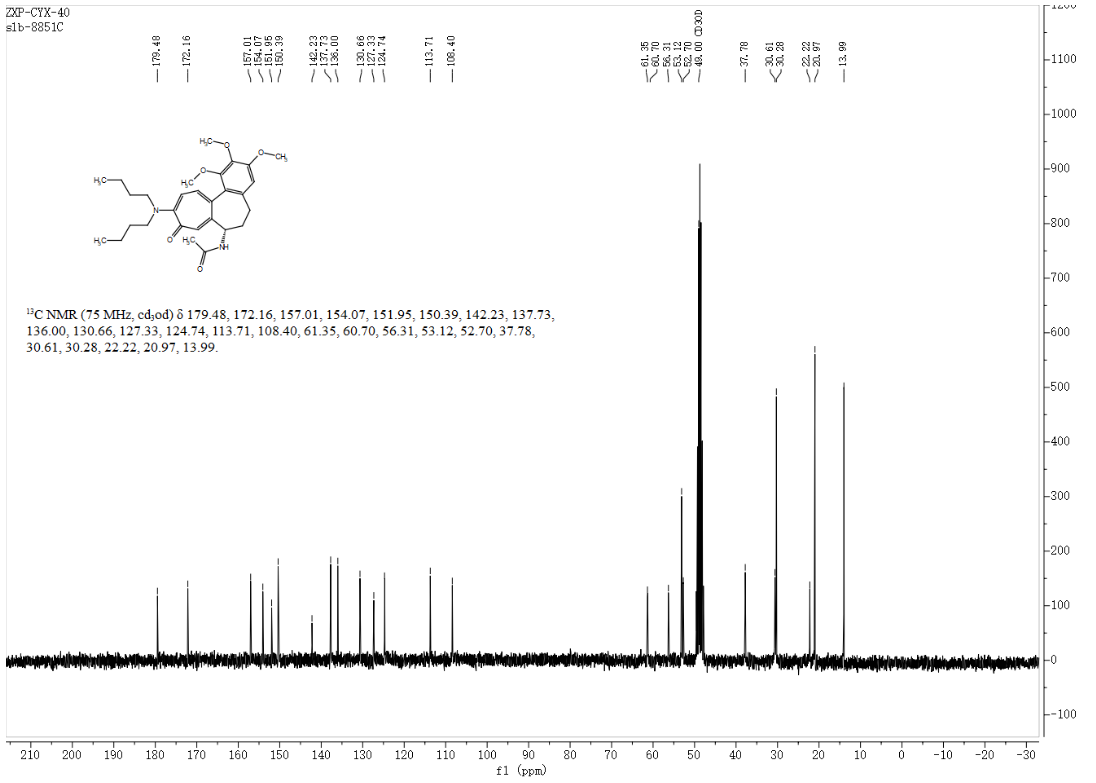
ZXP-CYX-40
slb-8851C
13C NMR (75 MHz, cd3od) δ 179.48, 172.16, 157.01, 154.07, 151.95, 150.39, 142.23, 137.73, 136.00, 130.66, 127.33, 124.74, 113.71, 108.40, 61.35, 60.70, 56.31, 53.12, 52.70, 37.78, 30.61, 30.28, 22.22, 20.97, 13.99.
f1 (ppm)

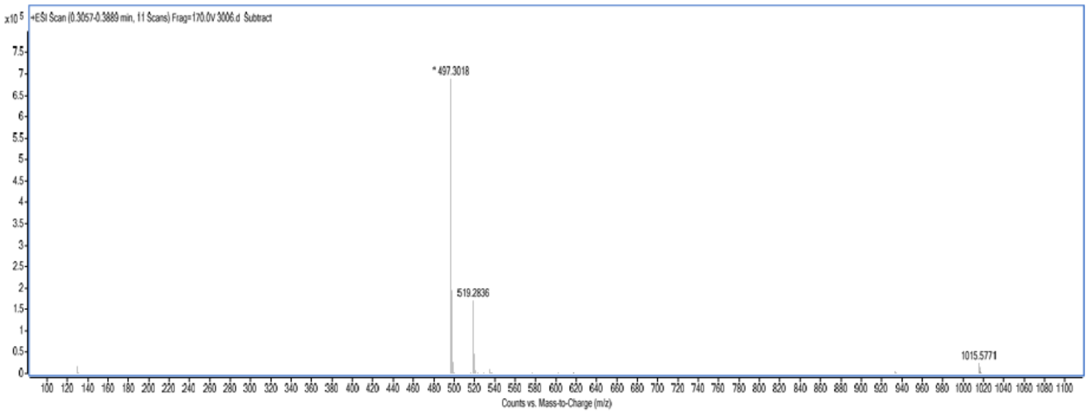
+ESI Scan (0.3057-0.3889 min, 11 Scans) Frag=170.0V 3006.d Subtract
x10 5
* 497.3018
519.2836
1015.5771
Counts vs. Mass-to-Charge (m/z)

Figure S15. ^{1}H-NMR, ^{13}C-NMR and HRMS data of compound 15b

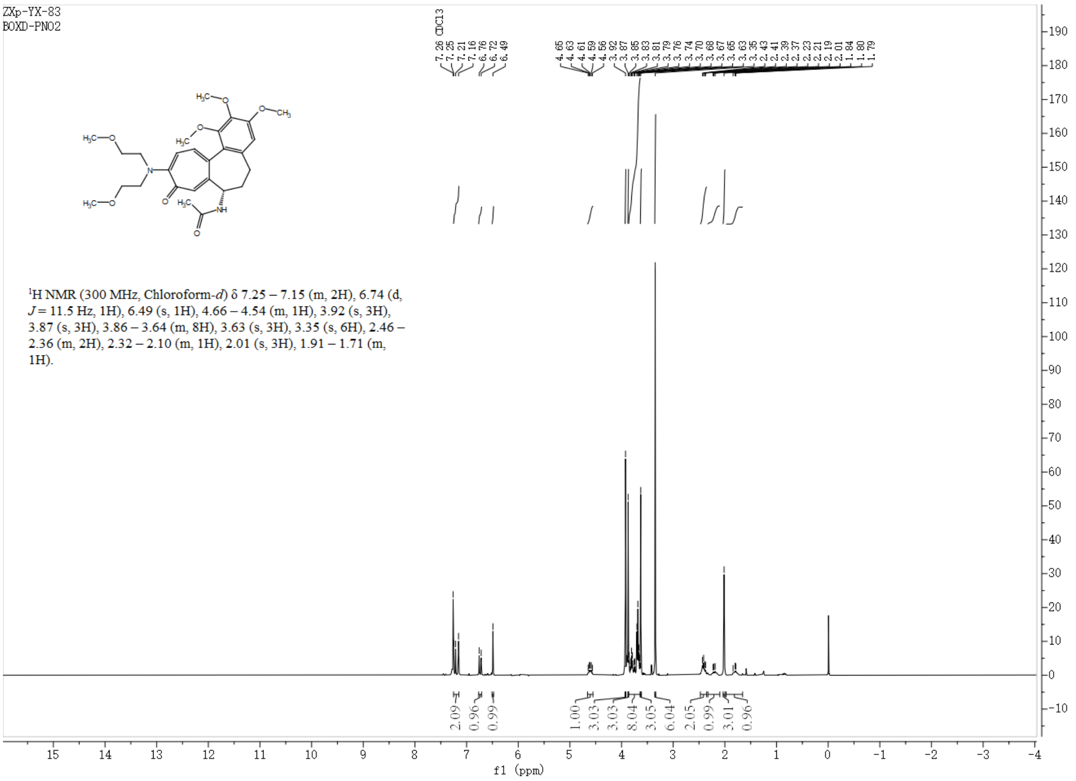

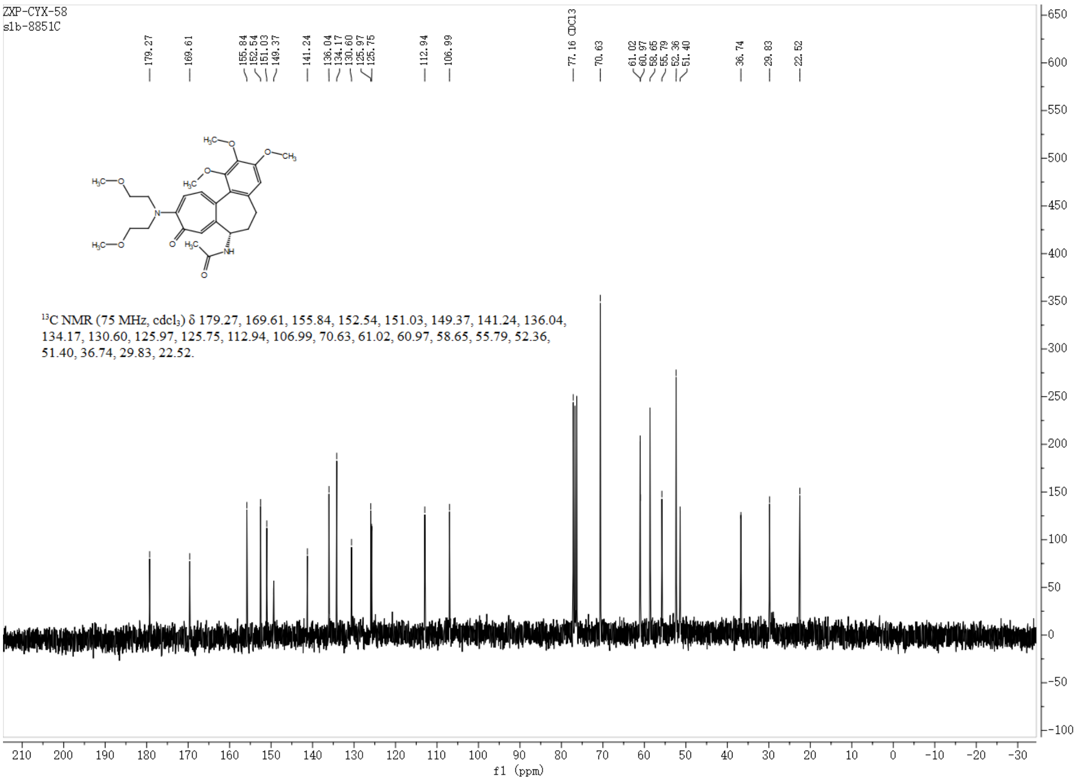

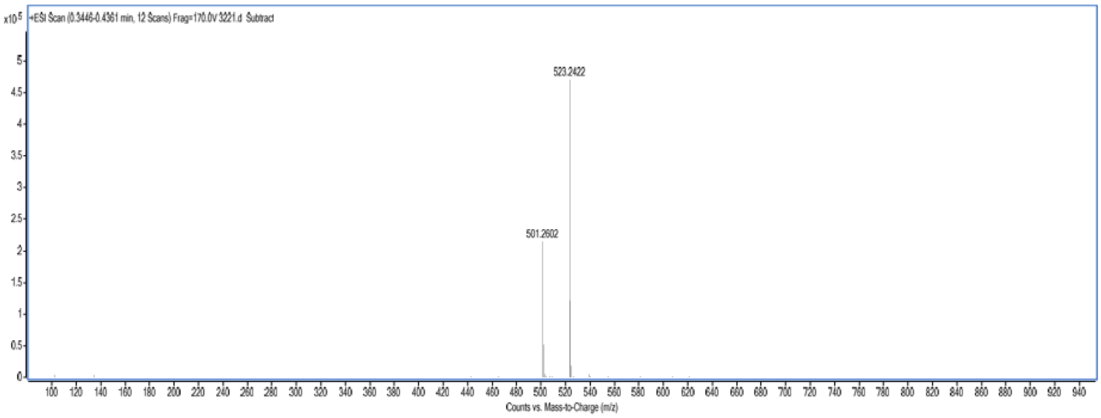

Figure S16. ^{1}H-NMR, ^{13}C-NMR and HRMS data of compound 16b

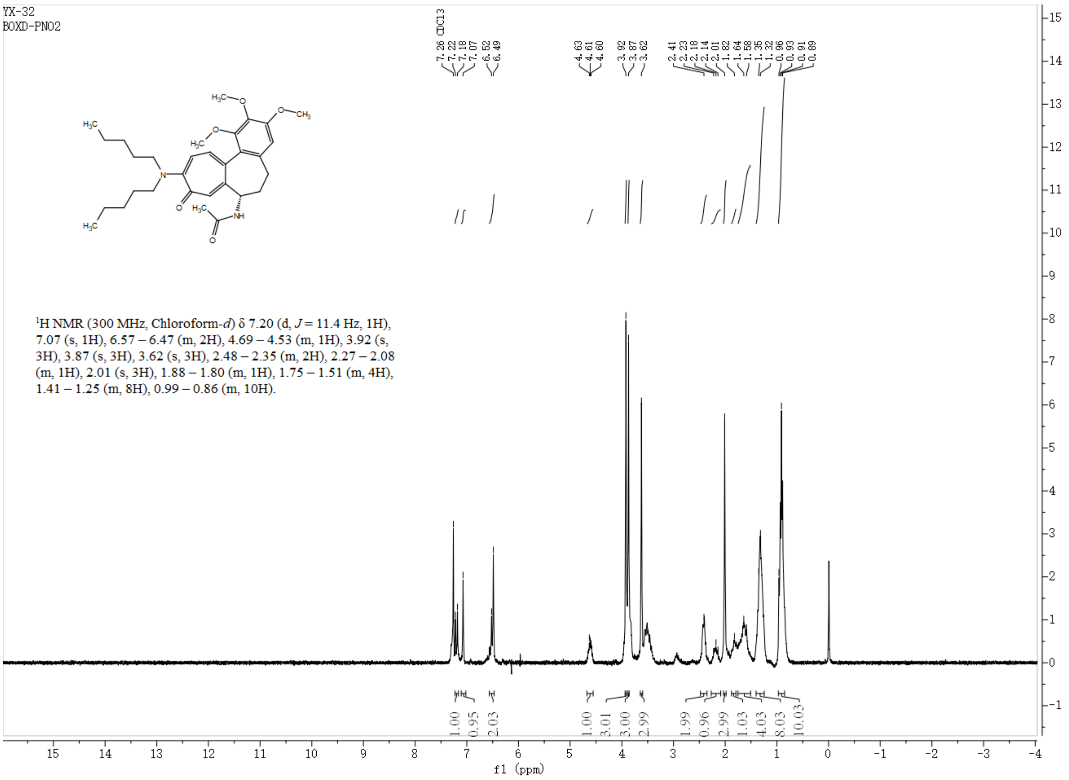

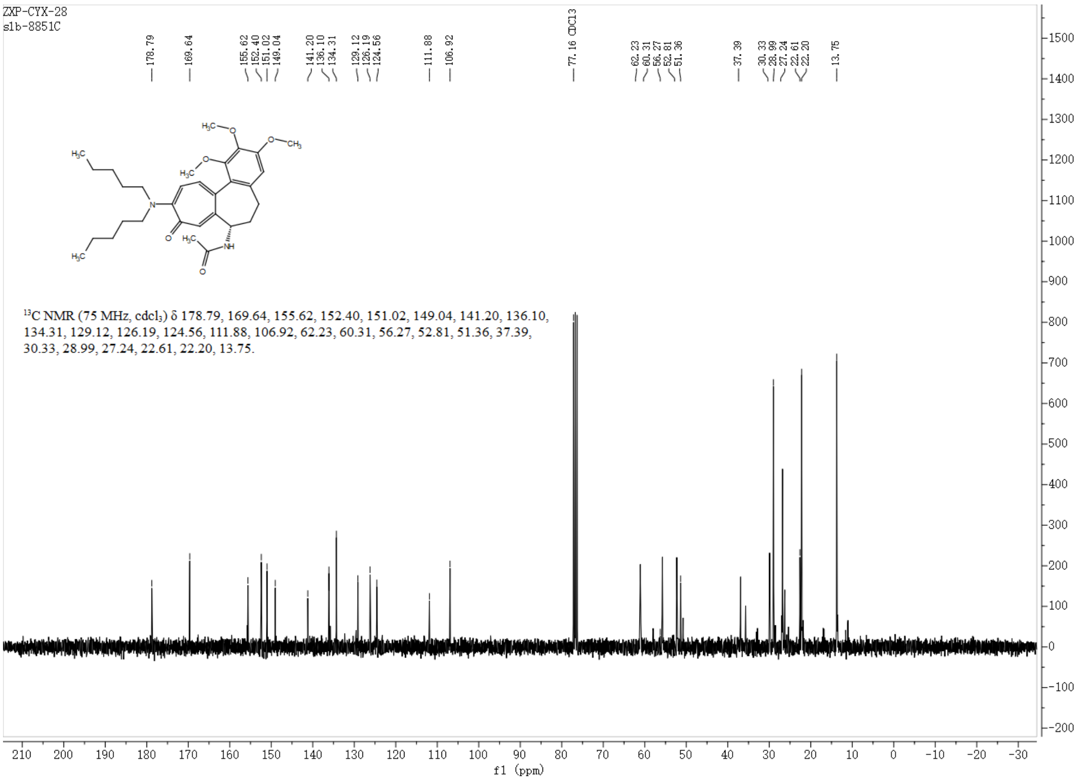
ZXP-CYX-28
slb-8851C
178.79
169.64
155.62
152.40
151.02
149.04
141.20
136.10
134.31
129.12
126.19
124.56
111.88
106.92
77.16 CDCl3
62.23
60.31
56.27
52.81
51.36
37.39
30.33
28.99
27.24
22.61
22.20
13.75
^{13}C NMR (75 MHz, cdcl$_3$) δ 178.79, 169.64, 155.62, 152.40, 151.02, 149.04, 141.20, 136.10, 134.31, 129.12, 126.19, 124.56, 111.88, 106.92, 62.23, 60.31, 56.27, 52.81, 51.36, 37.39, 30.33, 28.99, 27.24, 22.61, 22.20, 13.75.
f1 (ppm)

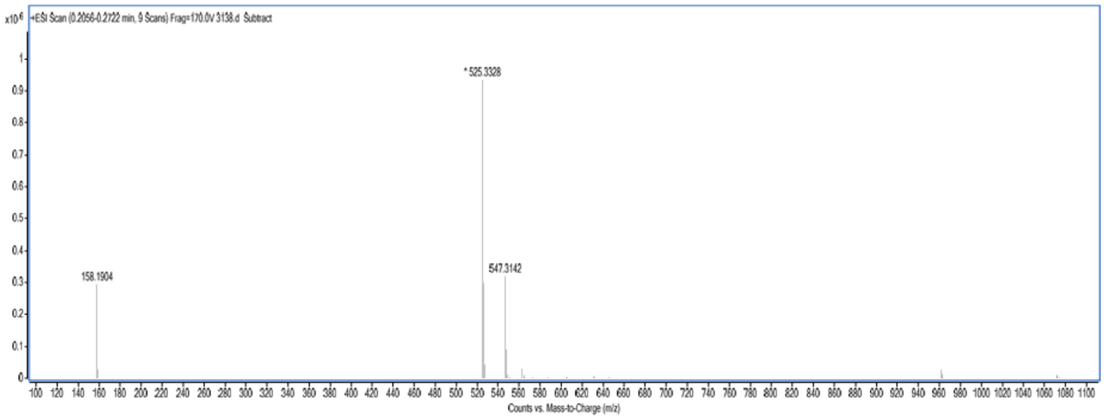
x10^6
+ESI Scan (0.2056-0.2722 min, 9 Scans) Frag=170.0V 3138.d Subtract
* 525.3328
547.3142
158.1904
Counts vs. Mass-to-Charge (m/z)

Figure S17. ^{1}H-NMR, ^{13}C-NMR and HRMS data of compound 17b

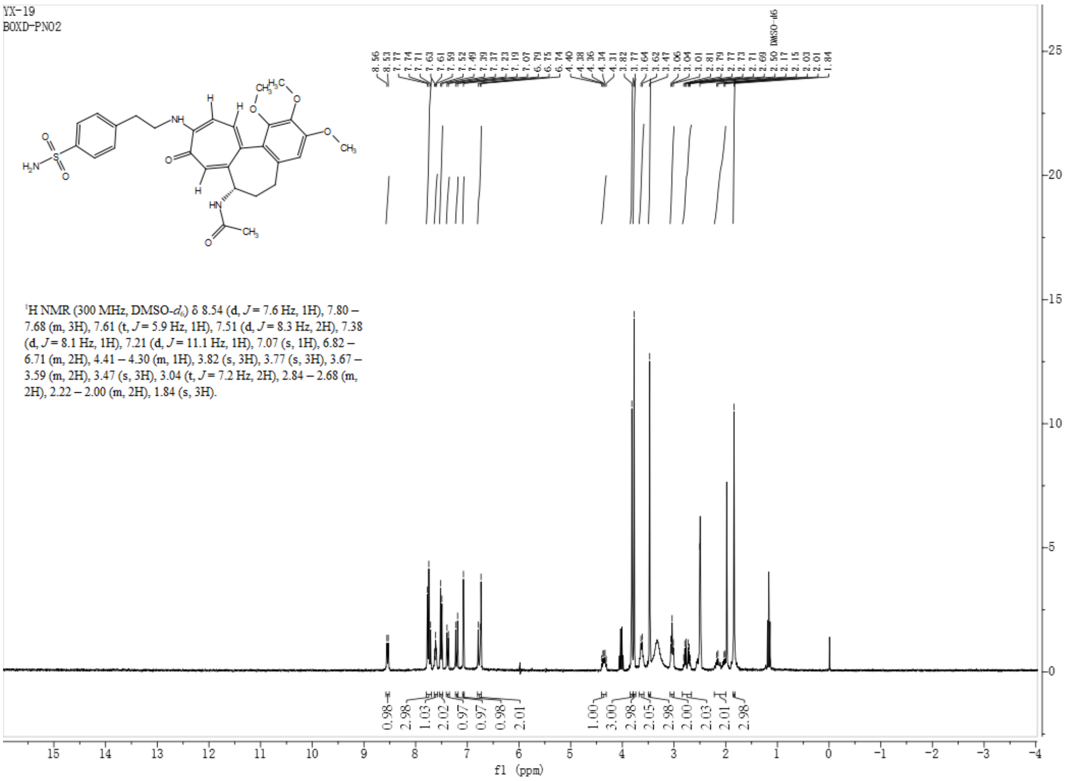

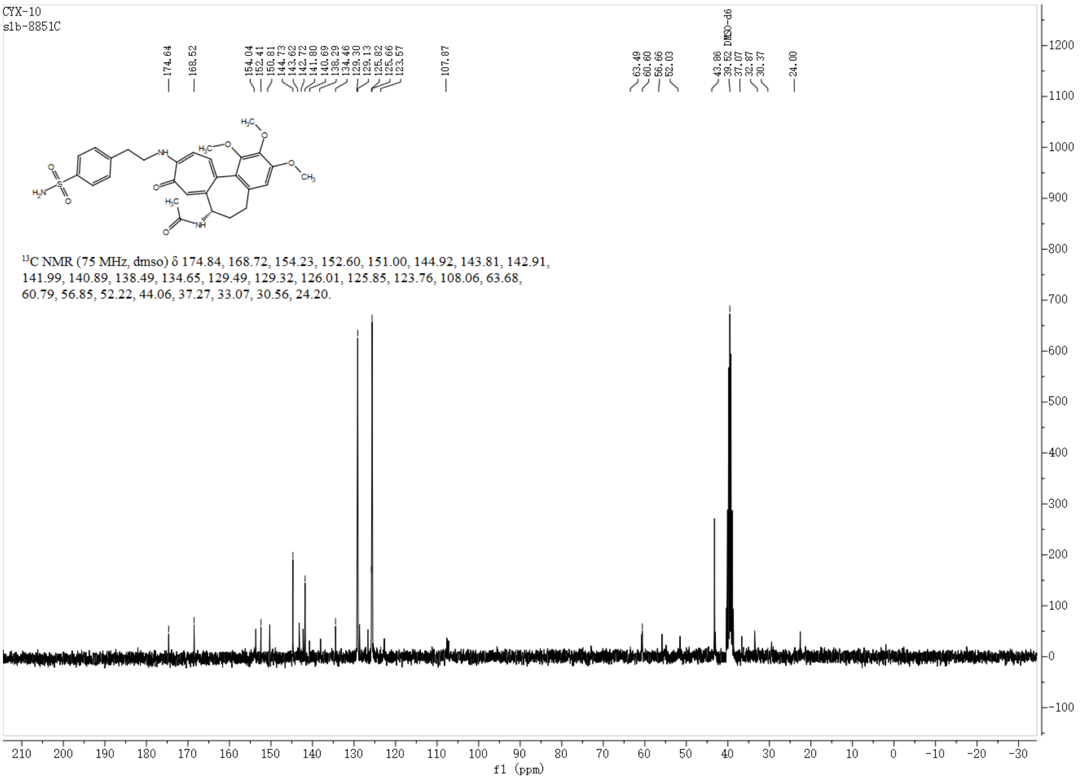

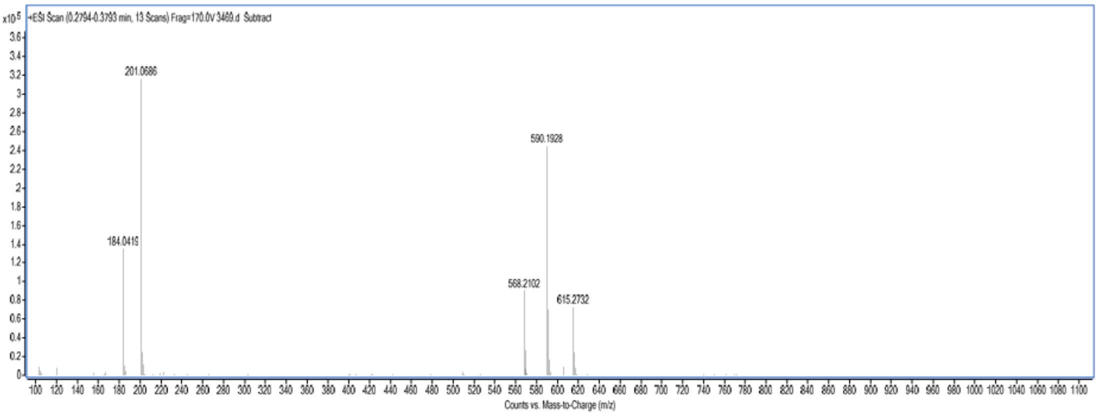

Figure S18. ^{1}H-NMR, ^{13}C-NMR and HRMS data of compound 18b

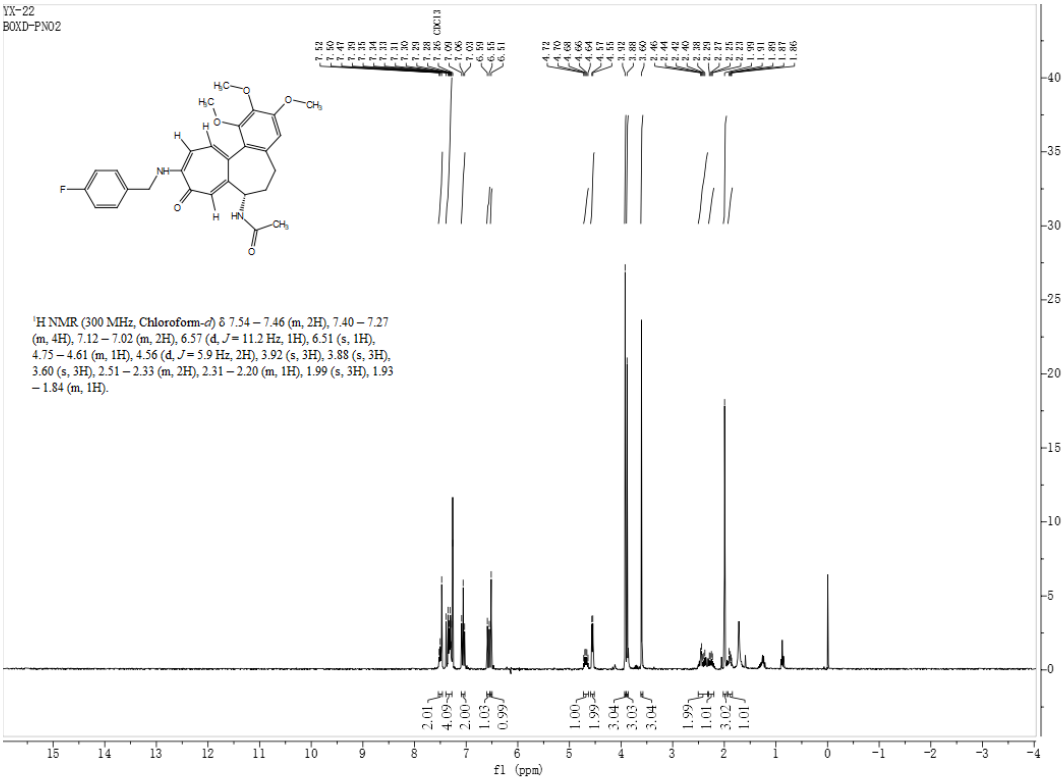

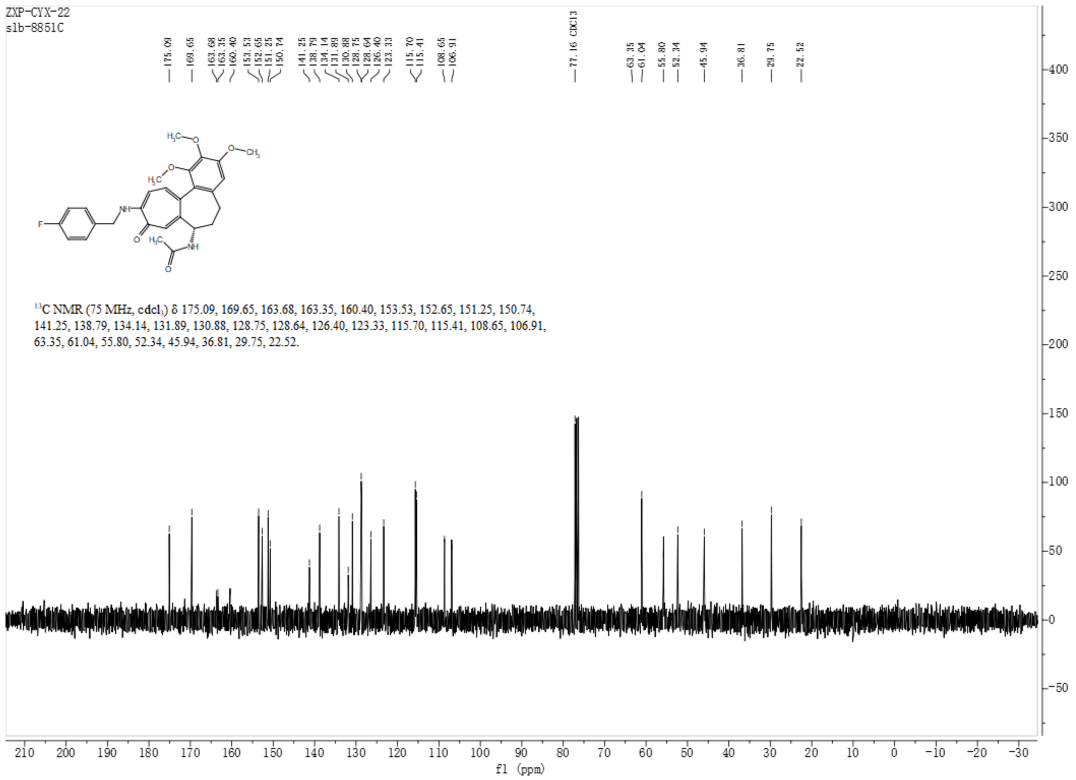
ZXP-CYX-22
slb-8851C
175.09
169.65
163.68
163.35
160.40
153.53
152.65
151.25
150.74
141.25
138.79
134.14
131.89
130.88
128.75
128.64
126.40
123.33
115.70
115.41
108.65
106.91
77.16 CDCl3
63.35
61.04
55.80
52.34
45.94
36.81
29.75
22.52
13C NMR (75 MHz, cdcl3) δ 175.09, 169.65, 163.68, 163.35, 160.40, 153.53, 152.65, 151.25, 150.74, 141.25, 138.79, 134.14, 131.89, 130.88, 128.75, 128.64, 126.40, 123.33, 115.70, 115.41, 108.65, 106.91, 63.35, 61.04, 55.80, 52.34, 45.94, 36.81, 29.75, 22.52.
f1 (ppm)

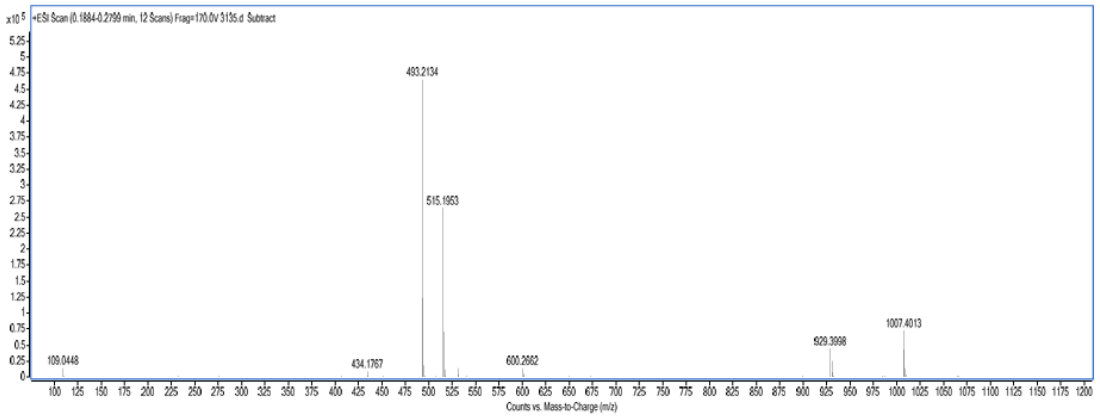
x10 5
+ESI Scan (0.1884-0.2799 min, 12 Scans) Frag=170.0V 3135.d Subtract
493.2134
515.1953
1007.4013
929.3998
600.2662
434.1767
109.0448
Counts vs. Mass-to-Charge (m/z)

Figure S19. ^{1}H-NMR, ^{13}C-NMR and HRMS data of compound 19b

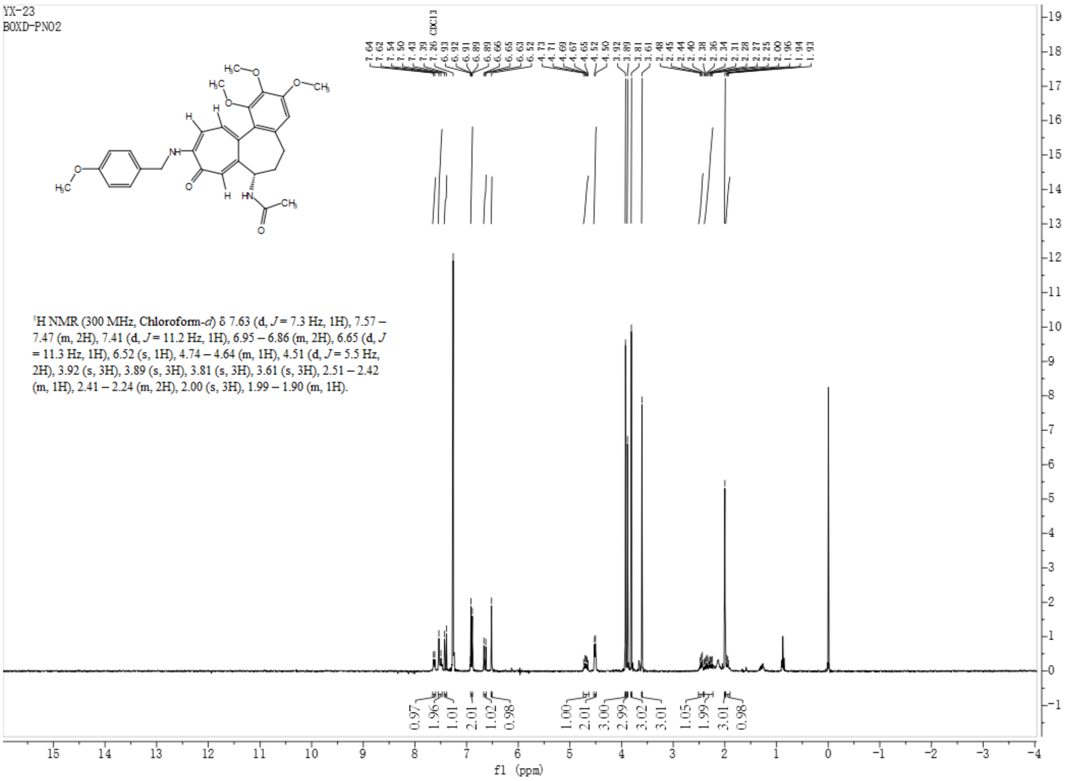

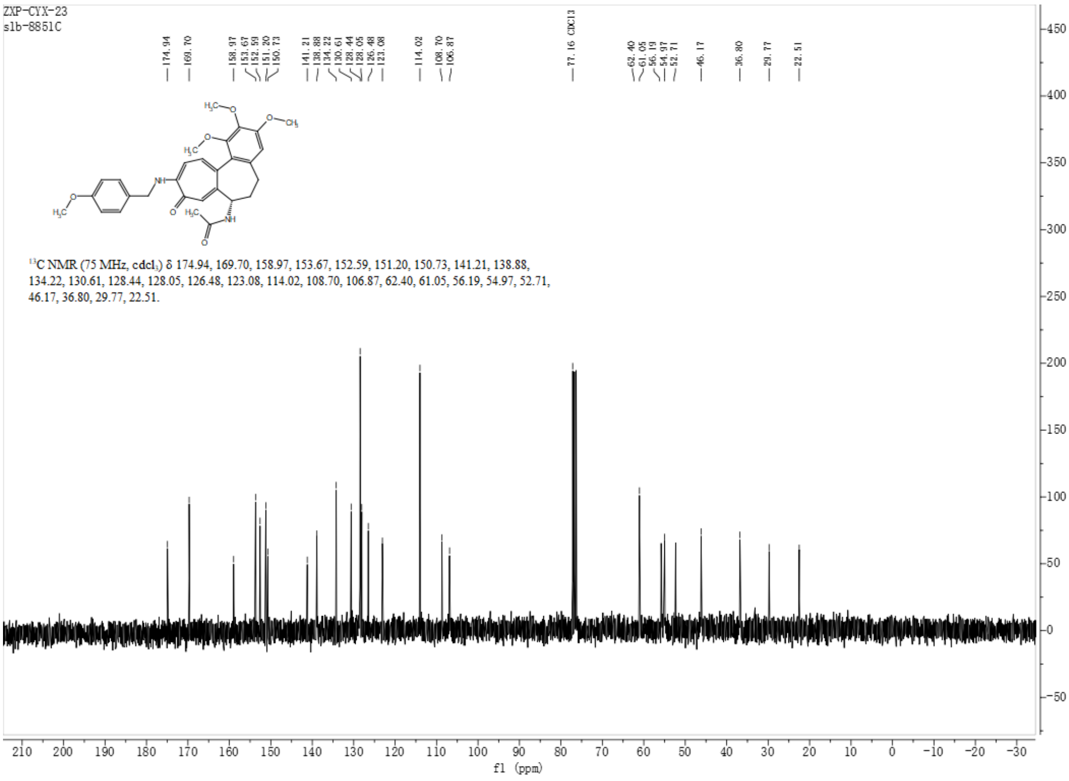

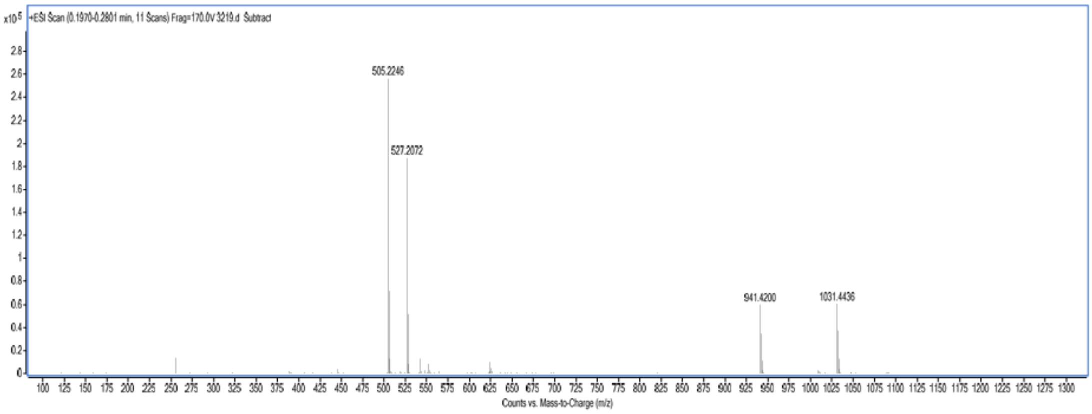

Figure S20. ^{1}H-NMR, ^{13}C-NMR and HRMS data of compound 20b

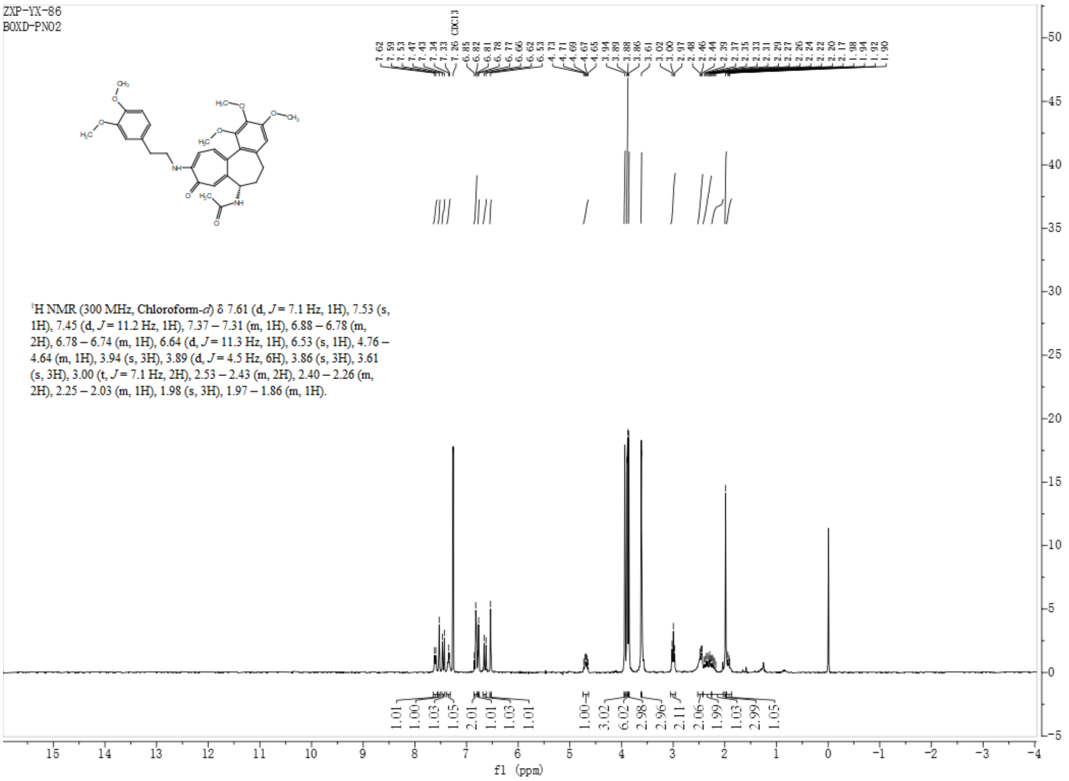

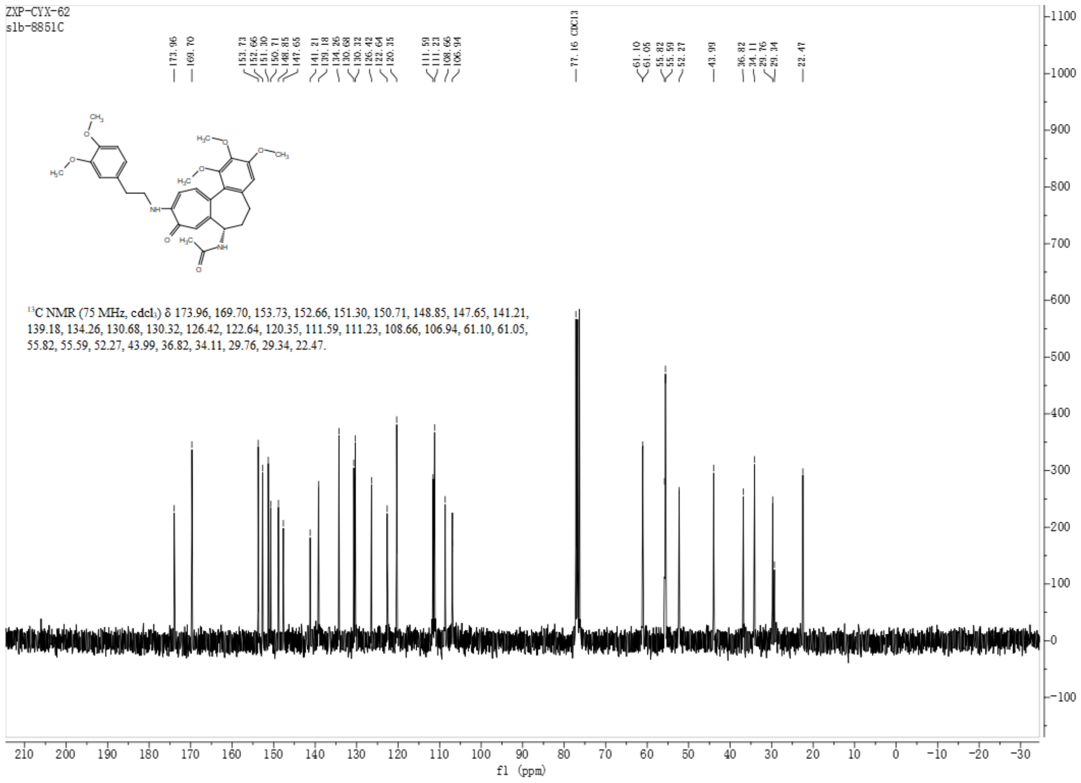
ZXP-CYX-62
slb-8851C
77.16 CDCl3
^{13}C NMR (75 MHz, cdcl$_3$) δ 173.96, 169.70, 153.73, 152.66, 151.30, 150.71, 148.85, 147.65, 141.21, 139.18, 134.26, 130.68, 130.32, 126.42, 122.64, 120.35, 111.59, 111.23, 108.66, 106.94, 61.10, 61.05, 55.82, 55.59, 52.27, 43.99, 36.82, 34.11, 29.76, 29.34, 22.47.
f1 (ppm)

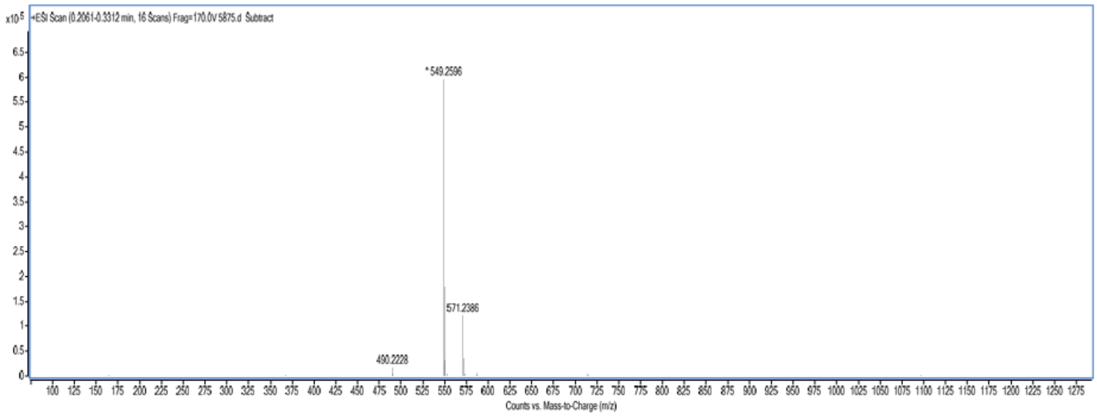
+ESI Scan (0.2061-0.3312 min, 16 Scans) Frag=170.0V 5875.d Subtract
x10^5
*549.2596
571.2386
490.2228
Counts vs. Mass-to-Charge (m/z)

Figure S21. ^{1}H-NMR, ^{13}C-NMR and HRMS data of compound 21b

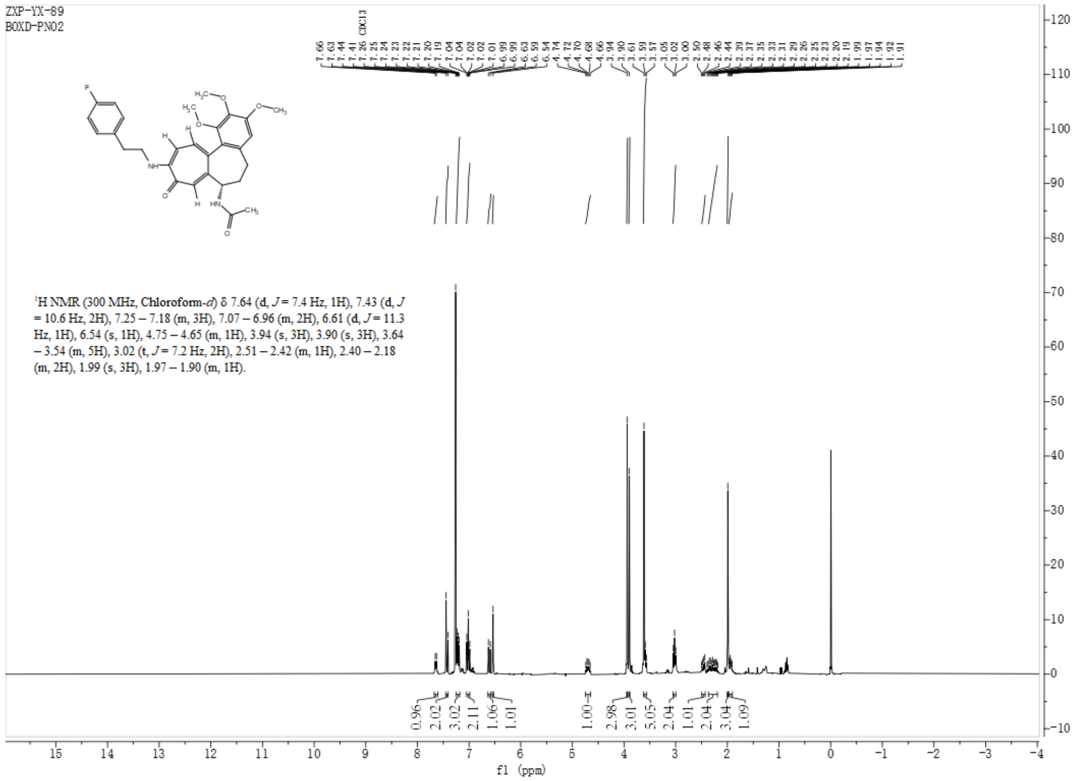

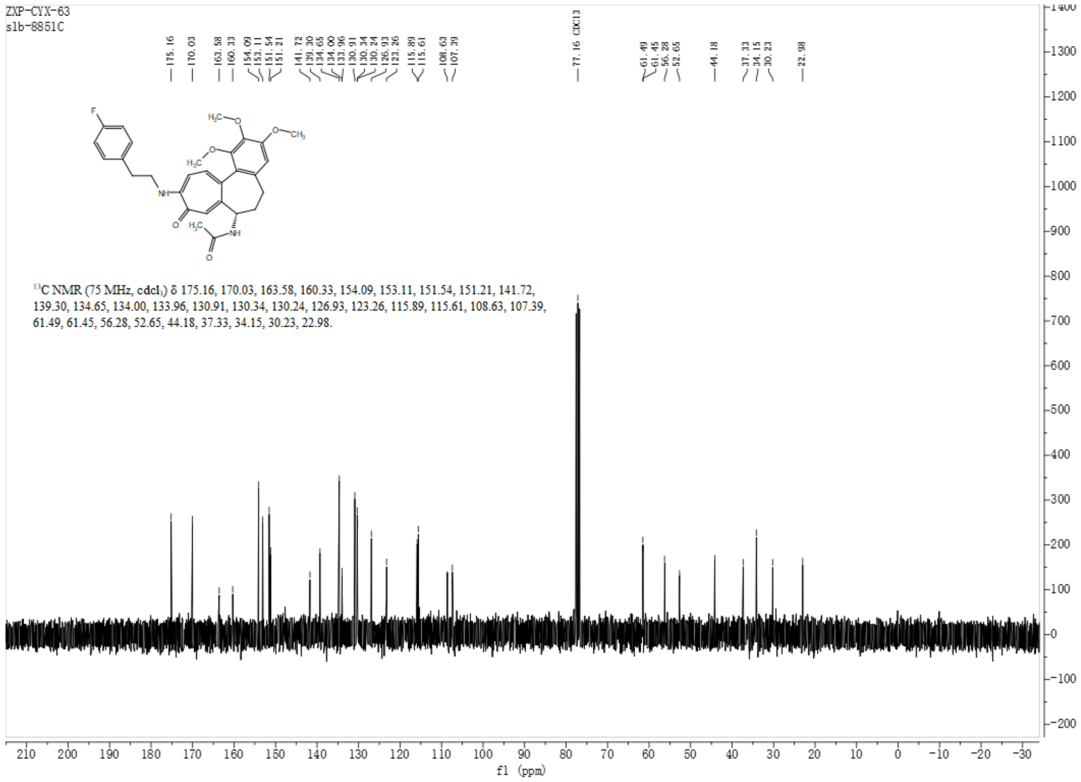

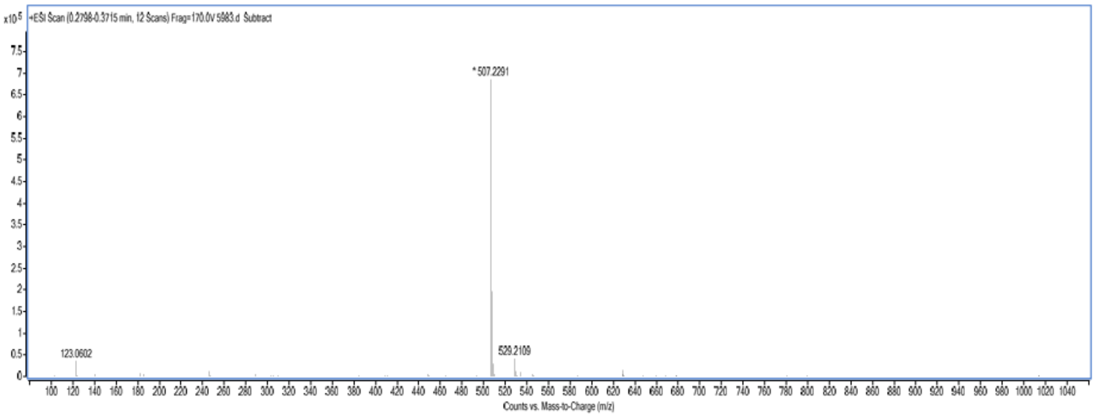

Figure S22. ^{1}H-NMR, ^{13}C-NMR and HRMS data of compound 22b

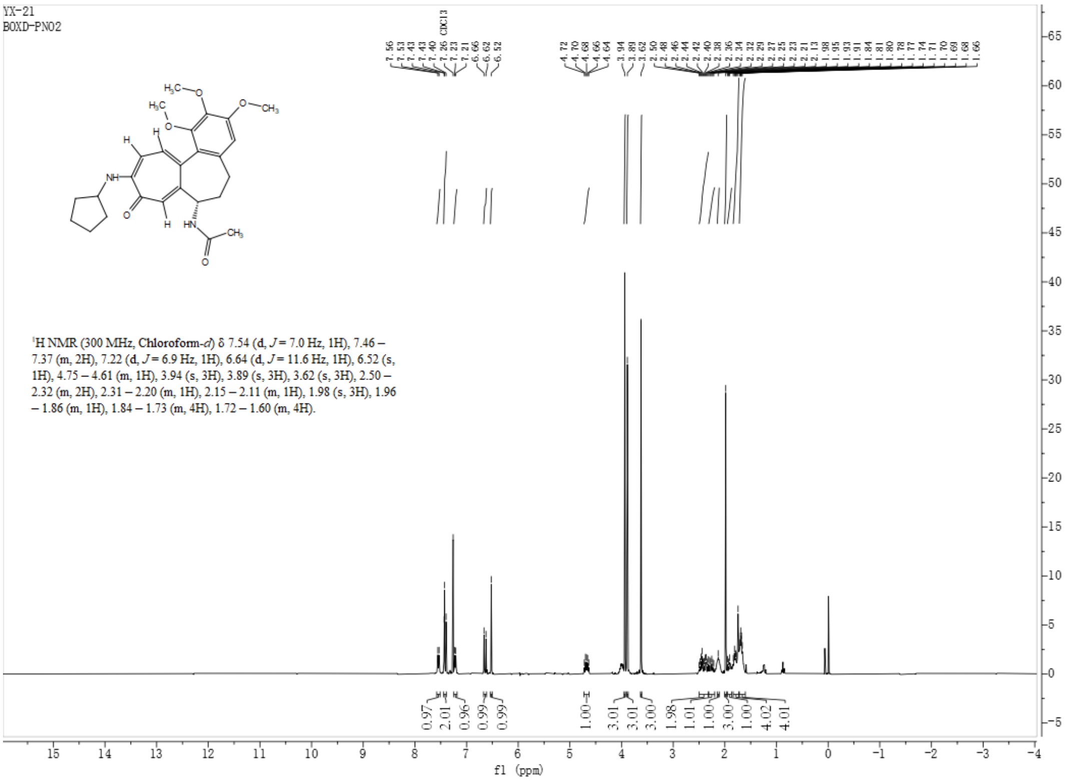

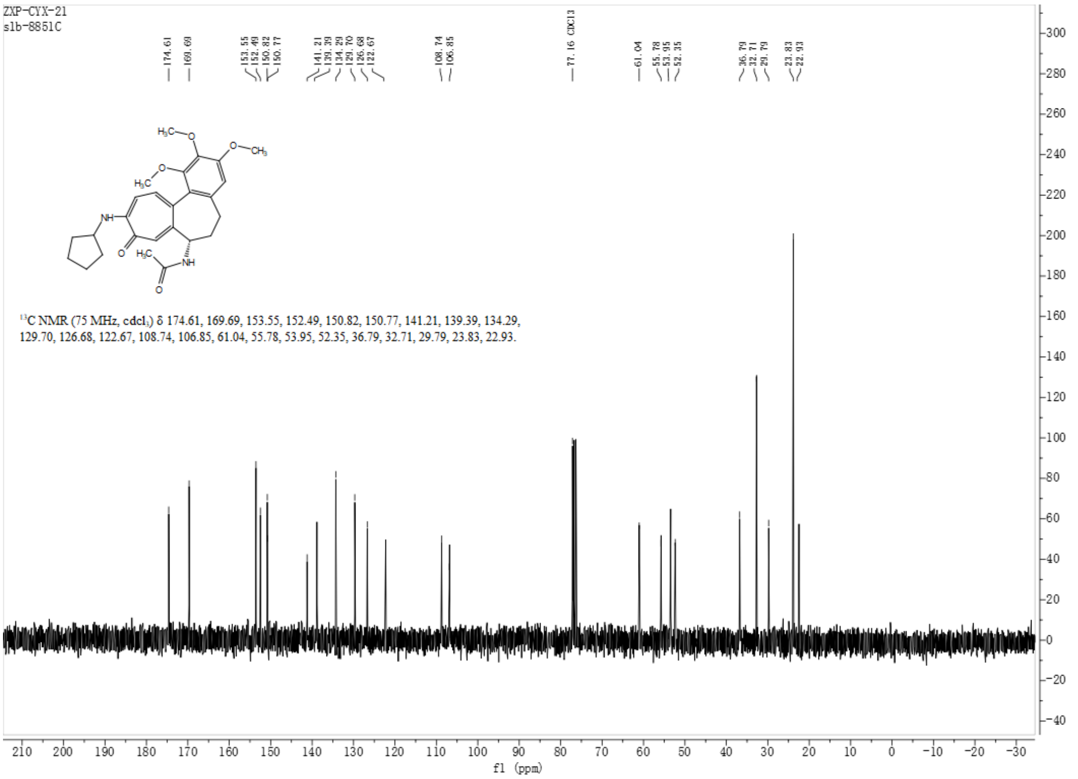
ZXP-CYX-21
slb-8851C
13C NMR (75 MHz, cdcl3) δ 174.61, 169.69, 153.55, 152.49, 150.82, 150.77, 141.21, 139.39, 134.29, 129.70, 126.68, 122.67, 108.74, 106.85, 61.04, 55.78, 53.95, 52.35, 36.79, 32.71, 29.79, 23.83, 22.93.
77.16 CDCl3
f1 (ppm)

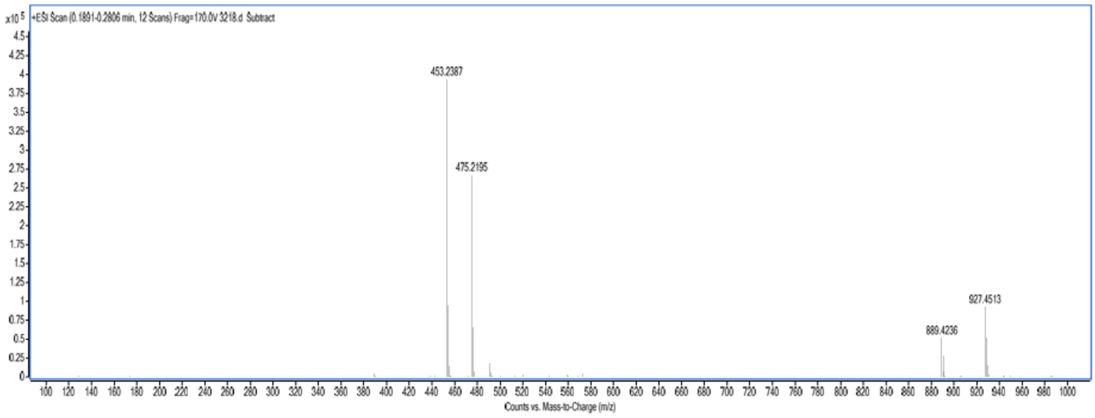
+ESI Scan (0.1801-0.2806 min, 12 Scans) Frag=170.0V 3218.d Subtract
x10 5
453.2387
475.2195
889.4236
927.4513
Counts vs. Mass-to-Charge (m/z)

Figure S23. ^{1}H-NMR, ^{13}C-NMR and HRMS data of compound 23b

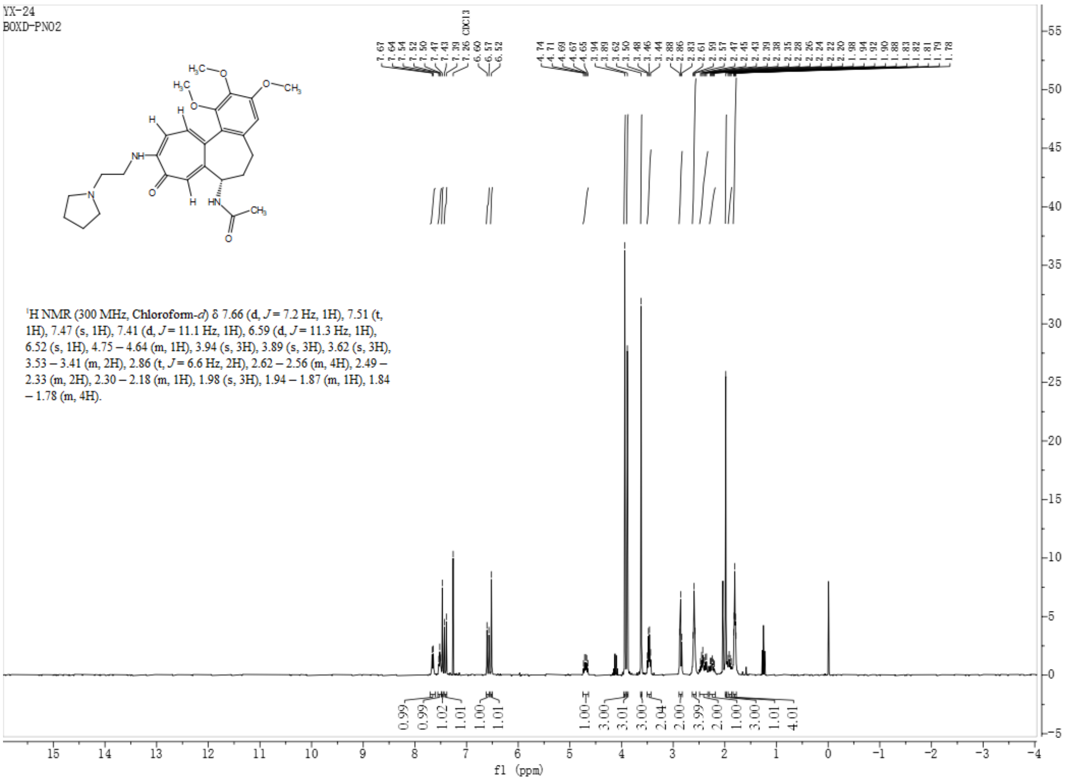

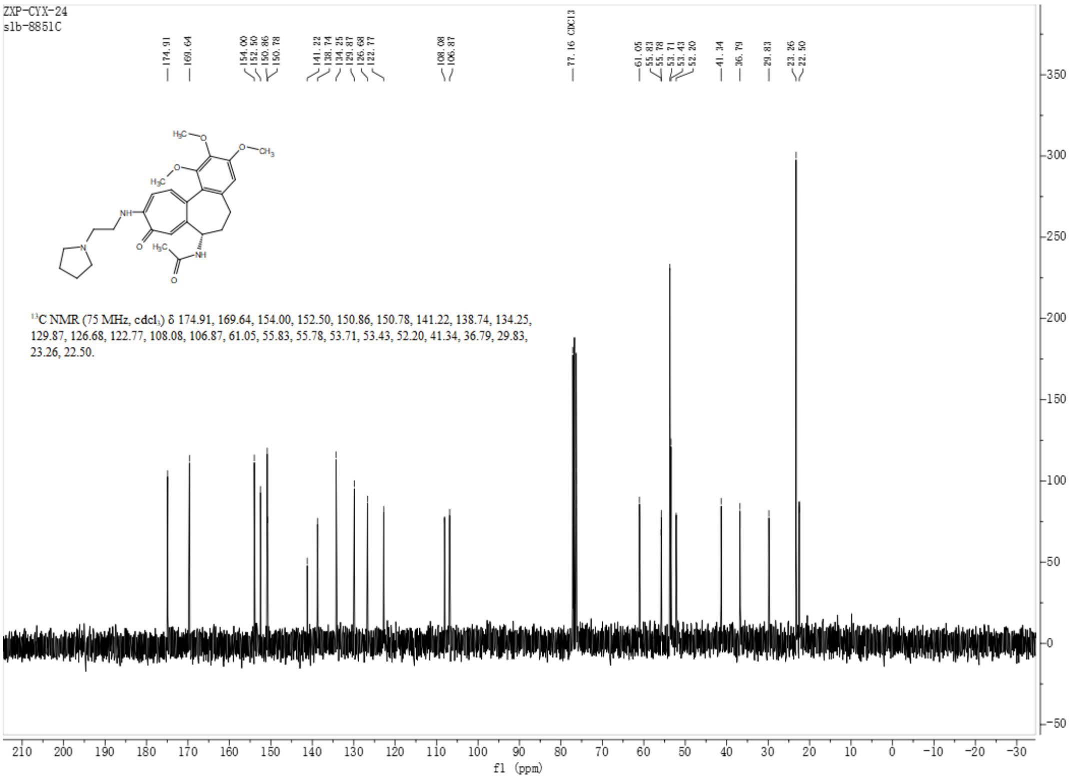

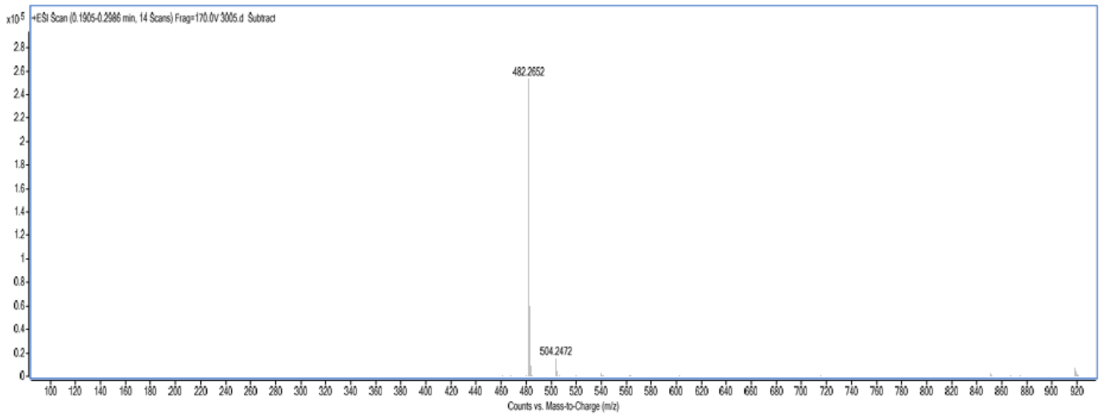

Figure S24. ^{1}H-NMR, ^{13}C-NMR and HRMS data of compound 24b

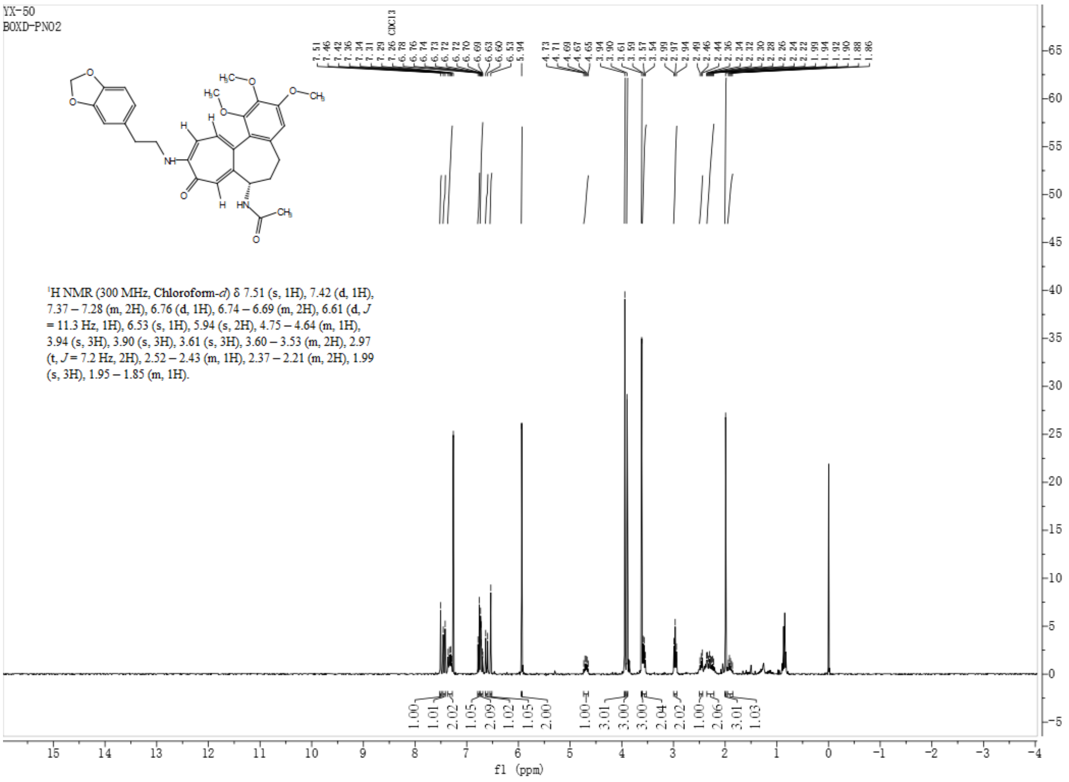

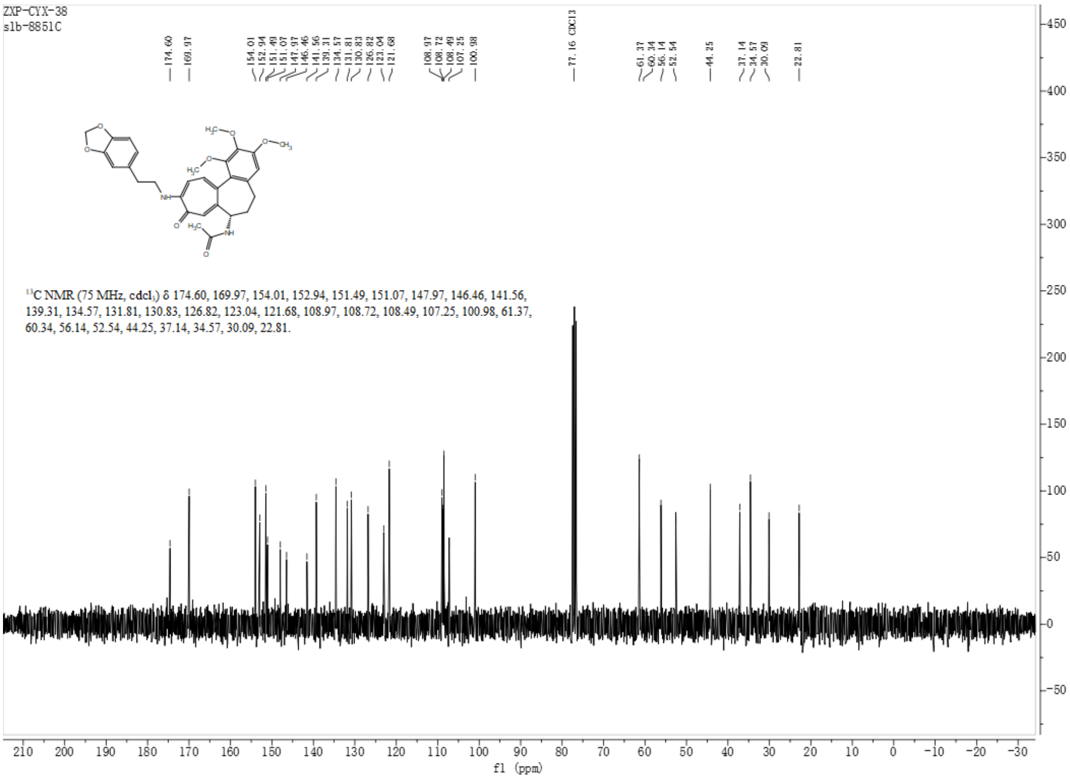
ZXP-CYX-38
slb-8851C
77.16 CDCl3
^{13}C NMR (75 MHz, cdcl$_3$) δ 174.60, 169.97, 154.01, 152.94, 151.49, 151.07, 147.97, 146.46, 141.56, 139.31, 134.57, 131.81, 130.83, 126.82, 123.04, 121.68, 108.97, 108.72, 108.49, 107.25, 100.98, 61.37, 60.34, 56.14, 52.54, 44.25, 37.14, 34.57, 30.09, 22.81.
f1 (ppm)

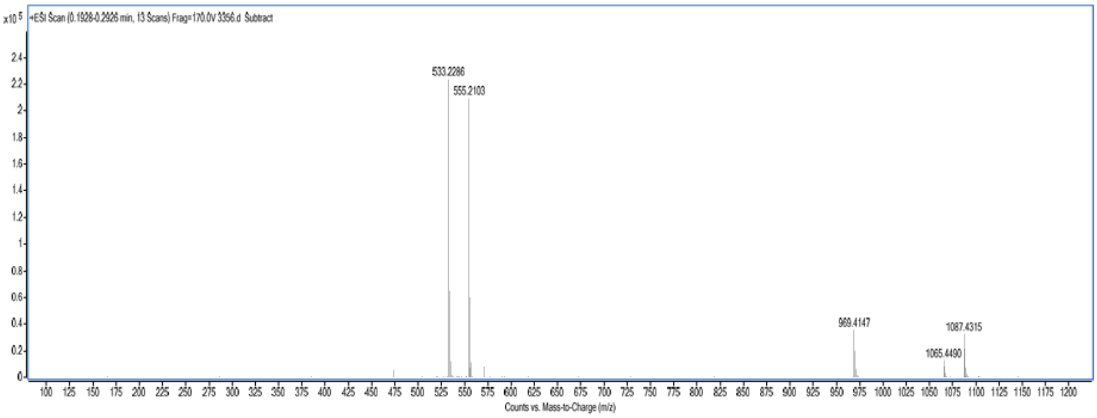
+ESI Scan (0.1528-0.2926 min, 13 Scans) Frag=170.0V 3356.d Subtract
x10 5
533.2286
555.2103
969.4147
1065.4490
1087.4315
Counts vs. Mass-to-Charge (m/z)

Figure S25. ^{1}H-NMR, ^{13}C-NMR and HRMS data of compound 25b

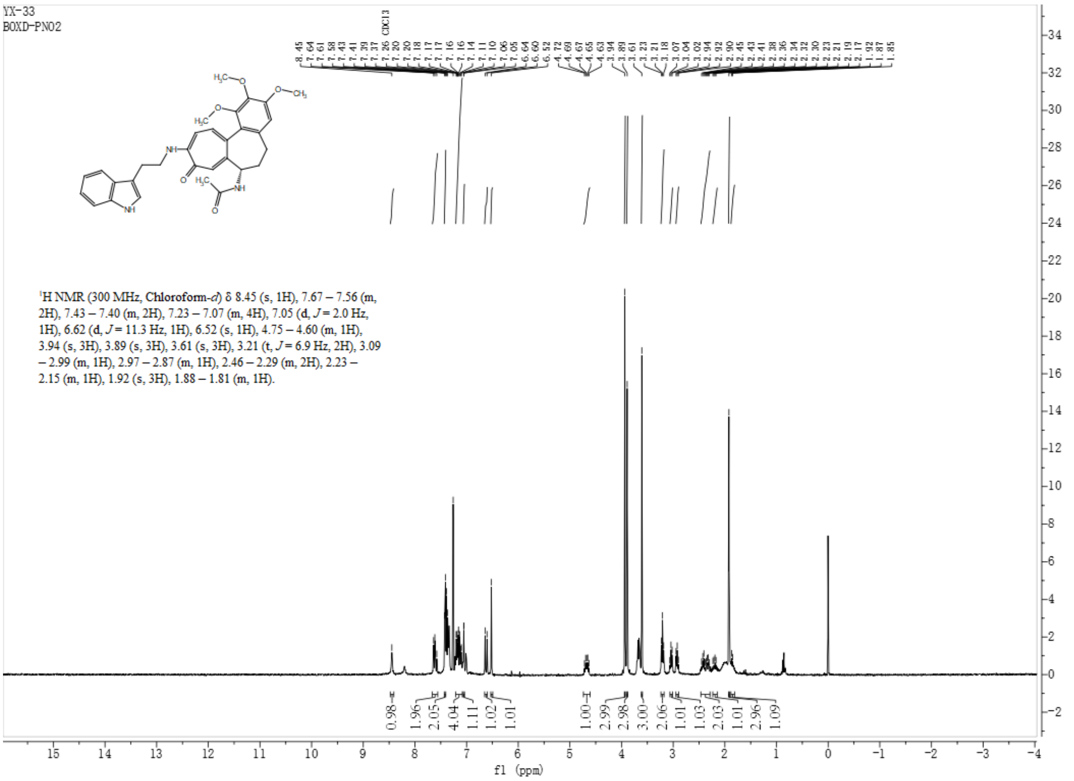

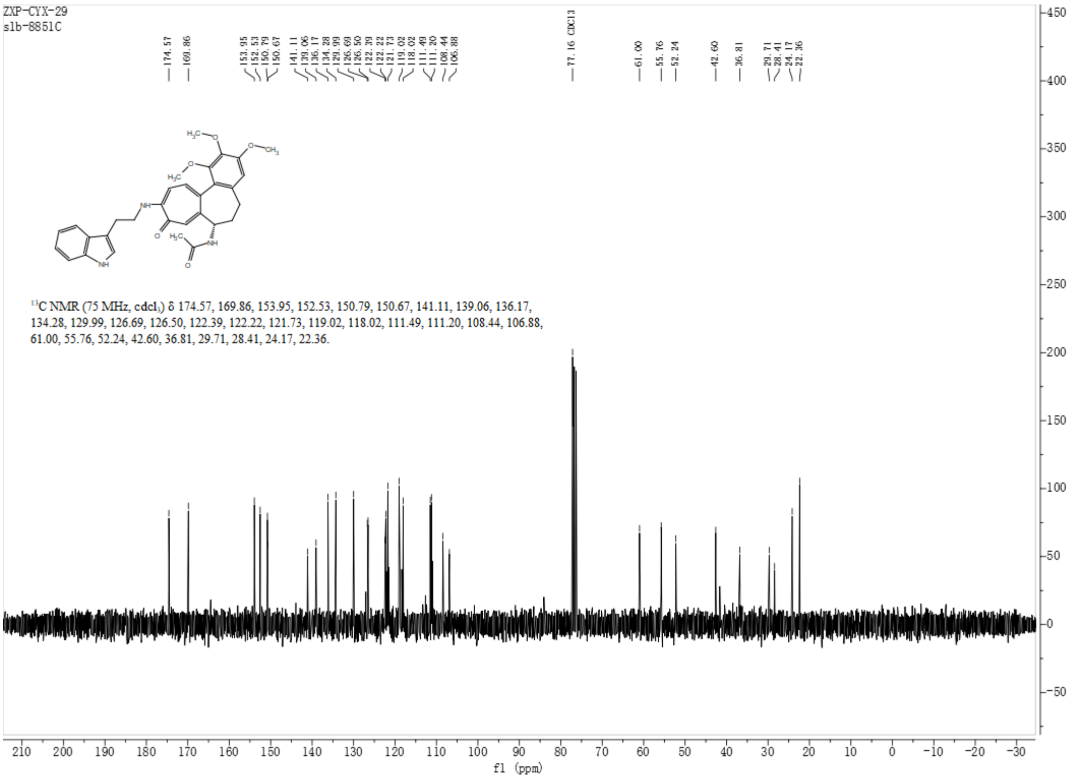

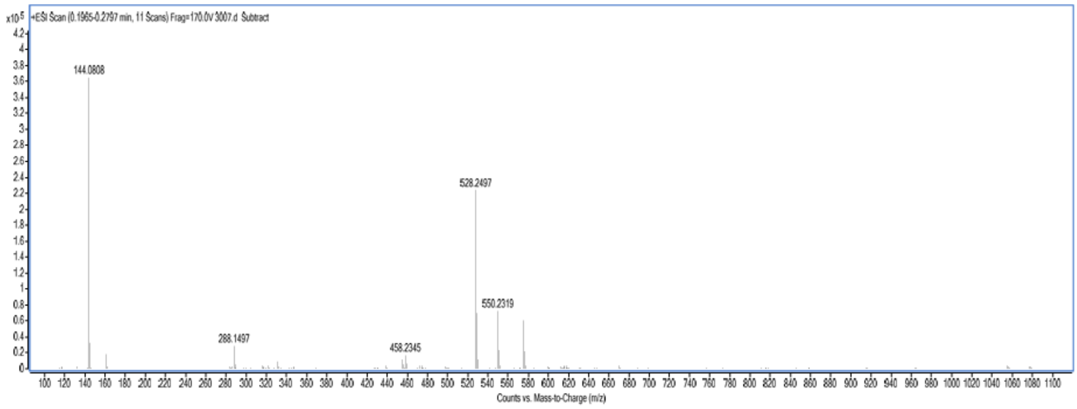

Figure S26. ^{1}H-NMR, ^{13}C-NMR and HRMS data of compound 26b

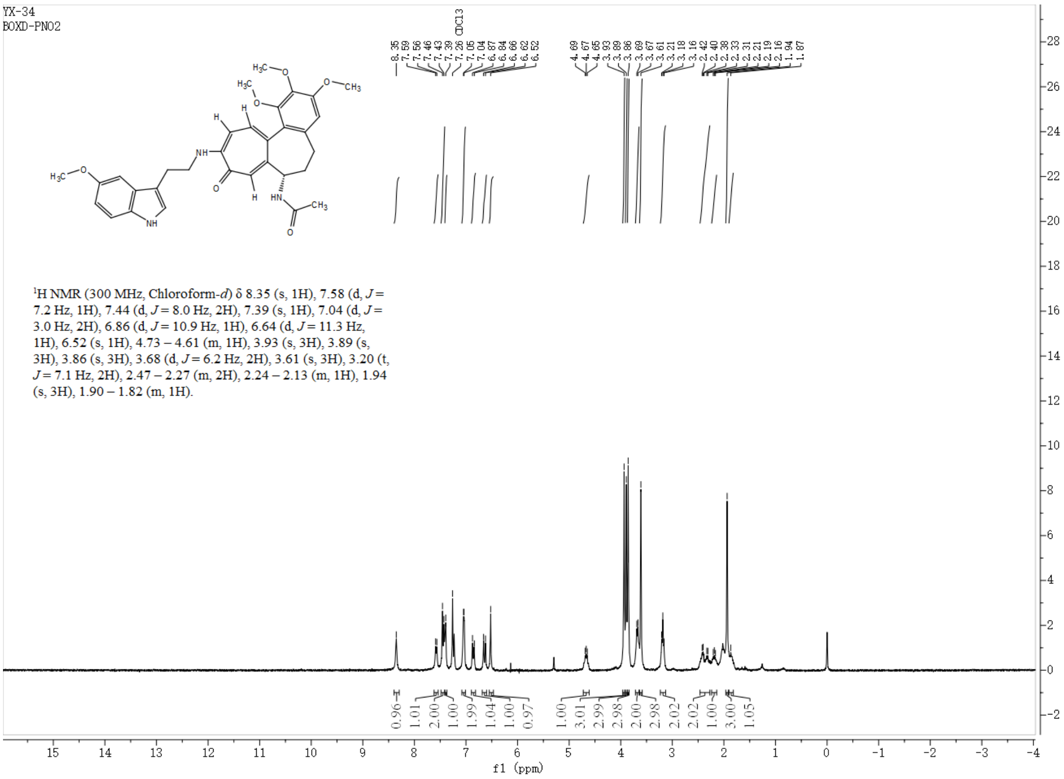

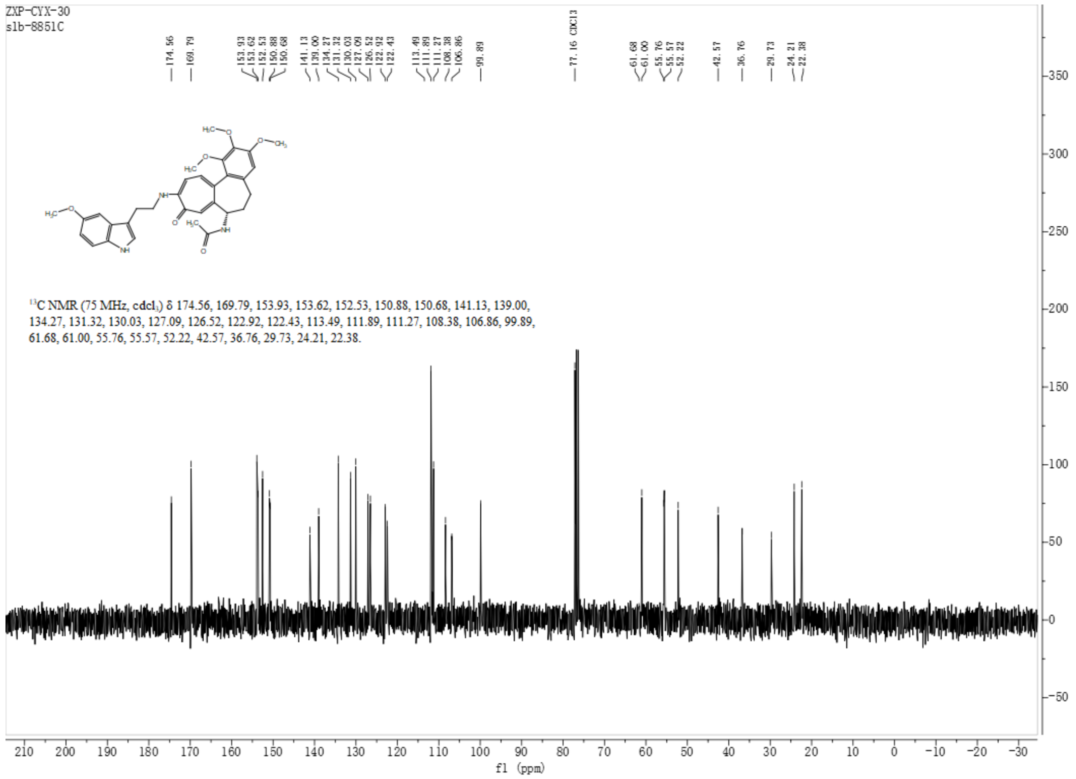
ZXP-CYX-30
slb-8851C
77.16 CDCl3
13C NMR (75 MHz, cdcl3) δ 174.56, 169.79, 153.93, 153.62, 152.53, 150.88, 150.68, 141.13, 139.00, 134.27, 131.32, 130.03, 127.09, 126.52, 122.92, 122.43, 113.49, 111.89, 111.27, 108.38, 106.86, 99.89, 61.68, 61.00, 55.76, 55.57, 52.22, 42.57, 36.76, 29.73, 24.21, 22.38.
f1 (ppm)

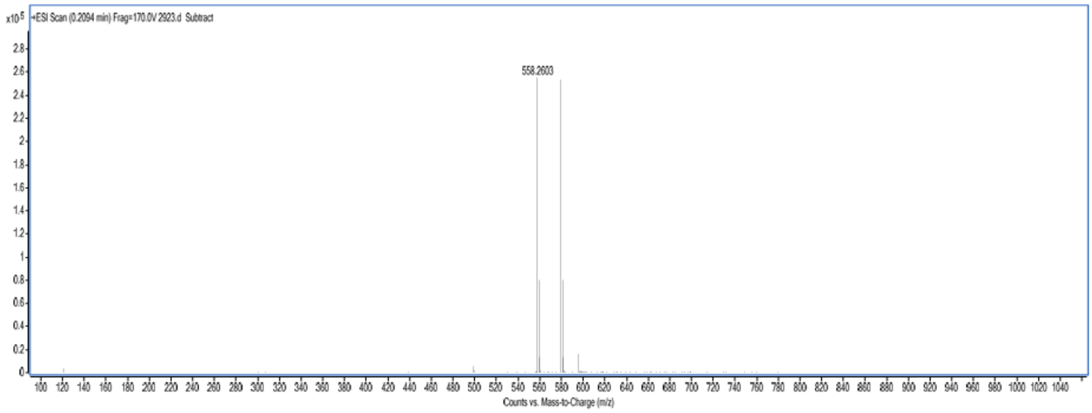
+ESI Scan (0.2094 min) Frag=170.0V 2923.d Subtract
558.2603
Counts vs. Mass-to-Charge (m/z)

Figure S27. ^{1}H-NMR, ^{13}C-NMR and HRMS data of compound 27b

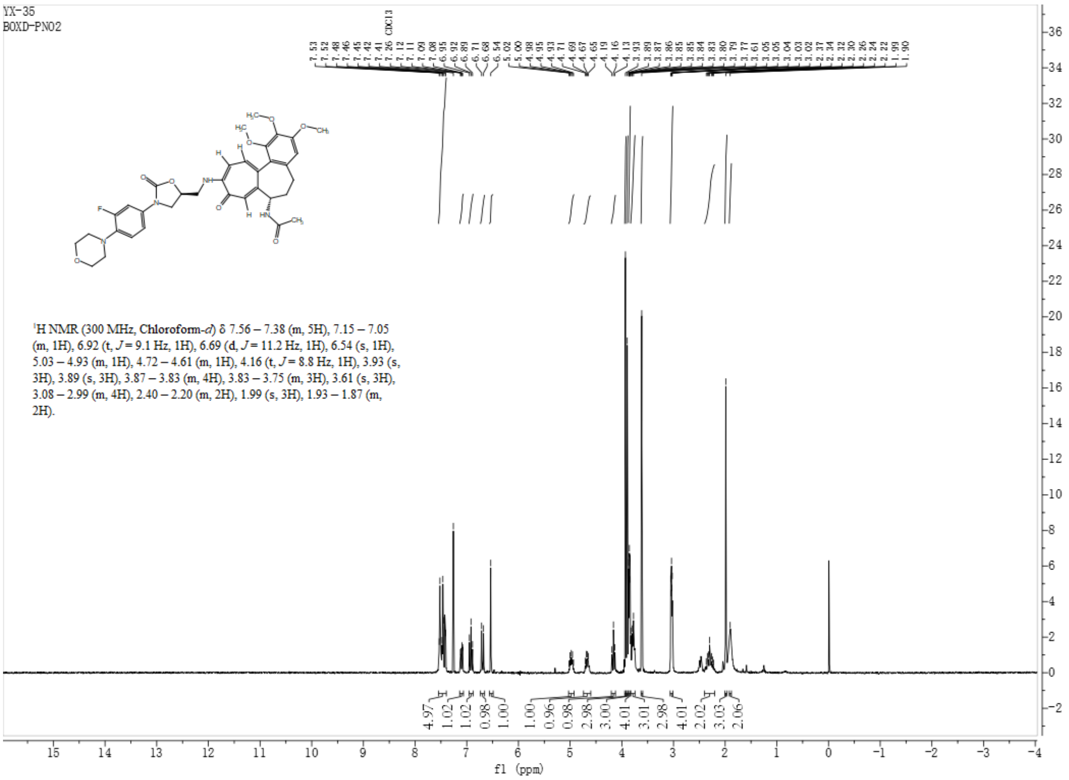

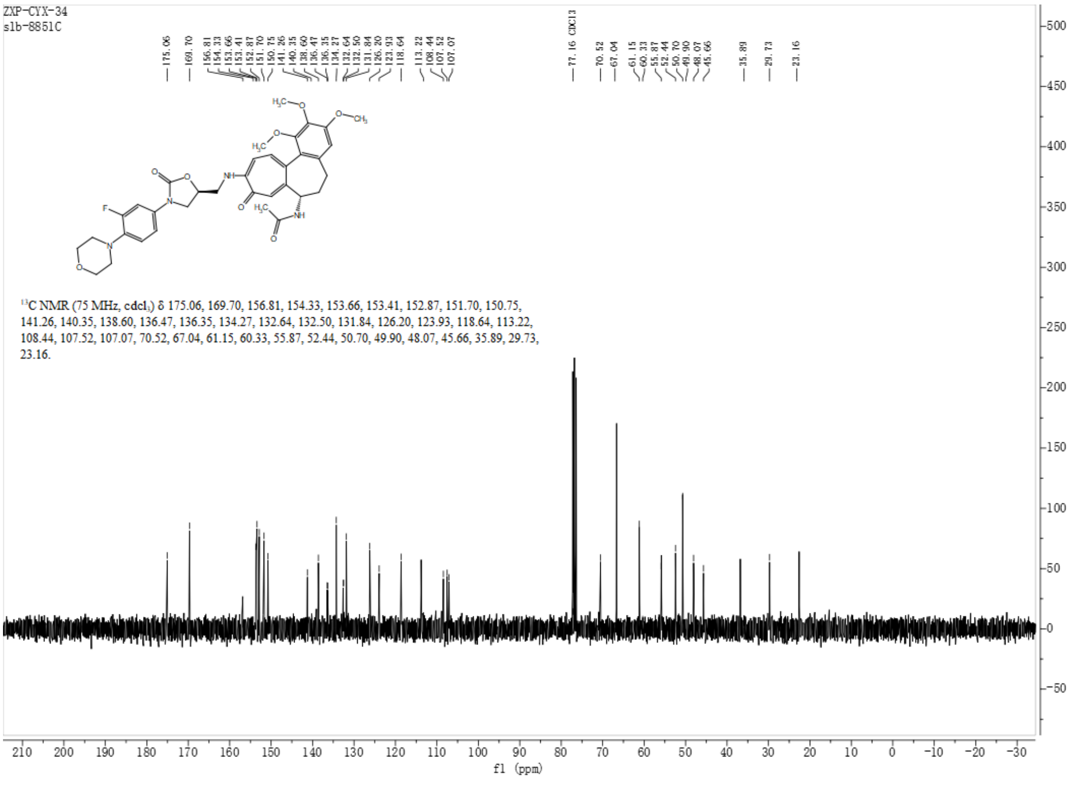

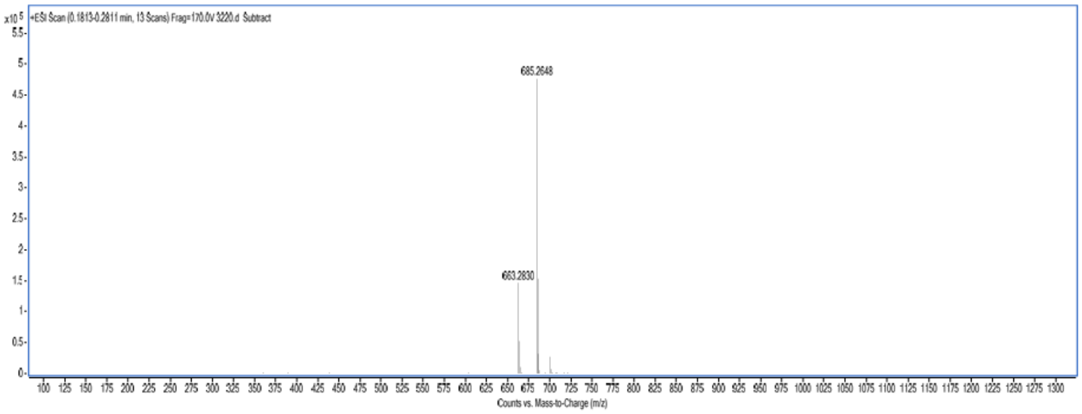

Table S1 Primer information for qPCR analysis of the selected genes

Primer name	Sequences (5′ -3′)
16s-Q-F	TGATCCTGGCTCAGGATGA
16s-Q-R	TTCGCTCGACTTGCATGTA
IcaA -Q-F	CTGGCGCAGTCAATACTATTTCGGGTGTCT
IcaA -Q-R	GACCTCCCAATGTTTCTGGAACCAACATCC
AgrA -Q-F	TGATAATCCTTATGAGGTGCTT
AgrA -Q-R	CAC TGT GAC TCG TAA CGA AAA
